# Hypnotic suggestion versus sensory modulation of bodily awareness

**DOI:** 10.1371/journal.pone.0291493

**Published:** 2023-09-12

**Authors:** C. Apelian, D. B. Terhune, F. De Vignemont

**Affiliations:** 1 Département d’études Cognitives, Institut Jean Nicod, ENS, EHESS, CNRS, PSL University, Paris, France; 2 ARCHE, Formation, Paris, France; 3 Department of Psychology, Goldsmiths, University of London, London, United Kingdom; 4 Department of Psychology, Institute of Psychiatry, Psychology & Neuroscience, King’s College London, London, United Kingdom; Anglia Ruskin University, UNITED KINGDOM

## Abstract

Bodily awareness arises from somatosensory, vestibular, and visual inputs but cannot be reduced to these incoming sensory signals. Cognitive factors are known to also impact bodily awareness, though their specific influence is poorly understood. Here we systematically compared the effects of sensory (bottom-up) and cognitive (top-down) manipulations on the estimated size of body parts. Toward this end, in a repeated-measures design, we sought to induce the illusion that the right index finger was elongating by vibrating the biceps tendon of the left arm whilst participants grasped the tip of their right index finger (Lackner illusion; bottom-up) and separately by hypnotic suggestion (top-down), with a sham version of the Lackner illusion as an active control condition. The effects of these manipulations were assessed with perceptual and motor tasks to capture different components of the representation of body size. We found that hypnotic suggestion significantly induced the illusion in both tasks relative to the sham condition. The magnitudes of these effects were stronger than those in the Lackner illusion condition, which only produced a significantly stronger illusion than the sham condition in the perceptual task. We further observed that illusion magnitude significantly correlated across tasks and conditions, suggesting partly shared mechanisms. These results are in line with theories of separate but interacting representational processes for perception and action and highlight the influence of cognitive factors on low-level body representations.

## Introduction

The experience of the body may seem self-evident, coherent, and stable, to the point of being almost invisible. However, in some circumstances bodily awareness can be dramatically altered. Not only can one experience phantom sensations in long-lost amputated limbs [1], but one can also fail to locate one’s body parts (autotopagnosia) [2], or experience them as being elongated (macrosomatognosia) [3], alien (somatoparaphrenia) [4], or as having a will of their own (anarchic hand syndrome) [5]. Critically, these distortions of bodily awareness are not restricted to psychiatric or neurological disorders and can be experimentally induced in neurotypical individuals through manipulation of sensory inputs [6–8].

The present study focused on body metrics, namely the representation of the length of body parts. Body metrics comprise relatively basic properties, whose accurate representation is essential for action planning [9]. It is also relatively central to the way we perceive ourselves, as distortions of these metrics are known to contribute to disorders such as anorexia nervosa [10, 11]. Yet, body metrics can be manipulated relatively easily. For instance, the vibration of the insertion site of the biceps brachii can elicit the experience of illusory arm extension. If, at the same time, one holds a part of one’s body with the arm that one feels moving away, one can report feeling that the grasped body part is elongating in the direction of the illusory motion (the *Lackner illusion*) [6, 12–17]. One can induce similar body part elongation by altering visual inputs, for example through virtual reality [7].

It is generally assumed that bodily illusions depend on misleading sensory signals [18, 19]. For example, in the Lackner illusion, perceived limb extension is caused by vibrating a tendon, which alters proprioceptive signals. By contrast, the extent to which these illusions are attributable to, or influenced by, top-down factors, has received only scant attention. In contrast to sensory-driven methods, a top-down approach aims to modify bodily awareness by inducing changes in the subject’s beliefs, motivations, and expectations, with outcomes shaped by personal learning history, current social environment, and germane psychological traits [20]. Most experiments adopting this top-down approach target high-level representations of the body. For example, participants are exposed to photographs of thin fashion models, which have been shown to modify bodily satisfaction, as indexed by psychometric measures [21]. A possibly more promising, albeit understudied, route for investigating the role of top-down mechanisms in the modulation of bodily awareness is provided by hypnotic suggestion [22, 23]. It involves direct verbal suggestions following hypnotic induction, which consists of a prior extended suggestion to enter a “state of hypnosis” aimed at raising suggestibility [24–27]. A wealth of data has demonstrated that hypnotic suggestion can be used to impact processes long thought to be beyond social influence. It has thus been shown that hypnotic suggestion can reliably induce analgesia [28], subjective blindness [29], and visual hallucinations [30–32], and that it can reduce response conflict in cognitive control tasks [33]. Hypnotic suggestion has also been used to influence various dimensions of self-awareness, including the sense of agency [34–36], mirror self-recognition [37], sex change delusion [38], and the sense of body ownership [39]. Some of these studies specifically target bodily awareness [40–42] or have used expectation manipulation to modulate body perception [43] but to our knowledge, hypnotic suggestion has not yet been systematically used to modify the relatively low-level property of body metrics.

Bottom-up modulation through distorted sensory signals and top-down modulation through hypnotic suggestion may be conceived as distinct modes by which bodily awareness can be manipulated but it remains an open question to what extent they are fully independent. For example, several studies have reported positive correlations between the magnitude of the rubber hand illusion (RHI) [8], as well as other germane bodily illusions, and (hypnotic) suggestibility [44–48]. However, it should be noted that a correlation between hypnotic suggestibility and a specific bodily illusion does *not* necessarily entail that the illusion is driven by suggestion and expectations. It may merely indicate that highly suggestible individuals are more responsive to the illusion induction method via bottom-up mechanisms. Furthermore, it has been recently debated to what extent the RHI could be explained by demand characteristics [45, 49, 50], that is, by participants’ explicit compliance, or unconscious changes in behaviour and experience in accordance with implicit or explicit experimental demands [51]. These debates highlight the need for a better understanding of top-down influences on bodily awareness. In turn, this will allow us to critically examine current models of body representation and pave the way forward to better understand the diverse factors that shape bodily awareness.

A now classic taxonomy distinguishes between visuospatial aspects of body representation, labelled body image, and sensorimotor aspects of body representation, often referred to under the label of body schema. This distinction raises a number of methodological and theoretical challenges but it can be summarized as follows: body image is the body you perceive, whereas body schema is the body you act with [52]. Here we define body image as the perceptual report of a body part size. Body schema on the other hand is defined here as the perceived distance between the position of a body part and a target one aims for when only proprioception is available to reach that target. Accordingly, bodily awareness can be selectively impaired specifically at one level, and not at the other [53]. In Alice in Wonderland syndrome, for instance, some patients report feeling very tall, as if they were walking on stilts, yet they walk normally [54]. In this case, only body image is disturbed, and body schema remains intact. This dissociation can also be experimentally induced in healthy participants. In several instances, illusions impact perceptual responses considerably more than motor responses [14, 55, 56]. Illusory arm movement due to tendon vibration, for instance, does not significantly affect motor planning [14]. Nonetheless, it is believed that body image and body schema interact in most instances [54, 57, 58], in part because the brain tends to avoid conflict whenever possible and favours unity and consistency. Consequently, in everyday life the body we perceive generally coincides with the body that we act with.

The central aim of this study was to induce the experience of finger elongation and to contrast bottom-up (Lackner illusion) and top-down (hypnotic suggestion) manipulations of both body image and body schema. To measure the effects on these bodily representations, we used two tasks that are considered as reliable behavioural measures of body image and body schema. We assessed body image by using a continuous version of body size estimation [16, 59, 60], in which a participant adjusted a visual representation of their hand where the index finger size is changed using a slider (finger length perception task) and body schema by a line reaching task wherein participants matched the position of different lines with their fingertip hidden inside of a box [14, 61]. First, we sought to determine whether hypnotic suggestion can effectively modulate body image and/or body schema. Secondly, we compared the magnitude of such effects against corresponding bottom-up influences on these representations (Lackner illusion) and an active sham condition. Since models of body image and body schema are mostly informed by bottom-up manipulations, our third aim was to assess the robustness of these models when cognitive factors were manipulated. More precisely, we wanted to clarify the interaction of body image and body schema, as reflected in correlations of their respective measures under different sets of constraints, either sensory (Lackner illusion) or cognitive (hypnotic suggestion). Insofar as responsiveness to direct verbal suggestions varies considerably in the general population [20], we measured hypnotic suggestibility and expected that it would predict the magnitude of the hypnotic suggestion effect.

## Material and methods

We report how we determined our sample size, all data exclusions, all inclusion/exclusion criteria, whether inclusion/exclusion criteria were established prior to data analysis, all manipulations, and all measures in the study.

### Participants

The sample size, exclusion criteria, manipulations and measures were established prior to data acquisition. We specified the *a priori* criterion that a mean effect compared to the sham condition below the test-retest reliability of our measurements would not be considered meaningful. A pilot study led us to estimate this minimal effect at 10 mm, corresponding to an effect size of (Hedges’s) *g* = .53. Using a statistical power estimate (1-β) of 0.95 and an α-level of .05 with a two-tailed paired-samples *t*-test, a power analysis run using GPower 3.1 [62] yielded a minimum sample size of 49 participants. To account for potential outliers and/or attrition, we pre-specified a sample size of 52, allowing for around 5% of data loss. 51 participants (31 women, 20 men) completed the experiment (*M*_age_ = 38.7; *SD* = 13.2; range: 20–65 years). All but two were right-handed according to the Edinburgh Handedness Inventory [63]. None reported psychiatric or neurologic disorders nor use of psychoactive drugs (medical or recreational) in the past six months. All participants had two valid, functional arms and were fluent French speakers. 27 participants had never experienced hypnosis, 11 reported having experienced hypnosis in the past at least once, and 13 were current hypnosis users (self-hypnosis, counselling, therapy). Participants were recruited *via* two communication networks. One was the RISC (Relais d’Information sur les Sciences de la Cognition; *RISC*-UAR 3332 CNRS), a service advertising experiment opportunities in cognitive science to potential participants. The second one was the first author’s personal communication network. The sample was diverse in age, gender, ethnicity and prior knowledge of hypnosis. The whole experiment was approved by the ethics committee of Paris Descartes under IRB n°00012019–19 in agreement with the Declaration of Helsinki (2008). Informed written consent was obtained from each participant before the beginning of the study.

### Materials

Social desirability was indexed using the *Balanced Inventory of Desirable Responding* short form (BIDR-16) [64]. The BIDR-16 consists of sixteen items in which participants rate the extent to which different statements apply to them using a 7-point Likert scale. This scale includes two subscales: self-deceptive enhancement, corresponding to the tendency to give honest but positively-biased reports, and image management, corresponding to conscious dissimulation of responses in order to give a socially-favourable image of oneself. These subscales have good test-retest reliability and correlate modestly. In our sample, the self-deceptive enhancement subscale displayed acceptable internal consistency (Cronbach’s α = 0.74) whereas the image management subscale did not (α = 0.52).

Hypnotic suggestibility was measured using the French version of the *Waterloo-Stanford Group Scale of Hypnotic Susceptibility*: *Form C* (WSGC) [65, 66]. This scale consists of a relaxation-based hypnotic induction followed by twelve suggestions for alterations in motor control, cognition, and perception. Following a de-induction, participants dichotomously self-rate their responsiveness to each of the suggestions. Hypnotic suggestibility is subsequently quantified as the total score (range: 0 to 12). The WSGC is a widely-used measure of hypnotic suggestibility that has previously been found to exhibit acceptable internal consistency [66, 67] although it was borderline in the present sample (α = 0.61), according to commonly used conventions [68]. In addition to hypnotic suggestibility, we asked participants to report their familiarity with hypnosis with the question “Report your familiarity with hypnosis (being hypnotized) or auto-hypnosis” using a Likert 5-point scale (1: “None” to 5: “Daily”).

**Fig 1:**
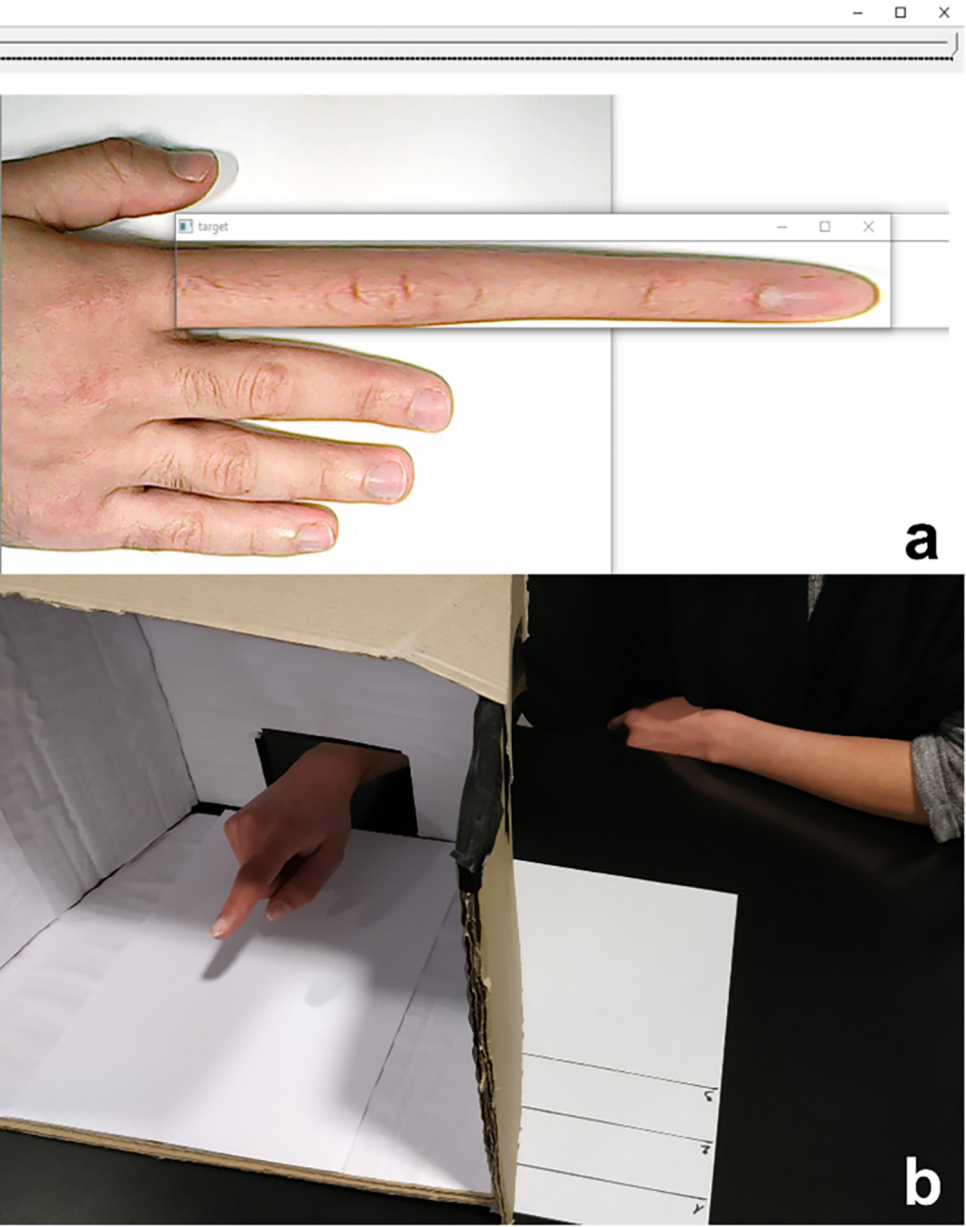
Experimental apparatuses in the finger length perception task (a) and line reaching task (b). (a) Participants adjusted the slider so that the graphical representation matched their perceived finger length. As the slider is moved, the target window is resized to change the displayed length of the finger. The window is set to enclose only the index finger and no other element that could give away the true finger size. (b) Participants hovered above the estimated location of one of three parallel lines with their right index fingertip whilst their hand was hidden from view in a box. The position of the right hand in (b) corresponds to the starting position in each trial; the left hand simply rests on the table during measurements.

A *finger length perception task*, a continuous version of body size estimation [16, 59, 60], was used to measure body image. The task was performed using a graphical user interface displaying a picture of the participant’s right hand and a slider (Fig 1). This GUI was presented on a laptop (Lenovo ideapad 100S; screen size: 14”). A picture of the participant’s left hand was taken at the start of the experiment against a white backdrop with ambient light from above (no directional light to prevent hard shadows) using a web camera (F/#2.0; f: 4.8mm - ∞). The GUI was developed by the first author using python and the OpenCV library (available upon request). The “target” window (Fig 1A) separating the index finger from the rest of the hand was positioned and sized by the experimenter at the beginning of the experiment, just after the picture of the hand was taken. The participant was instructed to adjust the slider until the picture of their index finger matched their perceived length of their finger. Adjusting the slider resized the “target” window laterally, thus shrinking or expanding the displayed length of the finger. In each condition, perceived finger length was measured as the mean of two trials, one with the starting position of the slider at the maximum distortion (three times the normal size) and one starting at the minimum distortion (one third of the normal size). This was done to prevent anchoring effects [69]. By comparing the reported finger size at baseline to the actual finger size, we can derive an estimate of the bias in the measurement. In this study, a mean bias of 9.6mm (*SD* = 7.4) was found, indicating that participants overestimated the size of their index finger. This motivated us to use the mean bias as the dependent measure for this task, which was operationalized as the difference between the perceived finger length in each experimental condition relative to baseline, with negative and positive values denoting perceptual contraction and elongation, respectively. We refer to this bias measure as the *perceptual effect*.

A *line reaching task* was used to measure body schema, similar to classic reaching or matching tasks [14, 61]. In this task, the participant’s right arm was hidden in a box on a table before removing their blindfold. Three parallel lines {1,2,3} with an inter-line distance of 40mm were displayed in the field of view of the participant next to the box, with a number displayed next to each line (Fig 1). In each condition, participants completed seven trials in which they were asked to match the position of one of the three parallel lines with their right index fingertip {1,2,3,2,1,3,1}. The lines were only visible to the participants outside the box, so they could not see the position of their right hand. Position recording was performed by placing a sheet of paper at a standard position at the bottom of the box and marking down the position of the fingertip with a marker for each trial. Positions were extracted by measuring the distance between the marks and the edge of the sheet. When comparing the difference of finger positions at baseline relative to the target lines positions, the mean variation was 6mm (*SD* = 18.9mm), again indicating participants’ tendency to overestimate their fingertip distance (resulting in undershooting). Hence, as in the finger perception task, we used the mean bias in our analyses, computed as the mean finger position in each experimental condition relative to the mean finger position at baseline with positive values corresponding to undershooting (pointing closer to the body relative to the baseline trial) and negative values corresponding to overshooting (pointing further away from the body relative to the baseline trial). We refer to this bias measure as the *pointing effect*. Given the lack of an a priori expectation regarding the direction of the effect on this task and the difficulty participants reported to consciously locate their fingertip, this task played the role of an implicit measure of body schema.

### Procedure

#### Lackner illusion

**Fig 2:**
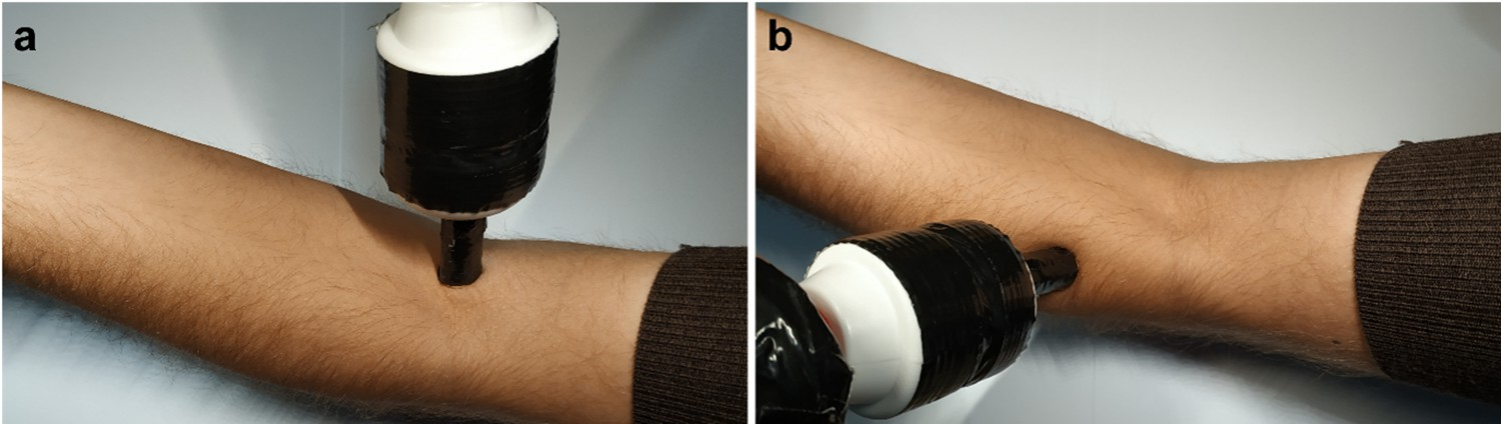
Depiction of the stimulation point for the (a) Lackner illusion procedure and (b) sham. (a) The stimulation was applied to the insertion site of the biceps brachii, while (b) stimulation on the brachio-radialis. In this figure the arm is open for clearly displaying the stimulation landmarks, however in the experiment, the participant’s left arm was set at a 90° angle.

In the Lackner illusion condition we vibrated (80Hz ± 10Hz) the tendon at the insertion site of the left biceps brachii for 30s (Fig 2) whilst the participant, blindfolded, held their right-hand index finger with their left hand in a pincer grip. This frequency and duration of stimulation has previously been shown to be optimal in the induction of the illusion [12]. The vibrating device used in this study was a handheld wand massager (CE; Paloqueth). The participant’s left arm was set at a 90° angle and remained stationary for the whole duration of the condition. Participants were informed that they might feel their right index finger changing in size or not, but no reference was made regarding whether it might contract or elongate. Insofar as the Lackner illusion persists after vibration has stopped, the illusion was tested in the same way as other conditions, without vibration or index finger being held by the contralateral hand.

#### Sham

In the sham condition, all instructions were the same as the Lackner illusion procedure, except the vibration was applied to the skin next to the tendon (~4cm distance) on the brachio-radialis where no physiological effect should arise [6, 16]. To ensure that expectations were similar between the sham manipulation and the Lackner illusion manipulation, we surveyed a group of 72 participants (online) that did not know of the Lackner illusion. Participants were described the manipulations and measurements with text and images, but the expected effect of the manipulation was not mentioned similarly to the actual study. Manipulations were labelled as “illusion 1” (sham) and “illusion 2” (Lackner illusion) for participants. Participants were then asked to estimate if the manipulation, compared to baseline, would make a person receiving the illusion “overshoot, pointing past the lines”, “correctly point the lines”, or “undershoot, being short of reaching the lines”. This was done for both manipulations in the same order as the experiment, with the sham manipulation first then the Lackner illusion manipulation. Similar questions were asked about the effects in the finger length perception task with options: “perceive the index finger shorter”, “perceive the index finger as normal”, “perceive the index finger longer”. Analyses reveal that for the line reaching task expectancies are balanced between manipulations as proportion *P* = .465 was not significantly different from .5 with *p* = .635, *B* = 0.13 (See Table 1). However, this was not the case for the perceptual task where the sham manipulation was expected to lead to perceptual elongation more than the Lackner illusion, proportion *P* = .333 was significantly different from .5 with *p* = .007, *B* = 4.45 (See Table 1). Overall, we are confident that the sham manipulation played its role as a control condition.

**Table 1:** Number of participants expecting each combination of manipulation effect direction in an online survey (N = 72).

	Line reaching task				Finger length perception task		
	Lackner illusion leads to undershooting	Lackner illusion has no effect	Lackner illusion leads to overshooting		Lackner illusion leads to shorter perceived index	Lackner illusion has no effect	Lackner illusion leads to longer perceived index
Sham leads to undershooting	2	4	13	Sham leads to shorter perceived index	3	1	8
Sham has no effect	2	19	3	Sham has no effect	0	19	4
Sham leads to overshooting	17	4	8	Sham leads to longer perceived index	19	7	11

#### Hypnotic suggestion

The hypnotic suggestion condition was conducted following a script developed for this experiment and included an induction followed by a suggestion to experience the right index finger elongating. The induction consisted of focusing attention on the bodily sensations felt in the hands, reducing agency for hand motion (similar to the item “moving hands together” in the WSGC [67]), and in using imagery to change the sensation experienced in the hands, thus fostering a positive set of attitudes toward hypnosis and a passive response set. The hypnotic suggestion was phrased as “the finger is being pulled and elongated”. This suggestion was repeated three times and participant was given 60s to experience the suggestion without further suggestions or cues. This was done to allow for optimal responding, as the perceived effect of the suggestion can take several seconds and often a couple of minutes to peak (see e.g., ref. [70]). Afterwards, and before testing, participants were told that they would go through the measurement tasks, eyes open, while remaining hypnotized with their index finger keeping the elongated size for the whole duration of the measurements. When testing was complete, a standard de-induction was performed (a short procedure aimed at reorienting participants to normal functioning by signing the end of the hypnotic procedure, similar to ref. [67]) with an emphasis on the index finger returning to its normal size. Participants where then instructed to rub their hands and take a short break.

#### General procedure

Participants’ involvement in this study consisted of two sessions separated by at least one day. The first session involved the administration of the French version of the WSGC in small groups (3–8) in a small, quiet classroom without windows or distractors. In the second session, participants completed the finger length perception task and the line reaching task four times: at baseline without manipulation (baseline); after vibrating an irrelevant location near the participant’s elbow (sham); after vibrating the insertion site of the biceps brachii (Lackner illusion); and after a hypnotic induction and a verbal suggestion for finger elongation (hypnotic suggestion). In all four conditions–except baseline–the participants held their right index finger with their left hand in a pincer grip whilst blindfolded. Baseline measurements were performed first, allowing the participant to develop familiarity with the tasks. The sham condition always preceded the Lackner illusion procedure so that participants had no reference level concerning the real illusion. The order of the sham and Lackner illusion conditions, and hypnotic suggestion condition were counterbalanced. Participants were told that the illusions were independent so that they might experience the first one only, the second one only, neither, or both. In each condition, the line reaching task always preceded the finger length perception task. At the end of the experiment, participants were debriefed regarding the sham condition and explained the motivation for this deception. No participant reported being aware that one of the conditions was a sham. A question-and-answer time was offered to dissipate any uneasy feelings that might arise due to the unusual experience of hypnosis. Participants were compensated 16€ for their time.

#### Analyses

In each task, we quantified finger elongation by subtracting the baseline score from the mean score in the respective condition. A single multivariate outlier (>*M*+3 *SD*s in 3 conditions) was identified and removed from the dataset. We tested the effect of each condition on each task relative to baseline with a repeated measures ANOVA followed by *post hoc* analyses, employing two-tailed paired-samples *t*-tests (both frequentist and Bayesian). In *post hoc* tests, the Holm-Bonferroni correction was applied to *p* values. We also computed and compared the correlations (Pearson’s *r*) between perceptual and pointing effects in the different conditions using the cocor library [71]. In Bayesian *post hoc* tests, we used default priors corresponding to a Cauchy distribution centred on zero with a width of .707 (i.e., 1/√2) [72]. For correlations, we also used default priors, corresponding to a stretched beta distribution with width 1.0 [72]. These analyses were performed using JASP [73]. We set alpha error probability thresholds at .05, with *p*-values below this threshold interpreted as statistically significant. Bayes Factors (*B*_10_) below .33 were interpreted to reflect moderate evidence in favour of the null hypothesis, those greater than 3.0 were taken to provide moderate evidence for the alternative hypothesis and in-between values were interpreted as being ambiguous [72]. When possible, we report robustness regions (*RR*) alongside Bayesian analyses by indicating the range of prior widths (either the scale of the Cauchy distribution for t-tests or the stretched Beta distribution width for correlations) consistent with the conclusion derived from the Bayes factor (eg. for a Bayes factor *B* = 10, we report the smallest and largest prior widths for which *B*>3). Hence, no *RR* are reported for *B* between 1/3 and 3. Prior width were varied from 0 to 2, which covers the reasonable range of prior distributions. We use *p*-values, effect sizes, and *B*s for interpreting significant effects but only effect sizes and *B*s for interpreting non-significant effects. Figures were generated using Python 3, and the matplotlib and seaborn libraries [74]. Data are publicly available on the Open Science Framework (https://osf.io/jxek3/). No part of the study procedures or analyses was pre-registered prior to the research being conducted and thus the analyses can be considered exploratory.

## Results

### Sample description

The distribution of hypnotic suggestibility in our sample, as indexed by the WSGC, *M* = 5.65; *SD* = 2.4; range: 0–11, was commensurate with the general Francophone population (*M* = 4.84; *SD* = 2.15; range: 1–10 [66]). Our sample included 8 low (score ≤ 3) and 8 high (score ≥ 9) suggestible participants. Body mass index (kg.m^-2^), *M*_BMI_ = 22; *SD* = 3.3; range: [17.4–36.6], was comparable to the general French population, *M*_BMI_ = 24.8; *SD* = 5.4 [75]. Social desirability, *M*_SDE_ = 4.32; *SD* = 0.97, *M*_IM_ = 4.63; *SD* = 0.78, was also commensurate with previously reported samples [64].

### Condition differences on perceived finger elongation

Our first analysis contrasted the magnitude of the condition manipulations on responses in the two tasks. To that end, we performed a repeated measures one-way ANOVA for testing the influence of condition (baseline, sham, Lackner illusion, hypnotic suggestion) on the outcome measure for each task separately. We found significant main effects of condition in both the perceptual task, *F*(3,49) = 28.9, *p* < .001, *η* ^*2*^ = 0.37, *B*_*10*_ = 3.2 x 10^12^, *RR* = [<0.001, >2], and the line reaching task, *F*(3,49) = 19.6, *p* < .001, *η*^*2*^ = 0.29, *B*_*10*_ = 1.2 x 10^8^, *RR* = [<0.001, >2]. Mauchly’s test of sphericity indicated that the assumption of sphericity was violated (*p* < .05). Nonetheless, applying either the Greenhouse-Geisser or Huynh-Feldt correction did not change the reported results. Hence, the results demonstrate that responses on both tasks varied across conditions.

**Fig 3:**
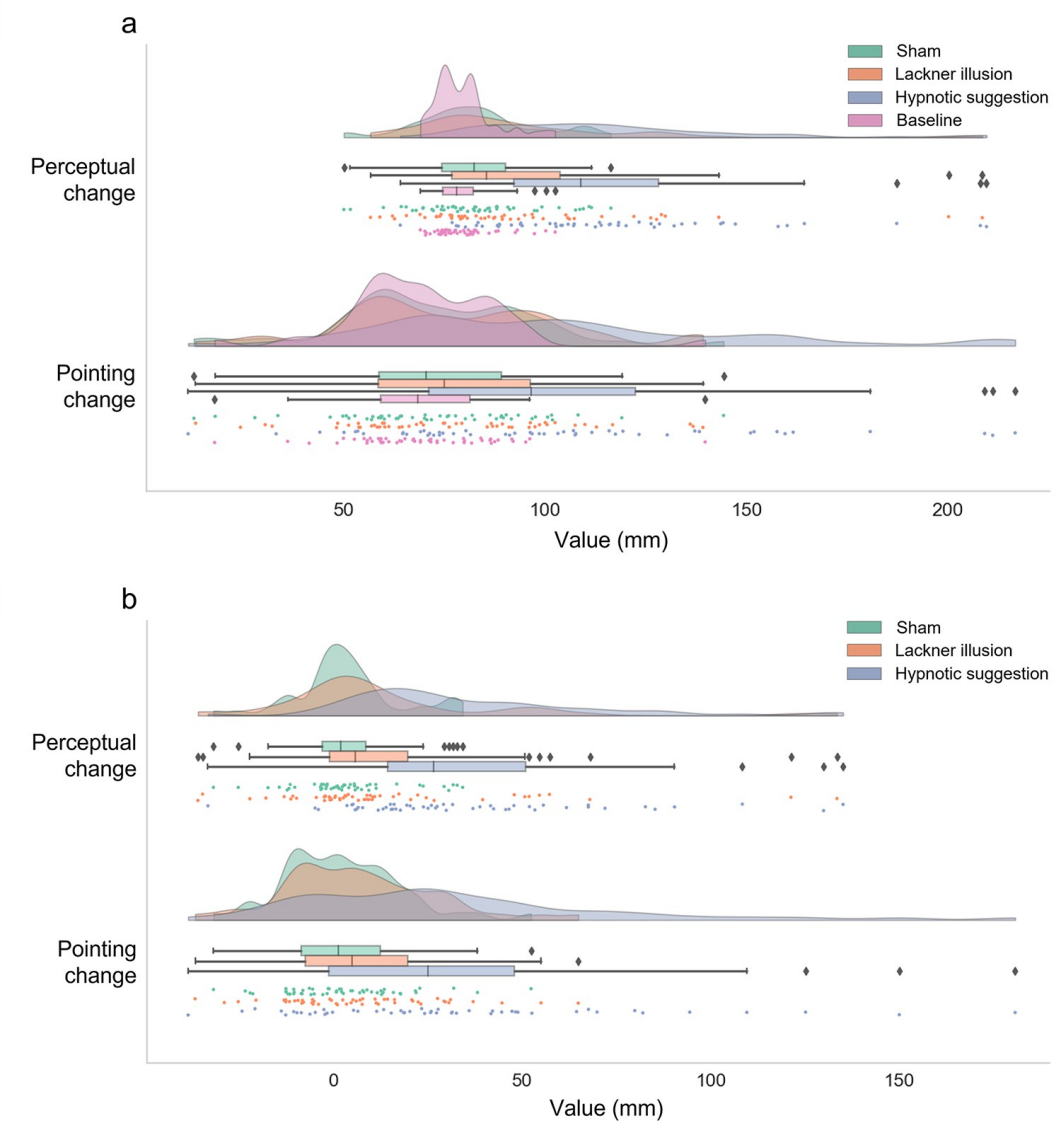
Raincloud plots of perceptual and pointing effects (mm) as a function of condition (*N* = 50). Distributions reflect kernel density estimation plots and markers reflect individual measurements. Boxplot whisker limits are at 1.5 interquartile range from the hinges, diamonds represent individual values outside the whiskers. (a) raw data, (b) baseline corrected data.

As can be seen in Fig 3, Tables 2 and 3, *post hoc t*-tests revealed that the sham condition did not have a significant effect compared to baseline in either task. However, corresponding Bayesian analyses provided evidence in favour of the null hypothesis (no condition difference) only for the line reaching task with only ambiguous evidence for the length perception task. The Lackner illusion procedure had a moderate significant effect (with corresponding Bayesian evidence) on responses in the perceptual task, but no significant effect could be detected on the line reaching task, with corresponding insensitive Bayesian evidence. Similarly, relative to the sham condition, the Lackner illusion only produced a significant effect on responses in the length perception task, although the Bayesian evidence for this difference was insensitive. Responses in the line reaching task also did not significantly differ between the sham and Lackner illusion conditions and the Bayesian evidence was insensitive as to a difference between these conditions. By comparison, relative to both baseline and the sham condition, the hypnotic suggestion condition yielded reliably strong, significant effects (with corresponding Bayesian evidence) on responses in both tasks. Finally, the hypnotic suggestion condition had a strong significant effect on both tasks compared to the Lackner illusion procedure, with corresponding Bayesian evidence in favour of a condition difference. Overall, 42 out of 50 (84%) participants had a stronger effect in the hypnotic suggestion condition compared to the Lackner illusion procedure in the perceptual task, and 40 (80%) in the line reaching task. These results demonstrate that the Lackner illusion manipulation produced a moderate-sized illusion of finger elongation relative to the sham procedure, when indexed with the perceptual effect, but this procedure did not produce robust responses relative to the sham procedure nor reliable effects in the line reaching task. Hypnotic suggestion, on the other hand yielded reliably strong effects on both tasks relative to baseline, sham, and the Lackner illusion procedure.

**Table 2:** Post hoc comparison of the effect of conditions on responses in the finger length perception task (*N* = 50).

Finger length perception task		Mean difference ^a^	*SE*	Cohen’s *d*	*p* ^b^	B_10_	*RR*
Baseline	Sham	3.37	4.21	0.11	.425	0.60	[1.407, >2]
	**Lackner illusion**	**13.95**	**4.21**	**0.47**	**.003**	**9.48**	[0.027, >2]
	**Hypnotic suggestion**	**35.49**	**4.21**	**1.19**	**< .001**	**6.73 x 10** ^**6**^	[<0.001, >2]
Sham	**Lackner illusion**	**10.58**	**4.21**	**0.36**	**.026**	**2.68**	[0.105, 0.578]
	**Hypnotic suggestion**	**32.12**	**4.21**	**1.08**	**< .001**	**1.55 x 10** ^**6**^	[<0.001, >2]
Lackner illusion	**Hypnotic suggestion**	**21.54**	**4.21**	**0.72**	**< .001**	**1426**	[<0.001, >2]

Notes

^a^ = means in mm

^b^ = Holm-Bonferroni corrected

Significant effects are in bold. RR = robustness region. Prior size for reported Bs: √2/2

**Table 3:** Post-hoc comparison of the effect of conditions on response in the line reaching task (*N* = 50).

Line reaching task		Mean difference ^a^	*SE*	Cohen’s *d*	*p* ^b^	*B*_10_	*RR*
Baseline	Sham	2.34	4.72	0.07	.68	0.26	[0.52, >2]
	Lackner illusion	6.86	4.72	0.21	.45	1.94	-
	**Hypnotic suggestion**	**32.08**	**4.72**	**0.96**	**< .001**	**3715**	[<0.001, >2]
Sham	Lackner illusion	4.52	4.72	0.14	.68	0.88	-
	**Hypnotic suggestion**	**29.74**	**4.72**	**0.89**	**< .001**	**790**	[<0.001, >2]
Lackner illusion	**Hypnotic suggestion**	**25.23**	**4.72**	**0.76**	**< .001**	**266**	[0.002, >2]

Notes

^a^ = means in mm

^b^ = Holm-Bonferroni corrected. Significant effects are in bold. RR = robustness region. Prior size for reported Bs: √2/2

Our results with the Lackner illusion procedure are in the same range as those of previous experiments [16], or on a different body part such as the arm [15] and nose [13]. For example, in ref. [16] the typical elongation for the subgroup (10 of 30 participants) feeling their finger elongated was about one phalanx (about 2 cm), ref. [15] reported a mean elongation (for the responsive subgroup) of 51.5 mm and 33.3 mm in two experiments, and in ref. [13] the mean elongation for participants reporting an elongation was 44 mm. For the 13 participants clearly responding to the illusion (perceptual elongation greater than 20 mm) the mean elongation was 55 mm. Hence, it appears that the Lackner illusion manipulation produced results similar to previous studies. Nevertheless, we did not observe robust differences relative to the sham condition in the line reaching task.

We next sought to assess the potential role of control variables: age, gender, condition order, BMI, hypnotic suggestibility (WSGC), social desirability (self-deceptive enhancement and image management), and familiarity with hypnosis. We did so by including each variable in turn in the repeated measure ANOVA model as a covariate. As presented in Table 4, only hypnotic suggestibility had a significant effect, and only on responses in the perceptual task. Nonetheless, including this covariate in the model did not notably change the foregoing condition effects. All of the covariates yielded Bayesian evidence in favour of the null except age (finger length perception task), gender, familiarity with hypnosis, image management and condition order (line reaching task), for which the evidence was insensitive in discriminating between the null and alternative hypotheses.

**Table 4:** Covariate effects in repeated-measures ANOVAs examining condition effects on responses in the finger length perception task and line reaching task (*N* = 50).

	Finger length perception task				Line reaching task			
Covariate	*F*	*p*	*η* ^*2*^	*B*_*10*_^*a*^	*F*	*p*	*η* ^*2*^	*B*_*10*_^*a*^
Age	3.53	.066	.069	0.88	0.02	.896	< .01	0.27
Gender	0.74	.394	.015	0.30	1.09	.301	.022	0.44
BMI	0.56	.457	.012	0.29	0.43	.514	< .01	0.32
Hypnotic suggestibility	**8.91**	**.004**	**.157**	**7.35**	0.25	.622	< .01	0.31
Familiarity with hypnosis	0.48	.491	< .01	0.27	0.99	.325	.020	0.41
Self-deceptive enhancement	0.84	.365	.017	0.30	0.03	.855	< .01	0.28
Image management	0.08	.784	< .01	0.24	1.70	.198	.034	0.55
Condition order	0.14	.708	< .01	0.24	3.91	.054	.075	1.31

Notes. Covariates were introduced separately in each model; ^a^ = comparison of models including the condition and the covariate to the model only including the condition. Significant effects are in bold.

### Correlations

**Fig 4:**
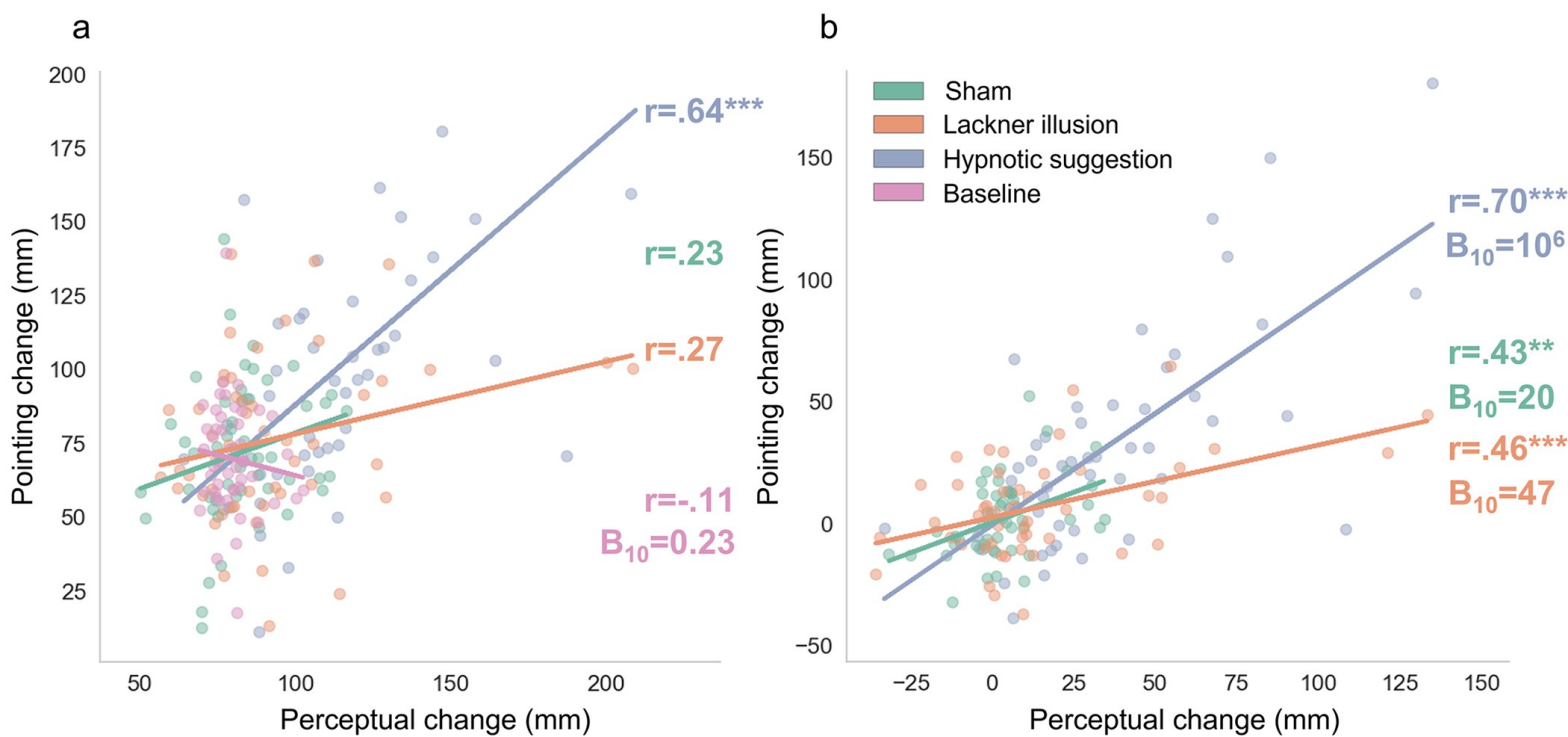
Scatterplots depicting the association between pointing effects and perceptual effects in the sham, Lackner illusion, hypnotic suggestion conditions and at baseline. Lines denote least squares regression slopes. (a) raw data, (b) baseline corrected data. *B*_10_ is the Bayes factor for the alternate hypothesis (correlation between variables). ** *p* < .01, *** *p* < .001.

In our next series of analyses, we assessed the correlations between responses in the two tasks in the different conditions. As can be seen in Fig 4, the perceptual and pointing effects were positively and significantly correlated in all conditions. This association was strong in the hypnotic suggestion condition but moderate in the Lackner illusion and sham conditions (with corresponding Bayesian evidence in favour of a strong positive association in all conditions). These results suggest a moderate to strong association between effects in the two tasks.

Finally, we examined whether the observed effects correlated with two measures of social desirability (SDE and IM) (Table 5). These effects were uniformly weak (*r*s < .19) and in yielded Bayesian evidence in favour of the null hypothesis in all cases except for the effect of hypnotic suggestion on pointing effects for SDE, which was insensitive. Cumulatively, these data strongly suggest the observed effects are unrelated to social desirability.

**Table 5:** Pearson correlation coefficients (with p-values and Bayes factors) between social desirability and responses in both tasks for each condition relative to baseline (*N* = 50).

		Sham perceptual effect	Sham pointing effect	Lackner illusion perceptual effect	Lackner illusion pointing effect	Hypnotic suggestion perceptual effect	Hypnotic suggestion pointing effect
Self-deceptive enhancement (SDE)	*r*	-.03	.11	.14	.12	-.01	.18
	*95% CI*	[-.29,.23]	[-.17,.37]	[-.14,.37]	[-.16,.37]	[-.27,.25]	[-.10,.43]
	*p*	.83	.46	.34	.42	.96	.20
	*B*_10_	0.18	0.23	0.27	0.24	0.18	0.39
	*RR*	[0.316, >2]	[0.506, >2]	[0.694, >2]	[0.552, >2]	[0.304, >2]	[1.315, >2]
Image management (IM)	*r*	.00	.08	.04	.01	-.04	.11
	*95% CI*	[-.26,.26]	[-.20,.35]	[-.23,.29]	[-.27,.28]	[-.29,.23]	[-.17,.37]
	*p*	.98	.57	.80	.97	.80	.46
	*B*_10_	0.18	0.21	0.18	0.18	0.18	0.23
	*RR*	[0.304, >2]	[0.413, >2]	[0.321, >2]	[0.303, >2]	[0.321, >2]	[0.506, >2]

RR = robustness region. Bs and RR are derived from a stretched beta distribution prior with width 1.0

## Discussion

This study compared the effects of hypnotic suggestion and the Lackner illusion manipulation on the perceived size of participants’ index finger (body image) and on the way they act with it (body schema). Body metrics comprise relatively low-level properties, which one might have expected to be immune to cognitive penetration. However, we found that not only did hypnotic suggestion induce illusory finger elongation at both visuospatial and sensorimotor levels, but also that these effects were significantly stronger than the corresponding magnitude of the sensory-driven Lackner illusion [13, 15, 16]. Furthermore, we report a pervasive correlation between perceptual and motor responses across all conditions, suggesting that these measures of body image and body schema are moderately to strongly interrelated. These results demonstrate that hypnotic suggestion is an effective technique for transient modulations of body image and body schema.

### Effects on body image and body schema

Although the mechanisms underlying the effects of hypnotic suggestion are still debated, it is widely accepted that they involve top-down regulation [22]. This implies that when relevant sensory evidence was minimized (participants were blindfolded and had no tactile inputs), body image was primarily shaped by high-level information originating from the verbal suggestion even though it was incongruent with prior knowledge about the finger size. By contrast, in the Lackner illusion condition there was no systematic expectation and misleading sensory signals induced by vibrations were present. In this condition, the contribution of prior knowledge was plausibly more predominant for most participants, as reflected in a weaker effect of this procedure relative to the hypnotic suggestion. It can indeed be safer for a cognitive system to rely more on the learning history of the organism than on noisy, and sometimes contradictory, sensory inputs. This finding is in line with other studies showing that participants’ perception is shaped more strongly by prior information in contexts of high uncertainty [76]. Accordingly, we interpret the condition difference as a tendency to favour data derived from high level representations (hypnotic suggestion) compared to sensory data (Lackner illusion) for updating body metrics.

The hypnotic suggestion also had a significant effect on body schema. This effect seemed tightly linked to the effect on body image as the two measurements were strongly correlated. Although the Lackner illusion modified body image, we did not find that it significantly modified body schema. It could be that we failed to observe a significant Lackner illusion effect on body schema because the effect was too noisy, too weak, or it could be that this illusion only influenced body image. The Bayesian evidence in favour of this effect was insensitive and thus we are unable to determine which of these outcomes led to the current results. Regardless, previous research has observed a dissociation showing that action can be immune to bodily illusions in some circumstances [14, 55, 56, 77] (but not in others e.g., [78]). Dissociations like this motivate the conceptual distinction between the two bodily representations [52].

Dissociations, however, are rare. Their scarcity, even in the clinical literature, can be explained by the fact that these representations normally interact and shape each other [54, 57, 58]. Such interaction can account for the correlation between perceptual and pointing effects in all conditions. In other words, regardless of the condition, variations in body image tend to be followed by corresponding changes in body schema.

Our data further revealed a positive correlation between perceptual effects in the hypnotic suggestion and Lackner illusion conditions, as we observed in another experiment [79]. This might reflect a sensitivity to alterations of bodily experiences towards bottom-up and top-down influences. Yet, detailed theoretical accounts integrating these influences on body representations are lacking.

### Theoretical accounts of the effect of hypnotic suggestion

The strong effect of the hypnotic suggestion on body schema compared to the Lackner illusion, can be interpreted at least in two different ways. Since the hypnotic suggestion explicitly revealed the intended purpose of the manipulation, it is possible that participants enacted what they interpreted to be the “correct” hypnotic behaviour. This interpretation aligns with some sociocognitive accounts of hypnosis [26, 80] that conceptualize hypnotic behaviour and experience as a culturally devised role and an active process. According to this account, the hypnotic suggestion did not *directly* alter body representations; rather, participants responded according to their expectations. This interpretation nicely explains the strong correlation between effects in the finger length perception and in the line reaching tasks. Indeed, if participants retained a normal body schema, they might index their pointing on their perceptual effect–i.e., the bias in the line reaching task was *intentionally* planned and enacted by the participant. According to this interpretation, participants imagined having a longer finger without critical appraisal, reported it in the perceptual task, and then intentionally undershot the targets in the line reaching task (with unbiased body schema). In other words, the perceptual effect could be interpreted as reporting imagery, and the pointing effect as adjusting the pointing motion to match the make-believe finger. In support of this interpretation, some highly suggestible individuals use active goal-directed strategies to bring about the suggested effect [81].

According to the predictive processing framework, representations are updated on the basis of their prior probability distribution and new available evidence [82–85]. In our case, the size of the index finger is usually inferred from prior knowledge stored in memory, and (new) sensory evidence. In the Lackner illusion condition, body image is computed based on incorrectly integrating information from illusory proprioceptive signals (arm extension caused by vibrations) and touch. These sensory signals are inconsistent with a normal finger length prior, or in other terms, the probability of having a normal sized finger given the sensory evidence is low. Hence, the corresponding finger representation shifts for some participants to an elongated finger on the basis of (misleading) sensory evidence. In contrast to the Lackner illusion manipulation, in the case of the hypnotic suggestion, no relevant sensory evidence is present; rather, the normal finger prior representation retrieved from memory and the elongated finger expectation derived from the suggestion compete based on their relative probabilities. This process may explain why participants responded on a continuum rather than according to a bimodal distribution. In turn, body image is shaped by the weighted average of the two priors with the weights corresponding to the precision of each (precision-weighting). When the suggested expectancies are unlikely for a participant, as might be the case for a difficult suggestion such as visual hallucination, the memory prior exerts greater influence. This yields a small effect and *vice versa*, if the more precise suggestion prior is heavily weighted. More broadly, beyond the setting of this experiment, the suggested prior could be supported by imagery and selective attention to seemingly confirming evidence while downplaying disconfirming evidence, though the relationship of these variable with response to suggestion is probably complex and controversial (on imagery e.g., ref. [79, 86]; on attention e.g., ref. [87–90]).

Further research is needed to stringently test the predictions of this account. Nonetheless, differential predictions can be derived from sociocognitive and predictive processing accounts in the context of the modulation of body representation through hypnotic suggestion. In particular, in cases wherein the hypnotic suggestion is incongruent with the close coupling of body image and body schema, sociocognitive accounts [26, 80] would predict that suggestion effects would dominate, whereas the predictive processing account would predict that the influence of suggestion would be weak. In particular, according to a sociocognitive account, behaviour can be adapted according to the suggestion irrespective of body image-schema coupling. By contrast, according to a predictive processing account, suggestions would have to compete with prior knowledge and thus suggestion effects would plausibly be diminished. For example, if participants were led to believe that the effect of experiencing one’s finger as longer was to point *past* the lines in the line reaching task (i.e., overshooting rather than undershooting), then sociocognitive accounts would predict a quasi-reversal of the association between perceptual and pointing effects in individuals who form this expectation. By contrast, a predictive processing account only predicts a small reduction of the effect of the hypnotic suggestion due to marginal compliance effects, but overall the same positive association between effects (see ref. [91] for a test).

Lastly, these two views discussed here do not overlap with awareness of intentions, in particular the sociocognitive view does not envision participants as lying or being deceitful. Metacognitive theories of hypnosis [92, 93] propose that intentional alteration of responses can occur outside of awareness. One version of this theory [92] maintains that intentions are preserved during hypnosis whereas high-order thoughts representing these first order intentions are inaccessible, thus resulting in a lack of awareness of one’s own intention (for supporting evidence, see [94, 95]). Regardless of awareness of intentions, which we did not measure in this study, our data suggest that the effect of hypnotic suggestion is not driven by compliance. If this effect were driven by compliance in response to the perceived requirements of the situation, social desirability would be expected to correlate with responses, at least in the hypnotic condition. Insofar as we observed a null correlation for this association, this suggests that our observed effects are unlikely to be driven by compliance, but we cannot rule out the possibility of broader effects related to demand characteristics.

### Limitations of the study

Despite the advances afforded by the present work, we acknowledge some limitations of this study. Firstly, we did not perform a non-hypnotic suggestion condition, nor an induction without suggestion. These would serve as potentially valuable controls for the hypnotic suggestion manipulation as they would have allowed us to better dissociate effects attributable to the induction and the suggestion. These conditions were not included because these questions were outside the scope of the present work and because we did not expect an induction to produce spontaneous finger elongation. Insofar as non-hypnotic direct verbal suggestibility is a reliable correlate of hypnotic suggestibility [96, 97], we expect that the present results would generalize to applications involving direct verbal suggestions without a formal induction procedure (as suggested by ref. [79]). Secondly, we did not measure participants’ expectancies for finger elongation. This would have allowed us to clarify to what extent the observed effects were mediated by this variable. We did not index response expectancies because doing so may provide participants with explicit indicators of how they are expected to respond. Relatedly, indexing response expectancies may introduce a confound wherein participants feel compelled to respond congruently with their expectations (consistency motivation) [98], thereby artificially biasing response patterns towards expectations. It should also be noted that different expectancies in different conditions does not necessarily mean that an effect (condition difference) is driven by expectancies. Nonetheless, future work would benefit from investigating the role of expectancies such as by formally manipulating them in different ways. Thirdly, we included all participants regardless of their responsiveness to the Lackner illusion procedure. This was intentional, as we wanted to assess the *general effect* of this illusion compared to the impact of hypnotic suggestion on perceived finger elongation. The effect of the illusion was significant on body image. Nonetheless, we observed relatively few participants who *unequivocally* responded to this illusion and as a result we could not perform analyses on this subgroup alone. A comparison of hypnotic suggestion and Lackner illusion manipulations in a homogeneous group displaying both effects would prove more informative regarding the interplay of bottom-up and top-down effects on body metrics, but the recruitment of such a population would be resource intensive. Fourth, the hypnotic suggestibility scale we used [66] displayed internal consistency that is marginally acceptable and it suffers from many problems that plague classical hypnotic suggestibility scales [99]. However, this scale is one of the most widely used scales in contemporary experimental hypnosis research [65, 100] and was deemed to the most optimal among French-adapted scales. Fifth, it would have been valuable to conduct systematic phenomenologically-oriented interviews with participants to elucidate the diversity of the experiences in the various conditions [81]. This research approach was not the primary target of our study, but it would be worthwhile to pursue in future research on this topic. Future work should similarly aim to compare and contrast the manipulations used here against other bottom-up manipulations, such as virtual reality (e.g., [7]). Sixth, in the present experiment the Lackner illusion was always preceded by the sham illusion condition. Although this can be seen as a limitation because of the lack of counterbalancing, a fixed order was implemented to avoid the possibility of participants detecting the sham illusion based on their previous experience of the Lackner illusion condition. Importantly, including the sham condition first is likely to provide a more conservative estimate of the experimental manipulations because it should not produce any perceptual distortion of the body (body image).

Finally, our conclusion that body image and body schema were altered by the Lackner illusion and hypnotic suggestion are based on inverse inference. Caution is advised in interpreting results of behavioural tests as indicators of latent unobservable variables [101]. Nonetheless, our claim that these behavioural tests are credible measures of body image and body schema rests on a wealth of prior data [14, 16, 59–61]. Furthermore, the link between the two body representations and behavioural measures is definitional. This link would be compromised in cases where participants misunderstood the task (e.g., reporting the preferred size of the finger instead of the perceived size) but no evidence points towards such a conclusion. We are confident that the association between our behavioural indicators and body metrics is sound but provisional, and it will depend on future development of the field.

### Clinical significance

The results presented here pave the way to future clinical research involving the use of hypnotic suggestion to modulate body representation. Bodily awareness is believed to be disturbed in eating disorders, such as anorexia nervosa [10, 11, 59, 60], and body image flexibility seems to be a valuable predictor of several psychopathological conditions [102, 103]. Hypnosis has demonstrated efficacy as an adjunct to traditional treatments [104–107], in particular in the treatment of psychosomatic disorders [108–111]. Hypnotic suggestibility is also known to be elevated in conditions involving aberrant body representation, such as functional paralysis [112, 113]. However, greater understanding of hypnotic suggestion pathways to modulate bodily awareness could delineate more clearly the areas where hypnosis could be clinically efficacious and where its use should be avoided. In particular, future research is necessary to elucidate the roles of hypnotic suggestibility and body representation flexibility as possible risk or protective factors in psychopathologies involving body awareness.

## Conclusion

Our objective was to compare sensory and cognitive contributions to bodily awareness. We showed a strong effect of hypnotic suggestion on body metrics at both sensorimotor and visuospatial levels. This effect was larger than the Lackner illusion manipulation on both levels. Bottom-up (Lackner illusion) and top-down (hypnotic suggestion) effects were correlated, suggesting that they both partly index a latent sensitivity of body metrics to modulation. We compared and contrasted two alternative accounts of these effects and outline ways by which they can be discriminated in future research.
