# Supplementary material for: Hypnotic suggestion versus sensory modulation of bodily awareness
Source: PLoS One. 2023 Sep 12;18(9):e0291493. doi: 10.1371/journal.pone.0291493 (PMC10497174; doi:10.1371/journal.pone.0291493)
Supplement: Attachment — Submitted filename: Response to Reviewers.pdf [file pone.0291493.s001.pdf]

Response to Reviewers

We thank the Editor and the Reviewers for their comments. Our responses (in blue) are below, and we have also highlighted corresponding changes in the text.

Journal Requirements:

When submitting your revision, we need you to address these additional requirements.

1. Please ensure that your manuscript meets PLOS ONE's style requirements, including those for file naming. The PLOS ONE style templates can be found at https://journals.plos.org/plosone/s/file?id=wjVg/PLOSOne_formatting_sample_main_body.pdf and https://journals.plos.org/plosone/s/file?id=ba62/PLOSOne_formatting_sample_title_authors_affiliations.pdf

The style of the manuscript has been updated accordingly.

2. Please provide additional details regarding ethical approval in the body of your manuscript. In the Methods section, please ensure that you have specified the name of the IRB/ethics committee that approved your study.

The approval for human experiment in the acknowledgement section has been copied to the material and methods section under the participants subsection (pp. 7):

“The whole experiment was approved by the ethics committee of Paris Descartes under IRB n°00012019-19 in agreement with the Declaration of Helsinki (2008). Informed written consent was obtained from each participant before the beginning of the study.”

3. Please provide additional details regarding participant consent. In the Methods section, please ensure that you have specified (1) whether consent was informed and (2) what type you obtained (for instance, written or verbal). If your study included minors, state whether you obtained consent from parents or guardians. If the need for consent was waived by the ethics committee, please include this information.

The approval for human experiment in the acknowledgement section has been copied to the material and methods section under the participants subsection (pp. 7):

“The whole experiment was approved by the ethics committee of Paris Descartes under IRB n°00012019-19 in agreement with the Declaration of Helsinki (2008). Informed written consent was obtained from each participant before the beginning of the study.”

4. Please change "female” or "male" to "woman” or "man" as appropriate, when used as a noun (see for instance https://apastyle.apa.org/style-grammar-guidelines/bias-free-language/gender).

The modification has been made (pp. 6).

5. We note that the grant information you provided in the ‘Funding Information’ and ‘Financial Disclosure’ sections do not match.

When you resubmit, please ensure that you provide the correct grant numbers for the awards you received for your study in the ‘Funding Information’ section.

We can now confirm that they are aligned.

6. Thank you for stating the following in the Competing Interests section:

"C.A. has read the journal's policy and the authors of this manuscript have the following competing interests: scholarship from ARCHE formation. D.B.T. and F.V. have declared that no competing interests exist."

We note that one or more of the authors have an affiliation to the commercial funders of this research study: ARCHE formation

(1) Please provide an amended Funding Statement declaring this commercial affiliation, as well as a statement regarding the Role of Funders in your study. If the funding organization did not play a role in the study design, data collection and analysis, decision to publish, or preparation of the manuscript and only provided financial support in the form of authors' salaries and/or research materials, please review your statements relating to the author contributions, and ensure you have specifically and accurately indicated the role(s) that these authors had in your study. You can update author roles in the Author Contributions section of the online submission form.

Please also include the following statement within your amended Funding Statement. “The funder provided support in the form of salaries for authors [insert relevant initials], but did not have any additional role in the study design, data collection and analysis, decision to publish, or preparation of the manuscript. The specific roles of these authors are articulated in the ‘author contributions’ section.” If your commercial affiliation did play a role in your study, please state and explain this role within your updated Funding Statement.

(2) Please also provide an updated Competing Interests Statement declaring this commercial affiliation along with any other relevant declarations relating to employment, consultancy, patents, products in development, or marketed products, etc.

Within your Competing Interests Statement, please confirm that this commercial affiliation does not alter your adherence to all PLOS ONE policies on sharing data and materials by including the following statement: ""This does not alter our adherence to PLOS ONE policies on sharing data and materials.” (as detailed online in our guide for authors http://journals.plos.org/plosone/s/competing-interests). If this adherence statement is not accurate and there are restrictions on sharing of data and/or materials, please state these. Please note that we cannot proceed with consideration of your article until this information has been declared.

Please include both an updated Funding Statement and Competing Interests Statement in your cover letter. We will change the online submission form on your behalf.

This has already been declared in the original financial disclosure statement “This research was supported by ARCHE formation (scholarship of C.A. and experimental expenses, no grant number, https://www.arche-hypnose.com/), FrontCog (ANR-17-EURE-0017 FrontCog, https://www.psl.eu/en/front-cog) and PSL (ANR-10-IDEX-0001-02 PSL, https://psl.eu/en). The funders had no role in the study design, data collection and analysis, decision to publish,

or preparation of the manuscript." and the original competing interests statement "C.A. has read the journal's policy and the authors of this manuscript have the following competing interests: scholarship from ARCHE formation. D.B.T. and F.V. have declared that no competing interests exist."

7. Please include your full ethics statement in the 'Methods' section of your manuscript file. In your statement, please include the full name of the IRB or ethics committee who approved or waived your study, as well as whether or not you obtained informed written or verbal consent. If consent was waived for your study, please include this information in your statement as well.

The approval for human experiment in the acknowledgement section has been copied to the material and methods section under the participants subsection. (pp. 7).

"The whole experiment was approved by the ethics committee of Paris Descartes under IRB n°00012019-19 in agreement with the Declaration of Helsinki (2008). Informed written consent was obtained from each participant before the beginning of the study."

Reviewer #1:

The study investigated how cognitive (hypnotic suggestion) versus sensory manipulations influence bodily awareness. The experiment appears to be well-designed, the data visualization is clear, and the data itself have been made open in a public repository, which are all solid strengths of the study. The statistical analyses are described in sufficient detail, and the conclusions are presented in an appropriate fashion and are supported by the data. There are a few minor issues that should be addressed before proceeding with publication of this study, which I pointed out below, hoping they are relevant for the authors to improve their manuscript.

Page 4, line 26: The peer-reviewed version of the paper can be found here: Lush, P., Botan, V., Scott, R. B., Seth, A. K., Ward, J., & Dienes, Z. (2020). Trait phenomenological control predicts experience of mirror synaesthesia and the rubber hand illusion. Nature communications, 11(1), 1-10.

We have corrected this error – thanks.

Page 5, line 6: Given the context of the subsequent sentence, I believe the authors wanted to refer to the following paper: Lush, P., Botan, V., Scott, R. B., Seth, A. K., Ward, J., & Dienes, Z. (2020). Trait phenomenological control predicts experience of mirror synaesthesia and the rubber hand illusion. Nature communications, 11(1), 1-10. (Although Lush, 2020 is also an example of a study on RHI and hypnotic suggestibility)

We substituted the suggested reference, this is indeed more relevant.

Page 5, line 7: The peer-reviewed version of the paper can be found here: Ehrsson, H. H., Fotopoulou, A., Radziun, D., Longo, M. R., & Tsakiris, M. (2022). No specific relationship between hypnotic suggestibility and the rubber hand illusion. Nature communications, 13(1), 1-3.
For a further insight in the investigation of the potential influence of hypnotic suggestibility on

the RHI, the authors could consider discussing the following article in the Introduction and/or Discussion: Slater, M., & Ehrsson, H. H. (2022). Multisensory integration dominates hypnotisability and expectations in the rubber hand illusion. Frontiers in Human Neuroscience, 317.

We corrected the version error and added the suggested article. However, given the comments of reviewer 2 and the fact that this discussion is not central to the paper, we shortened the discussion around the RHI (pp.4) "Furthermore, it has been recently debated to what extent the RHI could be explained by demand characteristics [44,48,49], that is, by participants' explicit compliance, or unconscious changes in behaviour and experience in accordance with implicit or explicit experimental aims [50]"

Reviewer #2:

This manuscript presents evidence for an influence of a hypnotic suggestion and a vibrating stimulus applied to the arm for two measures of perceived finger length. I find the initial concept very interesting (my colleagues and I have also been testing direct suggestion for other body illusions recently) and it is great to see the use of a sham control (though claims regarding this would be strengthened by a test of expectancies for the two conditions in a separate sample). The manuscript is appropriately structured and the standard of English is good. However, major conclusions (in particular regarding body schema and image), are not sufficiently supported by the data and there are a number of major problems with the presented analyses and their interpretation (e.g., non-significant p values have been used to support the null). In addition, insufficient detail has been provided for the mixed linear models. The authors may be able to address these issues in a revision, and I have provided some detailed consideration of each which I hope may be useful. Because this manuscript closely relates to much of our recent research, I have referenced papers by my colleagues and I and sign my review in the interests of transparency.

<u>Null results and Bayes factors</u>

Although there are Bayesian tests of some null results there are also many instances in which a non-significant p value has been erroneously interpreted as evidence of no difference.

E.g., "although the model did not improve if we allowed for an interaction between hypnotic suggestibility and condition, $\chi^2(2,N=50)=3.28$, p=.19." Please add Bayes factors in each case, or revise the text to clarify that no conclusions have been drawn from these non-significant p values. Also revise discussion text relating to assertion of the null based on non-significant p values (e.g., p.21 "Somewhat unexpectedly, familiarity with hypnosis did not significantly correlate with hypnotic suggestibility. Some correlation was…"). I have given only a couple of examples, but there are other similar examples throughout. Please provide the model for H1 for the Bayes factors. They are uninterpretable without this information. If a default model was used, it's relevance to the predicted effect size should be argued. If it is not relevant, a relevant prediction should be used. I find Dienes (2019) helpful for specifying predicted raw effects.

We agree with the reviewer that a non-significant p-value should not be interpreted as evidence supporting the null hypothesis. It is also true that using Bayes Factor in several instances rather than p-values allows for a stronger conclusions, and we thank the reviewer for this suggestion. On the other hand, we do think it is not inaccurate to state "did not significantly correlate" – please note that this is not a not a null claim (e.g., "did not correlate") – only a claim that a significant effect was not observed. We do acknowledge that in a few cases we incorrectly omitted the word "significant" and we have made sure to include that as appropriate throughout. This is especially appropriate when we're not interested in the null per se but only that a covariate did not significantly confound our results. We have now added Bayes factors for all analyses (see Table 1, Table 2, Table 3, Table 4 and Fig 4). We have now clearly stated that we are using default Bayes factors throughout.

Other analyses

I found the analyses very difficult to follow. A more detailed explanation of why particular analytical decisions were taken would be helpful. I did not find it possible to adequately assess the results in their present form. There seems to be a mixture of approaches. For example, why are there correlations in addition to the mixed linear models? I recommend sticking to one approach throughout. For example, (1) use ANOVAs and correlations on participant means, or (2) use LMMs for condition effects and corrrelations, and assess significance with t-tests on the coefficients, or (3) use LMMs for condition effects and correlations and assess significance using likelihood ratio tests In addition please: Report the LMM in R notation, including how each condition was coded (e.g. sham = 1, not sham = 0)". Specify what the betas are in table 1. Are they contrasts? If so, please ensure the LMM specification (given in bold) matches the parameters in the table For each statistical test, please make explicit what the DV is. For example, what are the t-tests in BFs in paragraph 2 of section 3.2 calculated on? If they were calculated on betas, please explain that the LMMs were refitted separately for each level of Condition Order

We thank the reviewer for these suggestions, which have helped us to clarify the results. We opted for the first suggested method as readers might be most familiar with it. The new result section is in line with what was done before. It should be noted that it did not change the main results and conclusions.

Conclusions regarding body image and schema

Measures are conflated with the constructs they are posited to reflect; the names of two tasks are treated as interchangeable with "body schema" and "body image", leading to overstatements in the central interpretation of results, e.g., p. 18 "These results demonstrate that the hypnotic suggestion is an effective technique for transient modulations of body image and body schema."; p.19 "The hypnotic suggestion also had a significant effect on body schema.". The presented arguments do not provide adequate support for claims that, for example, hypnotic suggestion modifies body schema. This issue could be addressed by referring to the measures directly rather than to the posited constructs, except when attempting to justify the link between the two through argument (e.g., p.23-25) and by removing, toning down or qualifying conclusions regarding body schema and image throughout.

Claims that response to an imaginative suggestion for a particular experience necessarily involves particular processes posited to underly similar experiences in other contexts are unsafe. As acknowledged in the discussion (p. 23 & 24), response to imaginative suggestion can be both interpretative and imaginative. Few would argue that the examples given in the introduction of hypnotically induced amnesia and blindness necessarily occur by the same mechanisms as other cases of amnesia and blindness. That an imaginative suggestion for an experience of finger lengthening involves changes in body image or schema seems to me little different than the claim that a moving hands together suggestion (e.g., in the WSGC) reveals an innate magnetic sense. I think the approach taken opens rather a large can of worms - hypnosis has a long history of such claims. While in some cases these are too implausible to be taken seriously (past life regression) in others they have been taken seriously and caused considerable problems (e.g., 'recovered' memories).

The following justification for reverse inference is given on p.25: "Caution is advised in interpreting results of behavioural tests as indicators of latent unobservable variables (Bach et al., 2018). Nonetheless, our claim that these behavioural tests are credible proxies of body image and body schema rests on current practices of the community(...) It follows that this inverse inference problem is rather a trivial limitation, meaning that the association between our behavioural indicators and body metrics is provisional and will depend on future development of the field." If my interpretation of this argument is correct, I do not find it convincing. A mistake being common does not indicate that it is trivial (I would argue the contrary). I don't believe the experimental design can test "whether hypnotic suggestion can effectively modulate body image and/or body schema". Response to these pointing and perceptual tasks might be taken to indicate relevant experience, but that such experience involves changes in "body image" or "body schema" is just one possibility, just as it is only one possibility among many that a subject's regressed memories of life as Cleopatra are veridical. There are very good reasons to tread carefully here – the range of potential 'discoveries' which might arise from the application of reverse inference to response to imaginative suggestion is potentially vast.

We appreciate the reviewer's comments on the interpretation of behavioural measurement in our study. This is an important issue, which is actually widely spread in cognitive neuroscience: how to go from behaviours to what's inside the back box? In the case of bodily processing, since Head and Holmes (1911), there has been a clinical tradition to take performance in perceptual or motor tasks as evidence in favour of a deficit of corresponding bodily representations (e.g. Paillard, 1999; Sirigu et al., 1991; Schwoebel and Coslett, 2005). It has further been found that performance in perceptual and motor tasks can be selectively modulated (e.g., Kammers et al., 2009; Gadsby, 2017). Finally, they correspond to distinct neural networks (Berlucchi and Aglioti, 2010; Naito et al., 2016; Ho and Lenggenhager, 2021). There may be other interpretations for all these results, but there is so far no evidence showing that these tasks do *not* measure two distinct types of bodily representations. Consequently, we believe that the body schema/body image hypothesis remains empirically and conceptually grounded. We have discussed extensively this hypothesis in other works (Vignemont, 2011; Vignemont, 2018; Vignemont et al., 2021) and we do not believe that our arguments should be repeated in this paper.

We should also note that researchers routinely operationalize a term with a measure and then reference the term even though others might disagree (e.g., operationalizing phenomenological control as suggestibility and then interpreting the latter as the former). We also don't think the analogies with past life regression or animal magnetism are relevant here just as we would never draw analogies between phenomenological control and animal magnetism.

p.5 “Furthermore, demand characteristics – namely participant’s explicit compliance, or unconscious changes in behaviour and experience in accordance, with implicit or explicit experimental aims (Orne, 1962) – were recently debated as a potential contributing factor to the RHI (Lush, 2020). Effects turned out to be weak and unsignificant when relevant controls were applied (Ehrsson et al., 2021).” p.19 “ (for the RHI, it was shown that with adequate control, the association was insignificant: Ehrsson et al., 2021).”

There is some confusion here. Lush (2020) showed that expectancies differ for the asynchronous control and synchronous illusion conditions of RHI studies. (see also replications in Reader 2022; Lush, Seth & Dienes, 2022). Asynchrony is not a valid control in the RHI, simply because control conditions must be matched for all important factors, and expectancies are clearly an important factor when controlling for demand characteristic effects. Ehrsson et al (2021) did not address the results of Lush (2020), but the results of Lush et al, (2020) which reported relationships between synchronous condition rubber hand illusion measures and hypnotisability, and also evidence for no relationship between illusion agreement scores and hypnotisability when ‘controlled’ by the asynchronous condition. Ehrsson et al reproduced this latter null result The interpretation of this null result rests on the validity of the control condition. Ehrsson et al assert that the control is valid, without evidence or argument. Similarly, in the manuscript, the control condition is described as ‘relevant’ and ‘adequate’, but no argument or reference for why it should be considered so has been provided. Please either provide an argument as to why this control procedure which is confounded by expectancies should be considered a valid control for demand characteristic effects or remove or revise this assertion. Rather than repeat our interpretation of this null result and related arguments here, I refer the authors to the arguments presented in our commentary published alongside Ehrsson et al (2021): Lush & Seth (2021) and in the original paper (Lush et al, 2020). In anticipation of a possible response, it is worth noting that a recent argument about the manipulation of expectancies in the original paper (deVignemont, 2022) is based on a misreading of our study design. It should also be noted that, even if the RHI control condition were valid, it would not provide good support for the claim that demand characteristics are not involved in the effects presented in this paper (in order to attribute the effects to changes in body schema etc).

We no longer think this debate is especially relevant to the current work and we have shortened or omitted discussion of these points. It should be noted that different expectancies in different conditions does not necessarily mean that an effect (condition difference) is driven by expectancies. For example, if we ask a group of athletes and a group of non-athletes their expectancies concerning their performance on a sportive task, we will likely have a group with high expectations and high scores and a group with low expectations and low scores. It would be a mistake to conclude that participants achieved higher scores because of their expectancies. We think this is an important point that has been lost in this discussion and we briefly highlight this in the “limitations of the study” section. Irrespective of control conditions in the RHI, we maintain that our sham control is adequate because it mimics all the aspects of the Lackner illusion procedure to the participant. Importantly, it doesn’t reliably elicit the illusion. We think studying expectancies with this paradigm is a useful suggestion and we have now made a recommendation regarding this in the Discussion.

P.20, section 4.2. The simple alternative theory that differences in effects between illusion and suggestion conditions may arise from differing demand characteristics is treated inconsistently in this section. While it is considered here as a possible explanation for order effects (as also may occur in the RHI, see Lush, 2021), it may also explain other results and should be consistently applied to conclusions. For example, the relatively clear demand characteristics in the hypnotic suggestion condition may explain the correlation between measures in the suggestion condition in contrast with the illusion condition (though please ensure all claims of null results are backed by analyses other than non-significant p values - see below).

An argument about why we think that the increased correlation is not a result of demand characteristics has been added to the manuscript (pp.22-23) "*First, if the effect of the hypnotic suggestion were caused by compliance in response to demand characteristics, social desirability would be expected to correlate to some extent with effects in the hypnotic condition, which is against the evidence. Second, this interpretation predicts that pointing effect follows perceptual effect. Indeed, in this view participants might shift their perceptual reports in response to demand characteristic but correctly follow instructions in the pointing task. However, the opposite does not hold, as participants going as far as shifting their pointing in line with the suggestion should report at least similar elongation in the perceptual task. One consequence is that the distribution of differences between perceptual and pointing effects should be shifted towards positive values, with negative values only corresponding to pointing inaccuracies. However, the data suggest that it is not the case, with the discrepancy between perceptual and pointing effect being reasonably close to a gaussian distribution centred on zero.*"

A further related issue - the indirect measures bear surface similarity to proprioceptive drift in the RHI (which is confounded by demand characteristics; Lush, Seth & Dienes, 2021) and may have similar problems.

As noted above, a demonstration of different expectancies does not mean something is confounded only that it might be confounded. To convincingly demonstrate this, it will be important to modulate expectancies. Sometimes expectancy claims are overstated, and we don't want to make that mistake here. We have noted that further interrogation of features of the principal features of this illusion and procedure are warranted.

"However, in contrast with claims made by some of these authors, a correlation between hypnotic suggestibility and a bodily illusion does not necessarily entail that an illusion is driven by suggestion and expectations."

Please clearly state which of the papers contain this claim. Without direct reference, it is a strawman (I don't recall seeing this argument in any of the cited papers, but I could well have missed it). As an aside, regarding the plausibility of suggestion effects in the rubber hand illusion, I think it is a mistake to consider this one illusion in isolation – similar relationships have been shown for mirror touch synaesthesia pain, ASMR and visually evoked auditory response. While other mechanisms may be in play in each case, that these relationships are due to imaginative suggestion effects arising from demand characteristics presents a simple, testable theory.

We fully agree that this is an interesting hypothesis that warrants further attention. However, strong claims along these lines have been made in multiple papers:

“First, as a way of more rigorously exploring phenomena which phenomenological control can surreptitiously bring about without subject nor experimenter necessarily realizing.” Lush et al. 2021 (https://psyarxiv.com/zhgeb/)

Correlations between these phenomena and suggestibility only show that they’re correlated not that suggestion can bring about these effects.

“If the effect is entirely produced by phenomenological control the slope should be steep and the intercept close to 0. … Indeed, the extent to which people experience vEAR is strongly related to their score on the Phenomenological Control Scale (with zero intercept […]”. (Dienes & Lush, 2022 “The role of phenomenological control in experience”).

Insofar as two different variables could correlate with a third variable and both effects could have zero intercepts, this doesn’t hold. Either way, note the use of “entirely produced” when referring to a correlation.

“That is, the sensations of ASMR may be largely constructed based on a belief of the situations that should bring them about." (Dienes & Lush, 2023).

Again, these are correlational data.

“Evidence that hypnotisability scores predict the most common RHI measures would be consistent with the theory that the RHI, as commonly reported, is at least partially driven by phenomenological control.” (Lush et al. 2020 pp.4)

“Our results show that RHI reports are, at least partially, likely to be driven by top-down phenomenological control in response to demand characteristics” (Lush & Seth, 2022 pp.1)

“Rubber hand illusion report is confounded by demand characteristics and may entirely reflect compliance, bias and implicit imaginative suggestion effects“

This is the title of the original paper.

"Mirror-touch synesthesia occurs when a person feels touch that they see someone else receive. Vicarious pain occurs when a person feels pain when they see someone else receiving noxious stimulation. Both effects depend steeply on hypnotizability, with small though not necessarily zero intercepts, when tested in unrelated contexts (Lush et al., 2020)."

Note the word "depend", which denotes a causal relationship.

P.22. “ In this framework, the hypnotic suggestions did not directly alter body representations; rather, participants changed their behaviour and experience according to their expectations...”

This paragraph is a good summary which matches my interpretation of the results. I don’t see anything in these data which is not consistent with this position. While it is only one

interpretation, that it can be applied to the data presented without issue highlights that the conclusions regarding body schema etc are an overstatement of the presented results.

Problems with this interpretation have been added to the manuscript (pp.22-23) "*First, if the effect of the hypnotic suggestion were caused by compliance in response to demand characteristics, social desirability would be expected to correlate to some extent with effects in the hypnotic condition, which is against the evidence. Second, this interpretation predicts that pointing effect follows perceptual effect. Indeed, in this view participants might shift their perceptual reports in response to demand characteristic but correctly follow instructions in the pointing task. However, the opposite does not hold, as participants going as far as shifting their pointing in line with the suggestion should report at least similar elongation in the perceptual task. One consequence is that the distribution of differences between perceptual and pointing effects should be shifted towards positive values, with negative values only corresponding to pointing inaccuracies. However, the data suggest that it is not the case, with the discrepancy between perceptual and pointing effect being reasonably close to a gaussian distribution centred on zero.*"

Other points

As far as I can tell, it seems that one of the two measures of the Lackner illusion employed here may say survive sham control. This, to me, was among the more interesting aspects of the present manuscript. I think it would be worth bringing out more.

We thank the reviewer for his interest in that part. The effect of the Lackner illusion on the perceived size of the finger is only a replication of previous studies (Lackner 1988, Burrack & Brugger 2005, Vignemont et al. 2005, Ehrsson et al. 2005) and therefore it is not very surprising. Since it is quite peripheral to our main question, we prefer not commenting more on that result.

P.8 "By comparing the reported finger size at rest to the actual finger size, we can derive an estimate of the validity of the measure." I think I get the intended meaning, but the use of "validity" here is inadvisable given the specific meanings of the term in psychological science.

It is true that validity here is a misnomer, and we changed it: "…*we can derive an estimate of the bias in the measurement*" (pp.8)

P.23 "supported by imagery and selective attention"

It might be worth noting evidence against trait differences in attention in response to imaginative suggestion here (see Martin & Pacherie, 2018, for example).

Thank you for the suggestion. It is true that there is no clear relationship between measures of attention and hypnotic suggestibility, and it deserves to be mentioned here. *"…the suggested prior could be supported by imagery and selective attention to seemingly confirming evidence while downplaying disconfirming evidence, though the relationship of these variable with response to suggestion is probably complex and debated (on imagery e.g., [96,97]; on attention e.g., [98–101]).*" (pp.23-24)

P.24 “We did not index response expectancies because doing so may provide participants with explicit indicators of how they are expected to respond”

These could be checked in a separate sample, e.g., to address (possibly) differing results between hypnosis and illusion conditions (I’m not suggesting you should do this now, btw).

Agreed, this might be an experiment we will do in the future.

Results are sometimes reported in cm and sometimes in mm. Better to choose one or the other throughout.

Indeed, this has been changed so that distances are in mm throughout, thank you for the suggestion.

Open data – the variables in the provided file are difficult to interpret without a key. For example, what do the ‘pinocchio’ columns refer to?

The OSF file has been updated to be interpreted more easily. Header names referred to older version of the manuscript where the “Lackner illusion” was referred to as “Pinocchio illusion” as it is sometimes used in the community.

Signed

P Lush

Dienes, Z. (2019). How do I know what my theory predicts?. Advances in Methods and Practices in Psychological Science, 2(4), 364-377.

Lush, P. (2021, July 19). Order effects in the rubber hand illusion. https://doi.org/10.31234/osf.io/amsrp

Lush, P., Seth, A. K., & Dienes, Z. (2021). Hypothesis awareness confounds asynchronous control conditions in indirect measures of the rubber hand illusion. Royal Society open science, 8(11), 210911.

Lush, P., & Seth, A. K. (2022). Reply to: no specific relationship between hypnotic suggestibility and the rubber hand illusion. Nature communications, 13(1), 1-3.

Reader, A. T. (2021). What do participants expect to experience in the rubber hand illusion? A conceptual replication of Lush (2020).
