# Supplementary material for: Hypnotic suggestion versus sensory modulation of bodily awareness
Source: PLoS One. 2023 Sep 12;18(9):e0291493. doi: 10.1371/journal.pone.0291493 (PMC10497174; doi:10.1371/journal.pone.0291493)
Supplement: Attachment — Submitted filename: Lackner RHI review.pdf [file pone.0291493.s002.pdf]

.

This manuscript presents data for a finger length perception task and a line reaching task in three conditions: sham control, Lackner illusion and a hypnotic suggestion for finger elongation which is comparable to a classic hypnosis scale item for an experience of the hands being drawn together by an imaginary magnetic force. There is evidence for a Lackner effect larger than the effect in the sham condition (though interpretation of this difference is complicated by a lack of evidence that a confounding variable is matched across conditions). The authors appeal to differences in the relationships between trait response to hypnotic imaginative suggestion (hypnotisability) and perception measure response between the hypnotic suggestion and Lackner conditions to support arguments about mechanism, but there is no evidence for or against a difference in these relationships. In my view, the difference between the Lackner illusion and hypnotic suggestion condition could simply reflect a difference in expectancies between the two conditions. The hypothesis-consistent demand characteristics seem to me likely to be clearer for the hypnotic suggestion than for the Lackner condition (because in the first case they are explicit and in the latter case implicit).

I did not review the results in detail in the first version of the manuscript. The results have now been extensively revised, and I will therefore focus this review the results and related claims in the discussion. Many of the claims in the discussion are based on invalid inferences, which I will detail below. I will focus on the perception measure because for the line-reaching measure there was no evidence for or against an effect in the Lackner condition, or evidence for a difference between sham control and Lackner condition.

I recommend major revision to remove invalid inferences and related discussion.

1) *Response : We agree with the reviewer that a non-significant p-value should not be interpreted as evidence supporting the null hypothesis. It is also true that using Bayes Factor in several instances rather than p-values allows for a stronger conclusions, and we thank the reviewer for this suggestion. On the other hand, we do think it is not inaccurate to state "did not significantly correlate" – please note that this is not a null claim (e.g., "did not correlate") – only a claim that a significant effect was not observed. We do acknowledge that in a few cases we incorrectly omitted the word "significant" and we have made sure to include that as appropriate throughout. This is especially appropriate when we're not interested in the null per se but only that a covariate did not significantly confound our results. We have now added Bayes factors for all analyses (see Table 1, Table 2, Table 3, Table 4 and Fig 4). We have now clearly stated that we are using default Bayes factors throughout.*

I thank the authors for removing some invalid claims based on non-significant p values (e.g., that a non-significant result is surprising). However, there are many such claims in the discussion section of the revised manuscript. For example, claims of differences for

significant and non-significant correlations between hypnotisability and either the Lackner illusion or hypnotic suggestion conditions**.** The only evidence for or against a relationship in these cases is a p value below .05 for a positive relationship between hypnotic suggestion response and hypnotisability.

p.19: "Weaker and non-significant correlations with hypnotic suggestibility were observed for perceptual effect in the Lackner illusion and sham condition."

p.19 "Our data do not exclude the possibility that hypnotic suggestibility accounts for variability in the Lackner illusion,although our sample size was not large enough to detect this association. Nevertheless, our results suggest that *if* this association were present, it would be rather weak."

p.19 "This sensitivity to alterations of bodily experience across bottom-up and top-down contexts is probably not reducible to hypnotic suggestibility since correlations with effects in the Lackner illusion condition were weak and unlikely to differ from zero."

*"*Relatedly, the specific effect of the Lackner illusion cannot be explained by top-down processing since it survived the inclusion of hypnotic suggestibility as a covariate in our model of perceptual effect."

Furthermore, to support claims of a difference between the correlations, a test of the difference is required. I calculated confidence intervals from the online data (the manuscript is lacking CIs and SEs). The (bootstapped) 95% CI for the Pearson coefficient between hypnotisability and Lackner illusion is [.02, .48] and for the relationship between hypnotisability and response to suggestion [.08, .51]. The CI for the hypnotisability/Lackner illusion coefficient contains the point value of the parameter estimate for the hypnotisability/suggestion coefficient. The data are simply too noisy to distinguish between these relationships. A larger sample would be required.

There is no statistical evidence for or against a line reaching/pointing effect in the Lackner condition to support the following claims:

p.20 "Although the Lackner illusion did modify body image, we found that it did not significantly modify body schema. This dissociation aligns with similar results showing that action can be immune to bodily illusions in some circumstances"

p.21 "Body image and body schema can be sometimes dissociated, thus motivating the conceptual distinction between the two bodily representations [51]. This seems to be confirmed by the dissociation between the effect of the Lackner illusion on body image, and the lack of effect on body schema."

19. Furthermore, the stimulation was apparently similar between the sham and the Lackner illusion procedure and the instructions were identical. The sham condition was thus able to provide a good control condition for confounding expectancies, as suggested by similar correlations with hypnotic suggestibility.

No justification for models of H1 has been included. This issue could be somewhat mitigated If robustness regions are reported (so that readers can see the range of models consistent with the inference). Bayes factors are only informative regarding the relative evidence for two theories. If the model of H1 is not reasonable, the Bayes factor is not useful, but the authors provide no argument as to why the chosen model of H1 is considered reasonable. There are earlier studies of the Lackner illusion which could inform the choice of model.

Models of H1 have only been provided for post-hoc comparisons, and not for other analyses (e.g., correlational analyses).

No criterion is provided for the interpretation of Bayes factors.

2) *Response : We appreciate the reviewer's comments on the interpretation of behavioural measurement in our study. This is an important issue, which is actually widely spread in cognitive neuroscience: how to go from behaviours to what's inside the back box? In the case of bodily processing, since Head and Holmes (1911), there has been a clinical tradition to take performance in perceptual or motor tasks as evidence in favour of a deficit of corresponding bodily representations (e.g. Paillard, 1999; Sirigu et al., 1991; Schwoebel and Coslett, 2005). It has further been found that performance in perceptual and motor tasks can be selectively modulated (e.g., Kammers et al., 2009; Gadsby, 2017). Finally, they correspond to distinct neural networks (Berlucchi and Aglioti, 2010; Naito et al., 2016; Ho and Lenggenhager, 2021). There may be other interpretations for all these results, but there is so far no evidence showing that these tasks do not measure two distinct types of bodily representations. Consequently, we believe that the body schema/body image hypothesis remains empirically and conceptually grounded. We have discussed extensively this hypothesis in other works (Vignemont, 2011; Vignemont, 2018; Vignemont et al., 2021) and we do not believe that our arguments should be repeated in this paper.*

I presume that in each of these other cases the experimental effects have been at least assumed to not be suggestion effects. That's not possible for a direct suggestion.

As a side note, I should clarify my comments on a body schema interpretation of the suggestion condition in my previous review. I was not saying you shouldn't be free to make

the claim that hypnotic suggestion changes body schema, but rather noting some responses I think this claim is likely to face from imaginative suggestion researchers.

3) *We should also note that researchers routinely operationalize a term with a measure and then reference the term even though others might disagree (e.g., operationalizing phenomenological control as suggestibility and then interpreting the latter as the former). We also don't think the analogies with past life regression or animal magnetism are relevant here just as we would never draw analogies between phenomenological control and animal magnetism.*

This paragraph is not directly relevant to my review of this manuscript, but note that "phenomenological control is not "operationalized as suggestibility"". It is a label we use to refer to hypnotic suggestibility (and response to implicit or direct suggestion in contexts other than hypnosis) with the aim of avoiding misunderstandings arising from other terms.

E.g., Dienes et al (2022) https://psycnet.apa.org/doi/10.1037/cns0000230: "Phenomenological control is the ability to control subjective experience so that a constructed counterfactual state of affairs appears real. It is not a new concept. We as a community have been calling it hypnotizability (in the context of hypnotic inductions) or suggestibility. But "hypnosis" refers to sleep, which is unrelated to phenomenological control (e.g. Banyai & Hilgard, 1976); and "suggestibility" implies being manipulated contrary to one's real intentions, a gullibility that is little related to hypnotic response (e.g. Coe, Kobayashi, & Howard, 1973; Moore, 1964). So in our laboratory, we have begun to refer to hypnotic suggestibility in a more general context as the capacity for phenomenological control and, correspondingly, hypnotic response as an example of phenomenological control."

I do not consider reference to mesmerism or hypnotic suggestion effects to be irrelevant or inappropriate when discussing an experimental investigation of response to hypnotic suggestion. Neither my colleagues nor I would be offended by comparisons between phenomenological control and mesmerism. Phenomenological control is an explanation of mesmerism. We often discuss mesmerism alongside phenomenological control.

4) *We no longer think this debate is especially relevant to the current work and we have shortened or omitted discussion of these points. It should be noted that different expectancies in different conditions does not necessarily mean that an effect (condition difference) is driven by expectancies…*

We do not anywhere claim that expectancy studies show an effect is necessarily attributable to demand characteristics. I stated our argument in my first review: "Asynchrony is not a valid control in the RHI, simply because control conditions must be matched for all

important factors, and expectancies are clearly an important factor when controlling for demand characteristic effects."

An experiment which employs controls for a known extraneous variable which is unmatched on that extraneous variable cannot rule out confounding effects of the extraneous variable. This argument is neither controversial nor our invention. The APA dictionary of psychology gives the following definition of control:

" the regulation of all extraneous conditions and variables in an experiment so that any change in the dependent variable can be attributed solely to manipulation of the independent variable and not to any other factors."

5) *Irrespective of control conditions in the RHI, we maintain that our sham control is adequate because it mimics all the aspects of the Lackner illusion procedure to the participant. Importantly, it doesn't reliably elicit the illusion. We think studying expectancies with this paradigm is a useful suggestion and we have now made a recommendation regarding this in the Discussion.*

That a control condition doesn't produce the same responses as the experimental condition is not evidence for its validity. The sham control here may well be a good match for expectancies. Or it may not. Please remove any assertions that expectancies were similar across sham and lackner conditions. E.g., that the stimulus was "apparently similar" across conditions.

6) *"First, if the effect of the hypnotic suggestion were caused by compliance in response to*

Demand characteristics, social desirability would be expected to correlate to some

extent with effects in the hypnotic condition, which is against the evidence.

Null relationships between response to an imaginative suggestion and social desirability scales do not provide evidence against cold control theory. Social desirability is not a good predictor of hypnotic responding. The claim in cold control theory is that response to imaginative suggestion is not reducible to social desirability.

E.g., Lush et al (2020) https://doi.org/10.1038/s41467-020-18591-6:

"Hypnotic responding is distinct from social compliance or social desirability, and is not attributable to mere compliance."

On a related note, cold control theory is described inaccurately throughout the manuscript (leading to some confusion in arguments). Cold control is a theory of hypnosis. Unless the authors define hypnosis as compliance (which they do not), this description doesn't make sense. It is inconsistent to describe cold control as compliance and other theories as not reducible to compliance.

For example, in the final paragraph, cold control is described as “compliance without awareness”. An accurate description of cold control here would be: “intentional imagination without awareness of intention”.

7) *Second,this interpretation predicts that pointing effect follows perceptual effect. Indeed, in this view participants might shift their perceptual reports in response to demand characteristic but correctly follow instructions in the pointing task. However, the opposite does not hold, as participants going as far as shifting their pointing in line with the suggestion should report at least similar elongation in the perceptual task. One consequence is that the distribution of differences between perceptual and pointing effects should be shifted towards positive values, with negative values only corresponding to pointing inaccuracies. However, the data suggest that it is not the case, with the discrepancy between perceptual and pointing effect being reasonably close to a gaussian distribution centred on zero.”*

This argument is not supported by any analyses. That aside, a simple explanation for any difference between the two tasks is differing expectancies, but given there was no evidence for an effect of the Lackner illusion on this measure, it may well have other issues.

8) *Response : We fully agree that this is an interesting hypothesis that warrants further attention. However, strong claims along these lines have been made in multiple papers…*

The authors list quotes from studies in which we describe testing a causal theory with correlational evidence to support their claim that we infer causation from correlation. Testing a causal theory with correlational evidence is not the same as inferring causation from correlation.

9) *As far as I can tell, it seems that one of the two measures of the Lackner illusion employed here may say survive sham control. This, to me, was among the more interesting aspects of the present manuscript. I think it would be worth bringing out more. We thank the reviewer for his interest in that part. The effect of the Lackner illusion on the perceived size of the finger is only a replication of previous studies (Lackner 1988, Burrack & Brugger 2005, Vignemont et al. 2005, Ehrsson et al. 2005) and therefore it is not very surprising. Since it is quite peripheral to our main question, we prefer not commenting more on that result.*

My intended point was that this could provide evidence that there is a Lackner illusion effect which might not be attributable to demand characteristics. For this to hold, expectancies for the Lackner and sham conditions would need to be shown to match on expectancies. I don't know how common sham controls are in Lackner illusion studies, but if this is not already well established it may be worth testing.

10) "the link between the two body representations and behavioural measures is definitional: body image is the body you perceive and thus variations in the perceptual task indexes changes in the body image."

This clarifies for me the arguments regarding hypnotic suggestion changing body schema. These arguments are straightforward if reports of changing body image are definitionally changes in body schema. However body schema are now not an explanation but a description. If body schema is simply a label applied to reports about body image, the inconsistency in the discussion section is resolved:

P.18 "These results demonstrate that hypnotic suggestion is an effective technique for transient modulations of body image and body schema."

And

P. 26 "The claim, however, is not that hypnotic suggestion engages the same basic perceptual processes as Lackner illusion. Instead, we discussed two interpretations of the effect, either low-level in terms of cognitive penetration, or high-level in terms of compliance without awareness."

The authors' definition of "body schema" should be established in the introduction, to reduce the risk of confusing readers who may have a different definition in mind (as I did).

11) As the study is not pre-registered, please clarify the exploratory nature of analyses.

Please state inferential rules followed in the analysis section. E.g., criteria for BF sensitivity (conventionally 3 and 1/3 or 6 and 1/6) and which inferences are based on which statistical approach (it seems that p values are interpreted for effects and Bayes factors for null effects, but sometimes this is deviated from.).
