# Supplementary material for: Hypnotic suggestion versus sensory modulation of bodily awareness
Source: PLoS One. 2023 Sep 12;18(9):e0291493. doi: 10.1371/journal.pone.0291493 (PMC10497174; doi:10.1371/journal.pone.0291493)
Supplement: Attachment — Submitted filename: Response to reviewers.docx [file pone.0291493.s003.docx]

**Response to Reviewers**

We thank the second reviewer for his additional comments. Our responses (in blue) are below, and we have also highlighted corresponding changes in the text.

**Reviewer #2:**

This manuscript presents data for a finger length perception task and a line reaching task in three conditions: sham control, Lackner illusion and a hypnotic suggestion for finger elongation (which is comparable to a classic hypnosis scale item for an experience of the hands being drawn together by an imaginary magnetic force). There is evidence for a Lackner effect larger than the effect in the sham condition, but interpretation of this difference is complicated by a lack of evidence that a confounding variable is matched across conditions). The authors appeal to differences in the relationships between trait response to hypnotic imaginative suggestion (hypnotisability) and perception measure response between the hypnotic suggestion and Lackner conditions to support arguments about mechanism. However, there is no evidence for or against a difference in these relationships. In my view, the simplest explanation for the difference between the Lackner illusion and hypnotic suggestion conditions is a difference in expectancies between the two conditions. The hypothesis-consistent demand characteristics seem to me likely to be clearer for the hypnotic suggestion than for the Lackner condition (because in the first case they are explicit and in the latter case implicit).

I did not review the results in detail in the first version of the manuscript. The results have now been extensively revised, and I will therefore focus this review the results and related claims in the discussion. Many of the claims in the discussion are based on invalid inferences, which I will detail below. I will focus on the perception measure because for the line-reaching measure there was no evidence for or against an effect in the Lackner condition, or evidence for a difference between sham control and Lackner condition. I will not review in detail any discussion which is based on invalid statistical inference.

I recommend major revision to address the problems I discuss below, including: remove invalid inferences and related discussion, to correct the definition of cold control theory and to clarify the definition of body schema employed in both the introduction and abstract.


1) Response : We agree with the reviewer that a non-significant p-value should not be interpreted as evidence supporting the null hypothesis. It is also true that using Bayes Factor in several instances rather than p-values allows for a stronger conclusions, and we thank the reviewer for this suggestion. On the other hand, we do think it is not inaccurate to state “did not significantly correlate” – please note that this is not a null claim (e.g., “did not correlate”) – only a claim that a significant effect was not observed. We do acknowledge that in a few cases we incorrectly omitted the word “significant” and we have made sure to include that as appropriate throughout. This is especially appropriate when we’re not interested in the null per se but only that a covariate did not significantly confound our results. We have now added Bayes factors for all analyses (see Table 1, Table 2, Table 3, Table 4 and Fig 4). We have now clearly stated that we are using default Bayes factors throughout.

I thank the authors for removing some invalid claims based on non-significant p values (e.g., that a non-significant result is surprising). However, there are many such claims in the discussion section of the revised manuscript. For example, claims of differences for significant and non-significant correlations between hypnotisability and either the Lackner illusion or hypnotic suggestion conditions. The only evidence for or against a relationship in these cases is a p value below .05 for a positive relationship between hypnotic suggestion response and hypnotisability.

p.19: “Weaker and non-significant correlations with hypnotic
suggestibility were observed for perceptual effect in the Lackner illusion and sham condition.”

p.19 “Our data do not exclude the possibility that hypnotic suggestibility accounts for variability in the Lackner illusion, although our sample size was not large enough to detect this association. Nevertheless, our results suggest that if this association were present, it would be rather weak.”

p.19 “This sensitivity to alterations of bodily experience across bottom-up
and top-down contexts is probably not reducible to hypnotic suggestibility since correlations with effects in the Lackner illusion condition were weak and unlikely to differ from zero.”

“Relatedly, the specific effect of the Lackner illusion cannot be explained by top-down processing since it survived the inclusion of hypnotic suggestibility as a covariate in our model of perceptual effect.”

We thank the referee for his careful reading of our paper. We did not intend to make null claims in these quotes, but we understand that a reader might interpret them as such. Hence, we removed these and other points related to weak and uncertain correlations to focus on the main results of this work.

Furthermore, to support claims of a difference between the correlations, a test of the difference is required. I calculated confidence intervals from the online data (the manuscript is lacking CIs and SEs). The (bootstapped) 95% CI for the Pearson coefficient between hypnotisability and Lackner illusion is [.02, .48 ] and for the relationship between hypnotisability and response to suggestion [.08, .51]. The CI for the hypnotisability/Lackner illusion coefficient contains the point value of the parameter estimate for the hypnotisability/suggestion coefficient. The data are simply too noisy to distinguish between these relationships. A larger sample would be required.

As mentioned above, this was removed.

There is no statistical evidence for or against a line reaching/pointing effect in the Lackner condition to support the following claims:

p.20 “Although the Lackner illusion did modify body image, we found that it did not significantly modify body schema. This dissociation aligns with similar results showing that action can be immune to bodily illusions in some circumstances”

p.21 “Body image and body schema can be sometimes dissociated, thus motivating the conceptual
distinction between the two bodily representations [51]. This seems to be confirmed by the dissociation between the effect of the Lackner illusion on body image, and the lack of effect on body schema.”

We agree that these parts of the discussion were based on ambiguous results. We rephrased these parts to show that we primarily base our discussion on the literature and not on our data alone, and we made clear that our analysis was insensitive (p.19): “Although the Lackner illusion modified body image, we did not find that it significantly modified body schema. It could be that we failed to observe a significant Lackner illusion effect on body schema because the effect was too noisy, too weak, or it could be that this illusion only influenced body image. The Bayesian evidence in favour of this effect was insensitive and thus we are unable to determine which of these outcomes led to the current results. Regardless, previous research has observed a dissociation showing that action can be immune to bodily illusions in some circumstances [14,77,55,56] (but not in others e.g., [78]). Dissociations like this motivate the conceptual distinction between the two bodily representations [52].”

19. Furthermore, the stimulation was apparently similar between the sham and the Lackner illusion procedure and the instructions were identical. The sham condition was thus able to provide a good control condition for confounding expectancies, as suggested by similar correlations with hypnotic suggestibility.

We added a statement (p.12): “No participant reported being aware that one of the conditions was a sham”


No justification for models of H1 has been included. This issue could be somewhat mitigated If robustness regions are reported (so that readers can see the range of models consistent with the inference). Bayes factors are only informative regarding the relative evidence for two theories. If the model of H1 is not reasonable, the Bayes factor is not useful, but the authors provide no argument as to why the chosen model of H1 is considered reasonable. There are earlier studies of the Lackner illusion which could inform the choice of model.

Insofar as there has not been any prior research on the use of suggestion to modulate response patterns with this illusion, we were unable to draw on previous research for model specification. We do not think that previous work on the Lackner illusion would be informative in this regard either. We now more clearly justify our use of a default BF in the analysis section and state that robustness tests did not change our conclusions (p. 12): “Robustness tests with a wider prior (a width of √2, i.e., twice the size of the original prior) reached similar conclusions and thus the report results do not seem to be highly dependent on the specific parameters chosen for the prior”. We still think this is useful and informative, particularly in aiding our interpretation of non-significant results.

Models of H1 have only been provided for post-hoc comparisons, and not for other analyses (e.g., correlational analyses).

Apologies, this has been added to the “analyses” section (p.12): “In correlations, we also used default priors, a stretched beta distribution with width 1.0 [71].”

No criterion is provided for the interpretation of Bayes factors.

This has been added to the “analyses” section: “We set an alpha error probability threshold at .05, with *p* values below this threshold interpreted as statistically significant. Bayes Factors (BF_10_) below .33 were interpreted to reflect moderate evidence in favour of the null hypothesis, those greater than 3.0 were taken to provide moderate evidence for the alternative hypothesis and in-between values were interpreted as being ambiguous [72].”

2) Response : We appreciate the reviewer’s comments on the interpretation of behavioural measurement in our study. This is an important issue, which is actually widely spread in cognitive neuroscience: how to go from behaviours to what’s inside the back box? In the case of bodily processing, since Head and Holmes (1911), there has been a clinical tradition to take performance in perceptual or motor tasks as evidence in favour of a deficit of corresponding bodily representations (e.g. Paillard, 1999; Sirigu et al., 1991; Schwoebel and Coslett, 2005). It has further been found that performance in perceptual and motor tasks can be selectively modulated (e.g., Kammers et al., 2009; Gadsby, 2017). Finally, they correspond to distinct neural networks (Berlucchi and Aglioti, 2010; Naito et al., 2016; Ho and Lenggenhager, 2021). There may be other interpretations for all these results, but there is so far no evidence showing that these tasks do not measure two distinct types of bodily representations. Consequently, we believe that the body schema/body image hypothesis remains empirically and conceptually grounded. We have discussed extensively this hypothesis in other works (Vignemont, 2011; Vignemont, 2018; Vignemont et al., 2021) and we do not believe that our arguments should be repeated in this paper.

I presume that in each of these other cases the experimental effects have been at least assumed to not be suggestion effects. That's not possible for a direct suggestion.
As a side note, I should clarify my comments on a body schema interpretation of the suggestion condition in my previous review. I was not saying you shouldn’t be free to make the claim that hypnotic suggestion changes body schema, but rather noting some responses I think this claim is likely to face from imaginative suggestion researchers.

Some of these studies plausibly included direct suggestions, others plausibly did not. The same goes for so-called indirect suggestions. The referee is raising a larger issue regarding the role of suggestion effects and demand characteristics in this literature. Although we think this is interesting and worthy of further study, it is beyond the scope of the present paper. We do briefly raise the issue in the Discussion of whether response patterns on these different tasks are partly influenced by suggestion and expectation effects and whether such effects could contribute to this observed dissociation.

3) We should also note that researchers routinely operationalize a term with a measure and then reference the term even though others might disagree (e.g., operationalizing phenomenological control as suggestibility and then interpreting the latter as the former). We also don’t think the analogies with past life regression or animal magnetism are relevant here just as we would never draw analogies between phenomenological control and animal magnetism.

This paragraph is not directly relevant to my review of this manuscript, but note that “phenomenological control is not “operationalized as suggestibility””. It is a label we use to refer to hypnotic suggestibility (and response to implicit or direct suggestion in contexts other than hypnosis) with the aim of avoiding misunderstandings arising from other terms.
E.g., Dienes et al (2022) https://psycnet.apa.org/doi/10.1037/cns0000230: “Phenomenological control is the ability to control subjective experience so that a constructed counterfactual state of affairs appears real. It is not a new concept. We as a community have been calling it hypnotizability (in the context of hypnotic inductions) or suggestibility. But “hypnosis” refers to sleep, which is unrelated to phenomenological control (e.g. Banyai & Hilgard, 1976); and “suggestibility” implies being manipulated contrary to one’s real intentions, a gullibility that is little related to hypnotic response (e.g. Coe, Kobayashi, & Howard, 1973; Moore, 1964). So in our laboratory, we have begun to refer to hypnotic suggestibility in a more general context as the capacity for phenomenological control and, correspondingly, hypnotic response as an example of phenomenological control.”
I do not consider reference to mesmerism or hypnotic suggestion effects to be irrelevant or inappropriate when discussing an experimental investigation of response to hypnotic suggestion. Neither my colleagues nor I would be offended by comparisons between phenomenological control and mesmerism. Phenomenological control is an explanation of mesmerism. We often discuss mesmerism alongside phenomenological control.

The point that we were making is that your research group operationalizes phenomenological control as responsiveness to direct verbal suggestions, which many researchers refer to as “suggestibility”. In a similar way we are using “body image” and “body schema” to refer to what is measured in our tasks. We were referring to mesmerism as a falsified hypothesis for therapeutic effects driven by expectation and suggestion effects – hence, it is why as a phenomenon we don’t think it’s a valuable analogy. Either way, this discussion is not pertinent to this paper.

4) We no longer think this debate is especially relevant to the current work
and we have shortened or omitted discussion of these points. It should be noted that
different expectancies in different conditions does not necessarily mean that an effect
(condition difference) is driven by expectancies…

We do not anywhere claim that expectancy studies show an effect is necessarily attributable to demand characteristics. I stated our argument in my first review: “Asynchrony is not a valid control in the RHI, simply because control conditions must be matched for all important factors, and expectancies are clearly an important factor when controlling for demand characteristic effects.”
An experiment which employs controls for a known extraneous variable which is unmatched on that extraneous variable cannot rule out confounding effects of the extraneous variable. This argument is neither controversial nor our invention. The APA dictionary of psychology gives the following definition of control:
“ the regulation of all extraneous conditions and variables in an experiment so that any change in the dependent variable can be attributed solely to manipulation of the independent variable and not to any other factors.”

This issue is outside the scope of this paper. We don’t disagree with the APA definition of course.

5) Irrespective of control conditions in the RHI, we maintain that our sham control is adequate because it mimics all the aspects of the Lackner illusion procedure to the participant. Importantly, it doesn’t reliably elicit the illusion. We think studying expectancies with this paradigm is a useful suggestion and we have now made a recommendation regarding this in the Discussion.

That a control condition doesn’t produce the same responses as the experimental condition is not evidence for its validity. The sham control here may well be a good match for expectancies. Or it may not. Please remove any assertions that expectancies were similar across sham and lackner conditions. E.g., that the stimulus was “apparently similar” across conditions.

We were not claiming that different responses in the two conditions present evidence for its validity. We have not mentioned anything about expectancies in this paradigm because they were not measured. We state that the two conditions were matched except for the location of the stimulation and we have also noted that none of the participants seemed to believe, or infer, that this condition was a sham (p.12): “No participant reported being aware that one of the conditions was a sham.”

6) “First, if the effect of the hypnotic suggestion were caused by compliance in response to
Demand characteristics, social desirability would be expected to correlate to some
extent with effects in the hypnotic condition, which is against the evidence.

Null relationships between response to an imaginative suggestion and social desirability scales do not provide evidence against cold control theory. Social desirability is not a good predictor of hypnotic responding. The claim in cold control theory is that response to imaginative suggestion is not reducible to social desirability.
E.g., Lush et al (2020) https://doi.org/10.1038/s41467-020-18591-6:
“Hypnotic responding is distinct from social compliance or social desirability, and is not attributable to mere compliance.”
On a related note, cold control theory is described inaccurately throughout the manuscript (leading to some confusion in arguments). Cold control is a theory of hypnosis. Unless the authors define hypnosis as compliance (which they do not), this description doesn’t make sense. It is inconsistent to describe cold control as compliance and other theories as not reducible to compliance.
For example, in the final paragraph, cold control is described as “compliance without awareness”. An accurate description of cold control here would be: “intentional imagination without awareness of intention”.

Our intention was in no point to misrepresent or argue against Cold control theory. We do not equate the first theoretical account (p. 21-22) with cold control theory, and we agree with the reviewer that the two theoretical positions are different. Our mention of cold control theory (p. 20) was only to present one theoretical account based on intentional action without conscious awareness: “However, this is not to say that participants were lying or being deceitful. Metacognitive theories of hypnosis [82,83] propose that this intentional alteration of responses can occur outside of awareness. One version of this theory [82] maintains that intentions are preserved during hypnosis whereas high-order thoughts representing these first order intentions are inaccessible, thus resulting in a lack of awareness of one’s own intention (for supporting evidence, see [84,85]). Regardless of awareness of intentions, which we did not measure in this study, our data suggest that compliance is not driven by compliance. If the effect of the hypnotic suggestion were driven by compliance in response to demand characteristics, social desirability would be expected to correlate with responses, at least in the hypnotic condition. However, we actually observed consistent Bayesian evidence in favour of the null hypothesis that social desirability, as indexed by two different measures, was unrelated to the observed effects in different conditions.”

7) Second,this interpretation predicts that pointing effect follows perceptual effect. Indeed, in this view participants might shift their perceptual reports in response to demand characteristic but correctly follow instructions in the pointing task. However, the opposite does not hold, as participants going as far as shifting their pointing in line with the suggestion should report at least similar elongation in the perceptual task. One consequence is that the distribution of differences between perceptual and pointing effects should be shifted towards positive values, with negative values only corresponding to pointing inaccuracies. However, the data suggest that it is not the case, with the discrepancy between perceptual and pointing effect being reasonably close to a gaussian distribution centred on zero.”

This argument is not supported by any analyses. That aside, a simple explanation for any difference between the two tasks is differing expectancies, but given there was no evidence for an effect of the Lackner illusion on this measure, it may well have other issues.

We discussed this in detail p. 17 (previous version), but we decided to remove these analyses and the corresponding point in the discussion as it diverted from the main result and did not add much to the null correlation with social desirability.

We didn’t measure expectancies in this experiment and so we cannot comment on their role beyond what we already state in the Discussion.

8) Response : We fully agree that this is an interesting hypothesis that warrants further attention. However, strong claims along these lines have been made in multiple papers…

The authors list quotes from studies in which we describe testing a causal theory with correlational evidence to support their claim that we infer causation from correlation. Testing a causal theory with correlational evidence is not the same as inferring causation from correlation.

Testing a causal theory with correlational evidence is fine – we were taking issue with causal claims made on the basis of correlational data, which should be tempered. This discussion is not directly pertinent to the present paper, so we don’t think it’s crucial to discuss this further.

9) As far as I can tell, it seems that one of the two measures of the Lackner illusion employed here may say survive sham control. This, to me, was among the more interesting aspects of the present manuscript. I think it would be worth bringing out more. We thank the reviewer for his interest in that part. The effect of the Lackner illusion on the perceived size of the finger is only a replication of previous studies (Lackner 1988, Burrack & Brugger 2005, Vignemont et al. 2005, Ehrsson et al. 2005) and therefore it is not very surprising. Since it is quite peripheral to our main question, we prefer not commenting more on that result.

My intended point was that this could provide evidence that there is a Lackner illusion effect which might not be attributable to demand characteristics. For this to hold, expectancies for the Lackner and sham conditions would need to be shown to match on expectancies. I don’t know how common sham controls are in Lackner illusion studies, but if this is not already well established it may be worth testing.

We understand this point, but we just think it’s best to leave this to future research as we did not measure expectancy in this study (for reasons stated in the paper) and we already highlight the potential roles of suggestion and expectation effects in different body representation distortions in the Discussion.

10) “the link between the two body representations and behavioural measures is definitional: body image is the body you perceive and thus variations in the perceptual task indexes changes in the body image.”

This clarifies for me the arguments regarding hypnotic suggestion changing body schema. These arguments are straightforward if reports of changing body image are definitionally changes in body schema. However body schema are now not an explanation but a description. If body schema is simply a label applied to reports about body image, the inconsistency in the discussion section is resolved:

P.18 “These results demonstrate that hypnotic suggestion is an effective technique for transient modulations of body image and body schema.”

And

P. 26 “The claim, however, is not that hypnotic suggestion engages the same basic perceptual processes as Lackner illusion. Instead, we discussed two interpretations of the effect, either low-level in terms of cognitive penetration, or high-level in terms of compliance without awareness.”

The authors’ definition of “body schema” should be established in the introduction, to reduce the risk of confusing readers who may have a different definition in mind (as I did).

Defining the body schema and the body image is a challenging task and we adopt here a standard functional definition that is widespread in this literature (see for instance the recent multidisciplinary volume on the topic edited by Ataria and coll., 2021): body schema for action and body image for perception. We have now made a stronger effort to ensure that both terms are clearly defined in the Introduction.

11) As the study is not pre-registered, please clarify the exploratory nature of analyses.

We added a statement in this regard at the end of the “analyses” section: “No part of the study procedures or analyses was pre-registered prior to the research being conducted and thus the analyses are exploratory.”

Please state inferential rules followed in the analysis section. E.g., criteria for BF sensitivity (conventionally 3 and 1/3 or 6 and 1/6) and which inferences are based on which statistical approach (it seems that p values are interpreted for effects and Bayes factors for null effects, but sometimes this is not the case).

This has been added to the “analyses” section: “We set an alpha error probability threshold at .05, with *p* values below this threshold interpreted as statistically significant. Bayes Factors (BF_10_) below .33 were interpreted to reflect moderate evidence in favour of the null hypothesis, those greater than 3.0 were taken to provide moderate evidence for the alternative hypothesis and in-between values were interpreted as being ambiguous [72]. We use p-values, effect sizes, and BFs for interpreting significant effects but only effect sizes and BFs for interpreting non-significant effects.”
