# Supplementary material for: Hypnotic suggestion versus sensory modulation of bodily awareness
Source: PLoS One. 2023 Sep 12;18(9):e0291493. doi: 10.1371/journal.pone.0291493 (PMC10497174; doi:10.1371/journal.pone.0291493)
Supplement: Attachment — Submitted filename: Response to reviewers.docx [file pone.0291493.s004.docx]

**Response to Reviewers**

We thank the second reviewer for his additional comments. Our responses (in blue) are below, and we have also highlighted corresponding changes in the text.

**Reviewer #2:**

This revised manuscript presents evidence for positive relationships between measures of trait hypnotisability and measures intended to reflect experience of an elongated finger for the Lackner illusion and a hypnotic suggestion for that experience. A null relationship between response and a measure of trait social compliance is also presented. The effect is stronger for the suggestion condition than for the Lackner condition. Because the sham control is not matched for demand characteristic effects with the Lackner condition, it is uninformative as to whether or not the Lackner illusion can be attributed to demand characteristics. A simple interpretation of these results is that both the Lackner illusion and the hypnotic suggestion are suggestion effects arising from phenomenological control. My interpretation of the difference in magnitude of effect between these conditions is as follows. In the hypnotic suggestion condition, demand characteristics will be relatively clear (because the participants are told what they should experience). In the Lackner condition, participants must work out what they are expected to experience themselves. The difference in effect between condition may reflect a resulting difference in the consistency of participant expectancies across conditions.

I thank the authors for addressing the issues I raised in the last review. However, there remain a couple of major inaccuracies which prevent the conclusions from following from both the presented data and existing evidence. There is also important information missing for Bayesian analyses. I will detail these below and provide some other brief comments which I hope will be useful.

1) On p.21 “If the effect of the hypnotic suggestion were driven by compliance in response to demand characteristics, social desirability would be expected to correlate with responses,”. This is incorrect. First, null relationships with social desirability measures do not provide evidence against demand characteristic effects (see e.g., Nichols & Maner, 2008). Second, the idea that response to imaginative suggestion is attributable to being controlled by a ‘hypnotist’ or being gullible or weak willed etc is a myth arising from the hypnotic context (e.g., see (Lynn et al., 2020.). As I stated in the last round of review, we employ the term ‘phenomenological control’ with the aim of avoiding misunderstandings of this kind which can arise from use of the term ‘suggestibility’. Response to imaginative suggestion is goal-directed and strategic. A null relationship between measures of social compliance and a given effect does not provide evidence against a cold control mechanism for that effect. These interpretations of social desirability results don’t hold and should be revised or removed.

We respectfully disagree with the reviewer. The reviewer seems to be misunderstanding what we’ve written and in particular the word ‘compliance’. We’re referring to simple compliance effects here, where participants overtly comply with the intended outcome without any actual changes in their experience. We’re not referring to suggestion effects, genuine changes in experience in response to the suggestion, or to what the author refers to as ‘phenomenological control’. This is precisely why we referred to “compliance” and not just demand characteristics, which can denote both simple compliance effects as well as genuine changes in experience. Similarly, we aren’t arguing for or against cold control theory – cold control theory isn’t relevant at all to this point. We’re not making any strong claim here – we’re only stating that a null correlation between social desirability and our observed effects (supported by Bayes factors) is consistent with the idea that the effects are not simple compliance effects. Indeed, this interpretation is consistent with the notion of phenomenological control and cold control theory (as well as other theories of hypnosis). We only base our conclusion on social desirability being representative of compliance, not all types of demand characteristics effects. Indeed, as the reviewer stated in a recent article (Corneille and Lush 2023), there are different strategies to meet the requirements of the situation, including behavioral compliance. Our conclusion here is that compliance is unlikely, and we think it is a reasonable conclusion.

Furthermore, we agree that response to suggestion is different from gullibility or being controlled by the hypnotist. These are well established results in the field. This has nothing to do with the quoted passage or our work.

2) It is asserted that there was “no systematic expectation” (p.19) for the Lackner illusion condition. I do not consider the claim on p.12 to be sufficient evidence against expectancy differences to support this assertion (“No participant reported being aware that one of the conditions was a sham.”). Whether there is “systematic expectation” for the Lackner condition is an open question. I think it is likely that there is. My position is informed by evidence for hypothesis awareness in a measure similar to the pointing task here in another embodiment illusion procedure (“proprioceptive drift”): https://royalsocietypublishing.org/doi/10.1098/rsos.210911 . In any case, there is no evidence provided here to decide this matter (and the claim is contradicted in subsequent paragraphs). This assertion should be removed.

Our reasoning is based on the sham manipulation being similar to the baseline condition (at least on the perceptual task). If systematic expectation were present, there should be a significant difference in the sham condition relative to baseline, which was not the case. We simply disagree on the sham being a valid control. The reviewer has not pointed to a specific difference between the sham and Lackner illusion to support their view and so we have left this statement in.

3) “Robustness tests with a wider prior (a width of √2,i.e., twice the size of the original prior) reached similar conclusions and thus the report results do not seem to be highly dependent on the specific parameters chosen for the prior.”

As requested in the previous round, robustness regions should be reported (alongside each Bayes factor). The sentence above is not a sufficient alternative. Robustness regions allow the reader to interpret Bayes factors according to their own model of H1. Because the analysis employs default models of H1, which are uniformed by evidence, RRs are of particular importance.

We can see the merit of reporting robustness regions and we updated the manuscript accordingly. We were not able to compute robustness regions for the Covariate effects in repeated-measures ANOVAs examining condition effects on responses in the finger length perception task and line reaching task. However, we do not consider this to be problematic as these analyses are only used to reject the hypothesis that each covariate significantly influences the effect of the conditions. We also removed the insensitive comparisons between correlations in the “correlations” section (p.17).

Other points:

• P.14, Ln 11: “By contrast”. Although this might be intended to only refer to a contrast in reference to sensitivity of Bayesian analysis, it could also be taken to mean a sensitive result contrasts meaningfully with an insensitive result. Because an insensitive result cannot be contrasted in this way (it provides no evidence for either H1 or H0 and is therefore uninformative), this second interpretation would not be valid. For this reason I recommend removing any reference to insensitive Bayes factors contrasting with sensitive Bayes factors.

We changed “by contrast” to “by comparison”.


• P.21 “When the suggested expectancies are unlikely for a participant, as might be expected for a low suggestible participant”
This claim lacks evidence. The relationship between expectancies and hypnotic responding does not indicate that low hypnotisables have particularly low expectancies (e.g., Benham et al, 2006).


This literature is more mixed than the reviewer implies, and some studies have indeed shown that low responsive participants do indeed have lower expectations (e.g., Lynn et al., 2008, Oxford handbook of hypnosis). On the other hand, we have several ongoing projects where the relationship between expectancy and response to suggestion shows heteroscedasticity, with low expectancies corresponding to no response and high expectancies corresponding to the whole range of responding. Nonetheless, this is not an important point for us, and we changed it for something less contentious (p21): “When the suggested experience is considered unlikely (low expectancy) by a participant, as might be the case for a difficult suggestion such as visual hallucination”
