# Supplementary material for: Hypnotic suggestion versus sensory modulation of bodily awareness
Source: PLoS One. 2023 Sep 12;18(9):e0291493. doi: 10.1371/journal.pone.0291493 (PMC10497174; doi:10.1371/journal.pone.0291493)
Supplement: Attachment — Submitted filename: Response to reviewers.docx [file pone.0291493.s005.docx]

**Response to Reviewers**

We thank the second reviewer for his additional comments. Our responses (in blue) are below, and we have also highlighted corresponding changes in the text.

**Reviewer #2:**

The authors have addressed several of my comments. However, the main points I raised in the last review round have not been satisfactorily addressed (the first has been addressed in the reviewer response, but not in the manuscript).

1)

a) “ If this effect were driven by compliance in response to demand characteristics, social desirability would be expected to correlate with responses, at least in the hypnotic condition.”

As I noted previously, trait social desirability is not a good predictor of demand characteristic effects e.g., (<https://www.tandfonline.com/doi/abs/10.3200/GENP.135.2.151-166>), and a null relationship between social desirability and an effect does not provide evidence that an effect is not attributable to demand characteristics (including compliance). The authors' response treats this claim more cautiously, but this is not reflected in the manuscript.

The evidence is actually mixed concerning the link between social desirability and demand characteristics, with some articles showing a positive correlation ([https://link.springer.com/article/10.1007/s00520-023-07761-z](https://emea01.safelinks.protection.outlook.com/?url=https%3A%2F%2Flink.springer.com%2Farticle%2F10.1007%2Fs00520-023-07761-z&data=05%7C01%7C%7C36771cd47c2b416085db08db90f6ed6a%7C84df9e7fe9f640afb435aaaaaaaaaaaa%7C1%7C0%7C638263162442156139%7CUnknown%7CTWFpbGZsb3d8eyJWIjoiMC4wLjAwMDAiLCJQIjoiV2luMzIiLCJBTiI6Ik1haWwiLCJXVCI6Mn0%3D%7C3000%7C%7C%7C&sdata=ICvI8x4mM68qpSMN24LgSOy6c4vayPUl4E2z7VZySvo%3D&reserved=0)). Note that we’re not claiming that this effect is thus not attributable to demand characteristics – only that an explanation in terms of basic compliance is rendered less likely. We have now amended the text as follows (p. 23): “If this effect were driven by compliance in response to the perceived requirements of the situation, social desirability would be expected to correlate with responses, at least in the hypnotic condition. Insofar as we observed a null correlation for this association, this suggests that our observed effects are unlikely to be driven by compliance, but we cannot rule out the possibility of broader effects related to demand characteristics.”

b) The authors have clarified that that their claim about social desirability is only intended to refer to compliance effects, and not to cold control theory. However, this has not been clarified in the manuscript, and the passage can still be read as a rejection of cold control theory in favour of a predictive processing theory of hypnosis. The unintended rejection of cold control implied by the structure could be avoided by presenting any arguments about compliance separately, rather than sandwiched in between discussion of the two theories.

We think this section is fairly clear since we’re clearly writing about compliance, which is a very distinct mechanism from that proposed by cold control theory. Nevertheless, we’re happy to move this to avoid any confusion. We moved this section later in the discussion (p.23)

2) The assertion on p.19 that there was “no systematic” expectation for Lackner illusion conditions has not been removed as requested. The authors justify retaining it with the following argument:

“If systematic expectation were present, there should be a significant difference in the sham condition relative to baseline, which was not the case.”

This is incorrect. Whether or not a difference between baseline and sham condition would be expected to arise from expectancies depends on the direction of the expectancies. I repeat the point made in my previous review - the evidence presented does not support this strong claim regarding a central issue. The claim should be removed, or evidence provided to support it (e.g., instruction-based replication: <https://doi.org/10.1525/collabra.82234>)

As suggested by the reviewer, we gathered data from an online survey. We report the results of this survey in the article p.10-11:

To ensure that expectations were similar between the sham manipulation and the Lackner illusion manipulation, we surveyed a group of 72 participants (online) that did not know of the Lackner illusion. Participants were described the manipulations and measurements with text and images, but the expected effect of the manipulation was not mentioned similarly to the study. Manipulations were labelled as “illusion 1” (sham) and “illusion 2” (Lackner illusion) for participants. They were then asked to estimate if the manipulation, compared to baseline, would make a person receiving the illusion “overshoot, pointing past the lines”, “correctly point to the lines”, or “undershoot, being short of reaching the lines”. This was done for both manipulations in the same order as the experiment, with the sham manipulation first then the Lackner illusion manipulation. Similar questions were asked about the effects in the finger length perception task with options: “perceive the index finger as shorter”, “perceive the index finger as normal”, “perceive the index finger as longer”. These analyses reveal that for the line reaching task expectancies are relatively balanced (no significant difference from .5) between manipulations, proportion *P*=.465 was not significantly different from .5 with *p*=.635, *B*=0.13 (See Table 1). However, this was not the case for the perceptual task where the sham manipulation was expected to lead to perceptual elongation more than the Lackner illusion, proportion *P*=.333 was significantly different from .5 with *p*=.007, *B*=4.45 (See Table 1). Overall, we are confident that the sham manipulation played its role as a control condition.

3) RRs for Table 4,: it should be made explicit that all the RR values allow the full range of possible correlations, so the evidence for H0 is overstated.

To illustrate, the 95% CI for the correlation between the Lackner illusion perceptual effect and 'SDE' is between approximately r = -.10 and r = .40. I consider a .4 correlation to be far from evidence for no relationship.

Table 4 also does not meet minimum reporting standards as there is no measure of dispersion (SE, SD or 95% CI). While it is necessary to provide some measure of dispersion, I strongly recommend presenting 95% CIs (in addition to SD or SE) so that the reader can easily interpret the results and decide for themselves whether they agree with the presented interpretation.

We added the 95% CIs, as recommended. The comment on RR and evidence for H0 being overstated comes down to the prior distribution selected (a stretched beta distribution). However, we put a reminder under Table 4 (now Table 5) to help the reader in interpreting RR (p.19): Bs and RR are derived from a stretched beta distribution prior with width 1.0

Note - the data file does not contain DVs for some measures (e.g., BIDR). While these can be calculated, PLOS policy requires they be included.

This has been changed in the data file.
