# Supplementary material for: Hypnotic suggestion versus sensory modulation of bodily awareness
Source: PLoS One. 2023 Sep 12;18(9):e0291493. doi: 10.1371/journal.pone.0291493 (PMC10497174; doi:10.1371/journal.pone.0291493)
Supplement: Peer review history

# Peer review: Decision Letter 0

**DOI:** 10.1371/journal.pone.0291493.r001

**Authors:** Jane Elizabeth Aspell

10 Jan 2023

PONE-D-22-33125Hypnotic suggestion versus sensory modulation of bodily awarenessPLOS ONE

Dear Dr. Apelian,

Thank you for submitting your manuscript to PLOS ONE. After careful consideration, we feel that it has merit but does not fully meet PLOS ONE’s publication criteria as it currently stands. Therefore, we invite you to submit a revised version of the manuscript that addresses the points raised by the two reviewers.

Please submit your revised manuscript by Feb 24 2023 11:59PM. If you will need more time than this to complete your revisions, please reply to this message or contact the journal office at plosone@plos.org. When you're ready to submit your revision, log on to https://www.editorialmanager.com/pone/ and select the 'Submissions Needing Revision' folder to locate your manuscript file.

Please include the following items when submitting your revised manuscript:

- A rebuttal letter that responds to each point raised by the academic editor and reviewer(s). You should upload this letter as a separate file labeled 'Response to Reviewers'.
- A marked-up copy of your manuscript that highlights changes made to the original version. You should upload this as a separate file labeled 'Revised Manuscript with Track Changes'.
- An unmarked version of your revised paper without tracked changes. You should upload this as a separate file labeled 'Manuscript'.

If you would like to make changes to your financial disclosure, please include your updated statement in your cover letter. Guidelines for resubmitting your figure files are available below the reviewer comments at the end of this letter.

If applicable, we recommend that you deposit your laboratory protocols in protocols.io to enhance the reproducibility of your results. Protocols.io assigns your protocol its own identifier (DOI) so that it can be cited independently in the future. For instructions see: https://journals.plos.org/plosone/s/submission-guidelines#loc-laboratory-protocols. Additionally, PLOS ONE offers an option for publishing peer-reviewed Lab Protocol articles, which describe protocols hosted on protocols.io. Read more information on sharing protocols at https://plos.org/protocols?utm_medium=editorial-email&utm_source=authorletters&utm_campaign=protocols.

We look forward to receiving your revised manuscript.

Kind regards,

Jane Elizabeth Aspell, PhD

Academic Editor

PLOS ONE

Journal Requirements:

When submitting your revision, we need you to address these additional requirements.

1. Please ensure that your manuscript meets PLOS ONE's style requirements, including those for file naming. The PLOS ONE style templates can be found at 

https://journals.plos.org/plosone/s/file?id=wjVg/PLOSOne_formatting_sample_main_body.pdf and 

https://journals.plos.org/plosone/s/file?id=ba62/PLOSOne_formatting_sample_title_authors_affiliations.pdf

2. Please provide additional details regarding ethical approval in the body of your manuscript. In the Methods section, please ensure that you have specified the name of the IRB/ethics committee that approved your study.

3. Please provide additional details regarding participant consent. In the Methods section, please ensure that you have specified (1) whether consent was informed and (2) what type you obtained (for instance, written or verbal). If your study included minors, state whether you obtained consent from parents or guardians. If the need for consent was waived by the ethics committee, please include this information.

4. Please change "female” or "male" to "woman” or "man" as appropriate, when used as a noun (see for instance https://apastyle.apa.org/style-grammar-guidelines/bias-free-language/gender).

5. We note that the grant information you provided in the ‘Funding Information’ and ‘Financial Disclosure’ sections do not match. 

When you resubmit, please ensure that you provide the correct grant numbers for the awards you received for your study in the ‘Funding Information’ section.

6. Thank you for stating the following in the Competing Interests section: 

"A.C have read the journal's policy and the authors of this manuscript have the following competing interests: scholarship from ARCHE formation. T.D. and V.F. have declared that no competing interests exist."

We note that one or more of the authors have an affiliation to the commercial funders of this research study: ARCHE formation

(1) Please provide an amended Funding Statement declaring this commercial affiliation, as well as a statement regarding the Role of Funders in your study. If the funding organization did not play a role in the study design, data collection and analysis, decision to publish, or preparation of the manuscript and only provided financial support in the form of authors' salaries and/or research materials, please review your statements relating to the author contributions, and ensure you have specifically and accurately indicated the role(s) that these authors had in your study. You can update author roles in the Author Contributions section of the online submission form.

Please also include the following statement within your amended Funding Statement. 

“The funder provided support in the form of salaries for authors [insert relevant initials], but did not have any additional role in the study design, data collection and analysis, decision to publish, or preparation of the manuscript. The specific roles of these authors are articulated in the ‘author contributions’ section.”

If your commercial affiliation did play a role in your study, please state and explain this role within your updated Funding Statement. 

(2) Please also provide an updated Competing Interests Statement declaring this commercial affiliation along with any other relevant declarations relating to employment, consultancy, patents, products in development, or marketed products, etc.  

Within your Competing Interests Statement, please confirm that this commercial affiliation does not alter your adherence to all PLOS ONE policies on sharing data and materials by including the following statement: ""This does not alter our adherence to  PLOS ONE policies on sharing data and materials.” (as detailed online in our guide for authors http://journals.plos.org/plosone/s/competing-interests). If this adherence statement is not accurate and  there are restrictions on sharing of data and/or materials, please state these. Please note that we cannot proceed with consideration of your article until this information has been declared.

Please include both an updated Funding Statement and Competing Interests Statement in your cover letter. We will change the online submission form on your behalf.

7. Please include your full ethics statement in the ‘Methods’ section of your manuscript file. In your statement, please include the full name of the IRB or ethics committee who approved or waived your study, as well as whether or not you obtained informed written or verbal consent. If consent was waived for your study, please include this information in your statement as well. 

[Note: HTML markup is below. Please do not edit.]

Reviewers' comments:

Reviewer's Responses to Questions

**Comments to the Author**

1. Is the manuscript technically sound, and do the data support the conclusions?

The manuscript must describe a technically sound piece of scientific research with data that supports the conclusions. Experiments must have been conducted rigorously, with appropriate controls, replication, and sample sizes. The conclusions must be drawn appropriately based on the data presented. 

Reviewer #1: Yes

Reviewer #2: No

**********

2. Has the statistical analysis been performed appropriately and rigorously? 

Reviewer #1: Yes

Reviewer #2: No

**********

3. Have the authors made all data underlying the findings in their manuscript fully available?

The PLOS Data policy requires authors to make all data underlying the findings described in their manuscript fully available without restriction, with rare exception (please refer to the Data Availability Statement in the manuscript PDF file). The data should be provided as part of the manuscript or its supporting information, or deposited to a public repository. For example, in addition to summary statistics, the data points behind means, medians and variance measures should be available. If there are restrictions on publicly sharing data—e.g. participant privacy or use of data from a third party—those must be specified.

Reviewer #1: Yes

Reviewer #2: Yes

**********

4. Is the manuscript presented in an intelligible fashion and written in standard English?

PLOS ONE does not copyedit accepted manuscripts, so the language in submitted articles must be clear, correct, and unambiguous. Any typographical or grammatical errors should be corrected at revision, so please note any specific errors here.

Reviewer #1: Yes

Reviewer #2: Yes

**********

5. Review Comments to the Author

Please use the space provided to explain your answers to the questions above. You may also include additional comments for the author, including concerns about dual publication, research ethics, or publication ethics. (Please upload your review as an attachment if it exceeds 20,000 characters)

Reviewer #1: The study investigated how cognitive (hypnotic suggestion) versus sensory manipulations influence bodily awareness. The experiment appears to be well-designed, the data visualization is clear, and the data itself have been made open in a public repository, which are all solid strengths of the study. The statistical analyses are described in sufficient detail, and the conclusions are presented in an appropriate fashion and are supported by the data. There are a few minor issues that should be addressed before proceeding with publication of this study, which I pointed out below, hoping they are relevant for the authors to improve their manuscript.

Page 4, line 26:

The peer-reviewed version of the paper can be found here: Lush, P., Botan, V., Scott, R. B., Seth, A. K., Ward, J., & Dienes, Z. (2020). Trait phenomenological control predicts experience of mirror synaesthesia and the rubber hand illusion. Nature communications, 11(1), 1-10.

Page 5, line 6:

Given the context of the subsequent sentence, I believe the authors wanted to refer to the following paper: Lush, P., Botan, V., Scott, R. B., Seth, A. K., Ward, J., & Dienes, Z. (2020). Trait phenomenological control predicts experience of mirror synaesthesia and the rubber hand illusion. Nature communications, 11(1), 1-10. (Although Lush, 2020 is also an example of a study on RHI and hypnotic suggestibility)

Page 5, line 7:

The peer-reviewed version of the paper can be found here: Ehrsson, H. H., Fotopoulou, A., Radziun, D., Longo, M. R., & Tsakiris, M. (2022). No specific relationship between hypnotic suggestibility and the rubber hand illusion. Nature communications, 13(1), 1-3.

For a further insight in the investigation of the potential influence of hypnotic suggestibility on the RHI, the authors could consider discussing the following article in the Introduction and/or Discussion: Slater, M., & Ehrsson, H. H. (2022). Multisensory integration dominates hypnotisability and expectations in the rubber hand illusion. Frontiers in Human Neuroscience, 317.

Reviewer #2: This manuscript presents evidence for an influence of a hypnotic suggestion and a vibrating stimulus applied to the arm for two measures of perceived finger length. I find the initial concept very interesting (my colleagues and I have also been testing direct suggestion for other body illusions recently) and it is great to see the use of a sham control (though claims regarding this would be strengthened by a test of expectancies for the two conditions in a separate sample). The manuscript is appropriately structured and the standard of English is good. However, major conclusions (in particular regarding body schema and image), are not sufficiently supported by the data and there are a number of major problems with the presented analyses and their interpretation (e.g., non-significant p values have been used to support the null). In addition, insufficient detail has been provided for the mixed linear models. The authors may be able to address these issues in a revision, and I have provided some detailed consideration of each which I hope may be useful. Because this manuscript closely relates to much of our recent research, I have referenced papers by my colleagues and I and sign my review in the interests of transparency.

Null results and Bayes factors

Although there are Bayesian tests of some null results there are also many instances in which a non-significant p value has been erroneously interpreted as evidence of no difference.

E.g., “although the model did not improve if we allowed for an interaction between hypnotic suggestibility and condition, χ²(2,N=50)=3.28, p=.19.”

Please add Bayes factors in each case, or revise the text to clarify that no conclusions have been drawn from these non-significant p values. Also revise discussion text relating to assertion of the null based on non-significant p values (e.g., p.21 “Somewhat unexpectedly, familiarity with hypnosis did not significantly correlate with hypnotic suggestibility. Some correlation was…”). I have given only a couple of examples, but there are other similar examples throughout.

Please provide the model for H1 for the Bayes factors. They are uninterpretable without this information. If a default model was used, it’s relevance to the predicted effect size should be argued. If it is not relevant, a relevant prediction should be used. I find Dienes (2019) helpful for specifying predicted raw effects.

Other analyses

I found the analyses very difficult to follow. A more detailed explanation of why particular analytical decisions were taken would be helpful. I did not find it possible to adequately assess the results in their present form. There seems to be a mixture of approaches. For example, why are there correlations in addition to the mixed linear models? I recommend sticking to one approach throughout. For example,

(1) use ANOVAs and correlations on participant means, or (2) use LMMs for condition effects and corrrelations, and assess significance with t-tests on the coefficients, or (3) use LMMs for condition effects and correlations and assess significance using likelihood ratio tests

In addition please:

Report the LMM in R notation, including how each condition was coded (e.g. sham = 1, not sham = 0)".

Specify what the betas are in table 1. Are they contrasts? If so, please ensure the LMM specification (given in bold) matches the parameters in the table

For each statistical test, please make explicit what the DV is. For example, what are the t-tests in BFs in paragraph 2 of section 3.2 calculated on? If they were calculated on betas, please explain that the LMMs were refitted separately for each level of Condition Order

Conclusions regarding body image and schema

Measures are conflated with the constructs they are posited to reflect; the names of two tasks are treated as interchangeable with “body schema” and “body image”, leading to overstatements in the central interpretation of results, e.g., p. 18 “These results demonstrate that the hypnotic suggestion is an effective technique for transient modulations of body image and body schema.”; p.19 “The hypnotic suggestion also had a significant effect on body schema.”. The presented arguments do not provide adequate support for claims that, for example, hypnotic suggestion modifies body schema. This issue could be addressed by referring to the measures directly rather than to the posited constructs, except when attempting to justify the link between the two through argument (e.g., p.23-25) and by removing, toning down or qualifying conclusions regarding body schema and image throughout.

Claims that response to an imaginative suggestion for a particular experience necessarily involves particular processes posited to underly similar experiences in other contexts are unsafe. As acknowledged in the discussion (p. 23 & 24), response to imaginative suggestion can be both interpretative and imaginative. Few would argue that the examples given in the introduction of hypnotically induced amnesia and blindness necessarily occur by the same mechanisms as other cases of amnesia and blindness. That an imaginative suggestion for an experience of finger lengthening involves changes in body image or schema seems to me little different than the claim that a moving hands together suggestion (e.g., in the WSGC) reveals an innate magnetic sense. I think the approach taken opens rather a large can of worms - hypnosis has a long history of such claims. While in some cases these are too implausible to be taken seriously (past life regression) in others they have been taken seriously and caused considerable problems (e.g., ‘recovered’ memories).

The following justification for reverse inference is given on p.25: “Caution is advised in interpreting results of behavioural tests as indicators of latent unobservable variables (Bach et al., 2018). Nonetheless, our claim that these behavioural tests are credible proxies of body image and body schema rests on current practices of the community(...) It follows that this inverse inference problem is rather a trivial limitation, meaning that the association between our behavioural indicators and body metrics is provisional and will depend on future development of the field.”

If my interpretation of this argument is correct, I do not find it convincing. A mistake being common does not indicate that it is trivial (I would argue the contrary). I don’t believe the experimental design can test “whether hypnotic suggestion can effectively modulate body image and/or body schema”. Response to these pointing and perceptual tasks might be taken to indicate relevant experience, but that such experience involves changes in “body image” or “body schema” is just one possibility, just as it is only one possibility among many that a subject’s regressed memories of life as Cleopatra are veridical. There are very good reasons to tread carefully here – the range of potential ‘discoveries’ which might arise from the application of reverse inference to response to imaginative suggestion is potentially vast.

p.5 “Furthermore, demand characteristics – namely participant’s explicit

compliance, or unconscious changes in behaviour and experience in accordance, with implicit or explicit experimental aims (Orne, 1962) – were recently debated as a potential contributing factor to the RHI (Lush, 2020). Effects turned out to be weak and unsignificant when relevant controls were applied (Ehrsson et al., 2021).”

p.19 “ (for the RHI, it was shown that with adequate control, the association was insignificant: Ehrsson et al., 2021).”

There is some confusion here. Lush (2020) showed that expectancies differ for the asynchronous control and synchronous illusion conditions of RHI studies. (see also replications in Reader 2022; Lush, Seth & Dienes, 2022). Asynchrony is not a valid control in the RHI, simply because control conditions must be matched for all important factors, and expectancies are clearly an important factor when controlling for demand characteristic effects. Ehrsson et al (2021) did not address the results of Lush (2020), but the results of Lush et al, (2020) which reported relationships between synchronous condition rubber hand illusion measures and hypnotisability, and also evidence for no relationship between illusion agreement scores and hypnotisability when ‘controlled’ by the asynchronous condition. Ehrsson et al reproduced this latter null result The interpretation of this null result rests on the validity of the control condition. Ehrsson et al assert that the control is valid, without evidence or argument. Similarly, in the manuscript, the control condition is described as ‘relevant’ and ‘adequate’, but no argument or reference for why it should be considered so has been provided. Please either provide an argument as to why this control procedure which is confounded by expectancies should be considered a valid control for demand characteristic effects or remove or revise this assertion. Rather than repeat our interpretation of this null result and related arguments here, I refer the authors to the arguments presented in our commentary published alongside Ehrsson et al (2021): Lush & Seth (2021) and in the original paper (Lush et al, 2020). In anticipation of a possible response, it is worth noting that a recent argument about the manipulation of expectancies in the original paper (deVignemont, 2022) is based on a misreading of our study design. It should also be noted that, even if the RHI control condition were valid, it would not provide good support for the claim that demand characteristics are not involved in the effects presented in this paper (in order to attribute the effects to changes in body schema etc).

P.20, section 4.2.

The simple alternative theory that differences in effects between illusion and suggestion conditions may arise from differing demand characteristics is treated inconsistently in this section. While it is considered here as a possible explanation for order effects (as also may occur in the RHI, see Lush, 2021), it may also explain other results and should be consistently applied to conclusions. For example, the relatively clear demand characteristics in the hypnotic suggestion condition may explain the correlation between measures in the suggestion condition in contrast with the illusion condition (though please ensure all claims of null results are backed by analyses other than non-significant p values - see below).

A further related issue - the indirect measures bear surface similarity to proprioceptive drift in the RHI (which is confounded by demand characteristics; Lush, Seth & Dienes, 2021) and may have similar problems.

“However, in contrast with claims made by some of these authors, a correlation between hypnotic suggestibility and a bodily illusion does not necessarily entail that an illusion is driven by suggestion and expectations.”

Please clearly state which of the papers contain this claim. Without direct reference, it is a strawman (I don’t recall seeing this argument in any of the cited papers, but I could well have missed it). As an aside, regarding the plausibility of suggestion effects in the rubber hand illusion, I think it is a mistake to consider this one illusion in isolation – similar relationships have been shown for mirror touch synaesthesia pain, ASMR and visually evoked auditory response. While other mechanisms may be in play in each case, that these relationships are due to imaginative suggestion effects arising from demand characteristics presents a simple, testable theory.

P.22. “ In this framework, the hypnotic suggestions did not directly alter body representations; rather, participants changed their behaviour and experience according to their expectations...”

This paragraph is a good summary which matches my interpretation of the results. I don’t see anything in these data which is not consistent with this position. While it is only one interpretation, that it can be applied to the data presented without issue highlights that the conclusions regarding body schema etc are an overstatement of the presented results.

Other points

As far as I can tell, it seems that one of the two measures of the Lackner illusion employed here may say survive sham control. This, to me, was among the more interesting aspects of the present manuscript. I think it would be worth bringing out more.

P.8 “By comparing the reported finger size at rest to the actual finger size, we can derive an estimate of the validity of the measure.”

I think I get the intended meaning, but the use of “validity” here is inadvisable given the specific meanings of the term in psychological science.

P.23 “supported by imagery and selective attention”

It might be worth noting evidence against trait differences in attention in response to imaginative suggestion here (see Martin & Pacherie, 2018, for example).

P.24 “We did not index response expectancies because doing so may provide participants with explicit indicators of how they are expected to respond”

These could be checked in a separate sample, e.g., to address (possibly) differing results between hypnosis and illusion conditions (I’m not suggesting you should do this now, btw).

Results are sometimes reported in cm and sometimes in mm. Better to choose one or the other throughout.

Open data – the variables in the provided file are difficult to interpret without a key. For example, what do the ‘pinocchio’ columns refer to?

Signed

P Lush

 

Dienes, Z. (2019). How do I know what my theory predicts?. Advances in Methods and Practices in Psychological Science, 2(4), 364-377.

Lush, P. (2021, July 19). Order effects in the rubber hand illusion. https://doi.org/10.31234/osf.io/amsrp

Lush, P., Seth, A. K., & Dienes, Z. (2021). Hypothesis awareness confounds asynchronous control conditions in indirect measures of the rubber hand illusion. Royal Society open science, 8(11), 210911.

Lush, P., & Seth, A. K. (2022). Reply to: no specific relationship between hypnotic suggestibility and the rubber hand illusion. Nature communications, 13(1), 1-3.

Reader, A. T. (2021). What do participants expect to experience in the rubber hand illusion? A conceptual replication of Lush (2020).

**********

6. PLOS authors have the option to publish the peer review history of their article (what does this mean?). If published, this will include your full peer review and any attached files.

If you choose “no”, your identity will remain anonymous but your review may still be made public.

**Do you want your identity to be public for this peer review?** For information about this choice, including consent withdrawal, please see our Privacy Policy.

Reviewer #1: No

Reviewer #2: **Yes: **Peter Lush

**********

[NOTE: If reviewer comments were submitted as an attachment file, they will be attached to this email and accessible via the submission site. Please log into your account, locate the manuscript record, and check for the action link "View Attachments". If this link does not appear, there are no attachment files.]

While revising your submission, please upload your figure files to the Preflight Analysis and Conversion Engine (PACE) digital diagnostic tool, https://pacev2.apexcovantage.com/. PACE helps ensure that figures meet PLOS requirements. To use PACE, you must first register as a user. Registration is free. Then, login and navigate to the UPLOAD tab, where you will find detailed instructions on how to use the tool. If you encounter any issues or have any questions when using PACE, please email PLOS at figures@plos.org. Please note that Supporting Information files do not need this step.

# Author comment: Author response to Decision Letter 0

**DOI:** 10.1371/journal.pone.0291493.r002

24 Mar 2023

Reviewer #1:

The study investigated how cognitive (hypnotic suggestion) versus sensory manipulations influence bodily awareness. The experiment appears to be well-designed, the data visualization is clear, and the data itself have been made open in a public repository, which are all solid strengths of the study. The statistical analyses are described in sufficient detail, and the conclusions are presented in an appropriate fashion and are supported by the data. There are a few minor issues that should be addressed before proceeding with publication of this study, which I pointed out below, hoping they are relevant for the authors to improve their manuscript.

Page 4, line 26: The peer-reviewed version of the paper can be found here: Lush, P., Botan, V., Scott, R. B., Seth, A. K., Ward, J., & Dienes, Z. (2020). Trait phenomenological control predicts experience of mirror synaesthesia and the rubber hand illusion. Nature communications, 11(1), 1-10.

Response : We have corrected this error – thanks. 

Reviewer #1:

Page 5, line 6: Given the context of the subsequent sentence, I believe the authors wanted to refer to the following paper: Lush, P., Botan, V., Scott, R. B., Seth, A. K., Ward, J., & Dienes, Z. (2020). Trait phenomenological control predicts experience of mirror synaesthesia and the rubber hand illusion. Nature communications, 11(1), 1-10. (Although Lush, 2020 is also an example of a study on RHI and hypnotic suggestibility)

Response : We substituted the suggested reference, this is indeed more relevant.

Reviewer #1:

Page 5, line 7: The peer-reviewed version of the paper can be found here: Ehrsson, H. H., Fotopoulou, A., Radziun, D., Longo, M. R., & Tsakiris, M. (2022). No specific relationship between hypnotic suggestibility and the rubber hand illusion. Nature communications, 13(1), 1-3.

For a further insight in the investigation of the potential influence of hypnotic suggestibility on the RHI, the authors could consider discussing the following article in the Introduction and/or Discussion: Slater, M., & Ehrsson, H. H. (2022). Multisensory integration dominates hypnotisability and expectations in the rubber hand illusion. Frontiers in Human Neuroscience, 317.

Response : We corrected the version error and added the suggested article. However, given the comments of reviewer 2 and the fact that this discussion is not central to the paper, we shortened the discussion around the RHI (pp.4) “Furthermore, it has been recently debated to what extent the RHI could be explained by demand characteristics [44,48,49], that is, by participants’ explicit compliance, or unconscious changes in behaviour and experience in accordance with implicit or explicit experimental aims [50]”

Reviewer #2:

This manuscript presents evidence for an influence of a hypnotic suggestion and a vibrating stimulus applied to the arm for two measures of perceived finger length. I find the initial concept very interesting (my colleagues and I have also been testing direct suggestion for other body illusions recently) and it is great to see the use of a sham control (though claims regarding this would be strengthened by a test of expectancies for the two conditions in a separate sample). The manuscript is appropriately structured and the standard of English is good. However, major conclusions (in particular regarding body schema and image), are not sufficiently supported by the data and there are a number of major problems with the presented analyses and their interpretation (e.g., non-significant p values have been used to support the null). In addition, insufficient detail has been provided for the mixed linear models. The authors may be able to address these issues in a revision, and I have provided some detailed consideration of each which I hope may be useful. Because this manuscript closely relates to much of our recent research, I have referenced papers by my colleagues and I and sign my review in the interests of transparency.

Null results and Bayes factors

Although there are Bayesian tests of some null results there are also many instances in which a non-significant p value has been erroneously interpreted as evidence of no difference.

E.g., “although the model did not improve if we allowed for an interaction between hypnotic suggestibility and condition, χ²(2,N=50)=3.28, p=.19.” Please add Bayes factors in each case, or revise the text to clarify that no conclusions have been drawn from these non-significant p values. Also revise discussion text relating to assertion of the null based on non-significant p values (e.g., p.21 “Somewhat unexpectedly, familiarity with hypnosis did not significantly correlate with hypnotic suggestibility. Some correlation was…”). I have given only a couple of examples, but there are other similar examples throughout. Please provide the model for H1 for the Bayes factors. They are uninterpretable without this information. If a default model was used, it’s relevance to the predicted effect size should be argued. If it is not relevant, a relevant prediction should be used. I find Dienes (2019) helpful for specifying predicted raw effects.

Response : We agree with the reviewer that a non-significant p-value should not be interpreted as evidence supporting the null hypothesis. It is also true that using Bayes Factor in several instances rather than p-values allows for a stronger conclusions, and we thank the reviewer for this suggestion. On the other hand, we do think it is not inaccurate to state “did not significantly correlate” – please note that this is not a not a null claim (e.g., “did not correlate”) – only a claim that a significant effect was not observed. We do acknowledge that in a few cases we incorrectly omitted the word “significant” and we have made sure to include that as appropriate throughout. This is especially appropriate when we’re not interested in the null per se but only that a covariate did not significantly confound our results. We have now added Bayes factors for all analyses (see Table 1, Table 2, Table 3, Table 4 and Fig 4). We have now clearly stated that we are using default Bayes factors throughout.

Reviewer #2:

Other analyses

I found the analyses very difficult to follow. A more detailed explanation of why particular analytical decisions were taken would be helpful. I did not find it possible to adequately assess the results in their present form. There seems to be a mixture of approaches. For example, why are there correlations in addition to the mixed linear models? I recommend sticking to one approach throughout. For example, (1) use ANOVAs and correlations on participant means, or (2) use LMMs for condition effects and corrrelations, and assess significance with t-tests on the coefficients, or (3) use LMMs for condition effects and correlations and assess significance using likelihood ratio tests In addition please: Report the LMM in R notation, including how each condition was coded (e.g. sham = 1, not sham = 0)". Specify what the betas are in table 1. Are they contrasts? If so, please ensure the LMM specification (given in bold) matches the parameters in the table For each statistical test, please make explicit what the DV is. For example, what are the t-tests in BFs in paragraph 2 of section 3.2 calculated on? If they were calculated on betas, please explain that the LMMs were refitted separately for each level of Condition Order

We thank the reviewer for these suggestions, which have helped us to clarify the results. We opted for the first suggested method as readers might be most familiar with it. The new result section is in line with what was done before. It should be noted that it did not change the main results and conclusions.

Conclusions regarding body image and schema

Measures are conflated with the constructs they are posited to reflect; the names of two tasks are treated as interchangeable with “body schema” and “body image”, leading to overstatements in the central interpretation of results, e.g., p. 18 “These results demonstrate that the hypnotic suggestion is an effective technique for transient modulations of body image and body schema.”; p.19 “The hypnotic suggestion also had a significant effect on body schema.”. The presented arguments do not provide adequate support for claims that, for example, hypnotic suggestion modifies body schema. This issue could be addressed by referring to the measures directly rather than to the posited constructs, except when attempting to justify the link between the two through argument (e.g., p.23-25) and by removing, toning down or qualifying conclusions regarding body schema and image throughout.

Claims that response to an imaginative suggestion for a particular experience necessarily involves particular processes posited to underly similar experiences in other contexts are unsafe. As acknowledged in the discussion (p. 23 & 24), response to imaginative suggestion can be both interpretative and imaginative. Few would argue that the examples given in the introduction of hypnotically induced amnesia and blindness necessarily occur by the same mechanisms as other cases of amnesia and blindness. That an imaginative suggestion for an experience of finger lengthening involves changes in body image or schema seems to me little different than the claim that a moving hands together suggestion (e.g., in the WSGC) reveals an innate magnetic sense. I think the approach taken opens rather a large can of worms - hypnosis has a long history of such claims. While in some cases these are too implausible to be taken seriously (past life regression) in others they have been taken seriously and caused considerable problems (e.g., ‘recovered’ memories).

The following justification for reverse inference is given on p.25: “Caution is advised in interpreting results of behavioural tests as indicators of latent unobservable variables (Bach et al., 2018). Nonetheless, our claim that these behavioural tests are credible proxies of body image and body schema rests on current practices of the community(...) It follows that this inverse inference problem is rather a trivial limitation, meaning that the association between our behavioural indicators and body metrics is provisional and will depend on future development of the field.” If my interpretation of this argument is correct, I do not find it convincing. A mistake being common does not indicate that it is trivial (I would argue the contrary). I don’t believe the experimental design can test “whether hypnotic suggestion can effectively modulate body image and/or body schema”. Response to these pointing and perceptual tasks might be taken to indicate relevant experience, but that such experience involves changes in “body image” or “body schema” is just one possibility, just as it is only one possibility among many that a subject’s regressed memories of life as Cleopatra are veridical. There are very good reasons to tread carefully here – the range of potential ‘discoveries’ which might arise from the application of reverse inference to response to imaginative suggestion is potentially vast.

Response : We appreciate the reviewer’s comments on the interpretation of behavioural measurement in our study. This is an important issue, which is actually widely spread in cognitive neuroscience: how to go from behaviours to what’s inside the back box? In the case of bodily processing, since Head and Holmes (1911), there has been a clinical tradition to take performance in perceptual or motor tasks as evidence in favour of a deficit of corresponding bodily representations (e.g. Paillard, 1999; Sirigu et al., 1991; Schwoebel and Coslett, 2005). It has further been found that performance in perceptual and motor tasks can be selectively modulated (e.g., Kammers et al., 2009; Gadsby, 2017). Finally, they correspond to distinct neural networks (Berlucchi and Aglioti, 2010; Naito et al., 2016; Ho and Lenggenhager, 2021). There may be other interpretations for all these results, but there is so far no evidence showing that these tasks do not measure two distinct types of bodily representations. Consequently, we believe that the body schema/body image hypothesis remains empirically and conceptually grounded. We have discussed extensively this hypothesis in other works (Vignemont, 2011; Vignemont, 2018; Vignemont et al., 2021) and we do not believe that our arguments should be repeated in this paper. 

We should also note that researchers routinely operationalize a term with a measure and then reference the term even though others might disagree (e.g., operationalizing phenomenological control as suggestibility and then interpreting the latter as the former). We also don’t think the analogies with past life regression or animal magnetism are relevant here just as we would never draw analogies between phenomenological control and animal magnetism.

Reviewer #2:

p.5 “Furthermore, demand characteristics – namely participant’s explicit

compliance, or unconscious changes in behaviour and experience in accordance, with implicit or explicit experimental aims (Orne, 1962) – were recently debated as a potential contributing factor to the RHI (Lush, 2020). Effects turned out to be weak and unsignificant when relevant controls were applied (Ehrsson et al., 2021).” p.19 “ (for the RHI, it was shown that with adequate control, the association was insignificant: Ehrsson et al., 2021).”

There is some confusion here. Lush (2020) showed that expectancies differ for the asynchronous control and synchronous illusion conditions of RHI studies. (see also replications in Reader 2022; Lush, Seth & Dienes, 2022). Asynchrony is not a valid control in the RHI, simply because control conditions must be matched for all important factors, and expectancies are clearly an important factor when controlling for demand characteristic effects. Ehrsson et al (2021) did not address the results of Lush (2020), but the results of Lush et al, (2020) which reported relationships between synchronous condition rubber hand illusion measures and hypnotisability, and also evidence for no relationship between illusion agreement scores and hypnotisability when ‘controlled’ by the asynchronous condition. Ehrsson et al reproduced this latter null result The interpretation of this null result rests on the validity of the control condition. Ehrsson et al assert that the control is valid, without evidence or argument. Similarly, in the manuscript, the control condition is described as ‘relevant’ and ‘adequate’, but no argument or reference for why it should be considered so has been provided. Please either provide an argument as to why this control procedure which is confounded by expectancies should be considered a valid control for demand characteristic effects or remove or revise this assertion. Rather than repeat our interpretation of this null result and related arguments here, I refer the authors to the arguments presented in our commentary published alongside Ehrsson et al (2021): Lush & Seth (2021) and in the original paper (Lush et al, 2020). In anticipation of a possible response, it is worth noting that a recent argument about the manipulation of expectancies in the original paper (deVignemont, 2022) is based on a misreading of our study design. It should also be noted that, even if the RHI control condition were valid, it would not provide good support for the claim that demand characteristics are not involved in the effects presented in this paper (in order to attribute the effects to changes in body schema etc). 

Response : We no longer think this debate is especially relevant to the current work and we have shortened or omitted discussion of these points. It should be noted that different expectancies in different conditions does not necessarily mean that an effect (condition difference) is driven by expectancies. For example, if we ask a group of athletes and a group of non-athletes their expectancies concerning their performance on a sportive task, we will likely have a group with high expectations and high scores and a group with low expectations and low scores. It would be a mistake to conclude that participants achieved higher scores because of their expectancies. We think this is an important point that has been lost in this discussion and we briefly highlight this in the “limitations of the study” section. Irrespective of control conditions in the RHI, we maintain that our sham control is adequate because it mimics all the aspects of the Lackner illusion procedure to the participant. Importantly, it doesn’t reliably elicit the illusion. We think studying expectancies with this paradigm is a useful suggestion and we have now made a recommendation regarding this in the Discussion. 

Reviewer #2:

P.20, section 4.2. The simple alternative theory that differences in effects between illusion and suggestion conditions may arise from differing demand characteristics is treated inconsistently in this section. While it is considered here as a possible explanation for order effects (as also may occur in the RHI, see Lush, 2021), it may also explain other results and should be consistently applied to conclusions. For example, the relatively clear demand characteristics in the hypnotic suggestion condition may explain the correlation between measures in the suggestion condition in contrast with the illusion condition (though please ensure all claims of null results are backed by analyses other than non-significant p values - see below).

Response : An argument about why we think that the increased correlation is not a result of demand characteristics has been added to the manuscript (pp.22-23) “First, if the effect of the hypnotic suggestion were caused by compliance in response to demand characteristics, social desirability would be expected to correlate to some extent with effects in the hypnotic condition, which is against the evidence. Second, this interpretation predicts that pointing effect follows perceptual effect. Indeed, in this view participants might shift their perceptual reports in response to demand characteristic but correctly follow instructions in the pointing task. However, the opposite does not hold, as participants going as far as shifting their pointing in line with the suggestion should report at least similar elongation in the perceptual task. One consequence is that the distribution of differences between perceptual and pointing effects should be shifted towards positive values, with negative values only corresponding to pointing inaccuracies. However, the data suggest that it is not the case, with the discrepancy between perceptual and pointing effect being reasonably close to a gaussian distribution centred on zero.”

Reviewer #2:

A further related issue - the indirect measures bear surface similarity to proprioceptive drift in the RHI (which is confounded by demand characteristics; Lush, Seth & Dienes, 2021) and may have similar problems.

As noted above, a demonstration of different expectancies does not mean something is confounded only that it might be confounded. To convincingly demonstrate this, it will be important to modulate expectancies. Sometimes expectancy claims are overstated, and we don’t want to make that mistake here. We have noted that further interrogation of features of the principal features of this illusion and procedure are warranted. 

“However, in contrast with claims made by some of these authors, a correlation between hypnotic suggestibility and a bodily illusion does not necessarily entail that an illusion is driven by suggestion and expectations.”

Please clearly state which of the papers contain this claim. Without direct reference, it is a strawman (I don’t recall seeing this argument in any of the cited papers, but I could well have missed it). As an aside, regarding the plausibility of suggestion effects in the rubber hand illusion, I think it is a mistake to consider this one illusion in isolation – similar relationships have been shown for mirror touch synaesthesia pain, ASMR and visually evoked auditory response. While other mechanisms may be in play in each case, that these relationships are due to imaginative suggestion effects arising from demand characteristics presents a simple, testable theory.

Response : We fully agree that this is an interesting hypothesis that warrants further attention. However, strong claims along these lines have been made in multiple papers:

“First, as a way of more rigorously exploring phenomena which phenomenological control can surreptitiously bring about without subject nor experimenter necessarily realizing.” Lush et al. 2021 (https://psyarxiv.com/zhgeb/)

Correlations between these phenomena and suggestibility only show that they’re correlated not that suggestion can bring about these effects.

“If the effect is entirely produced by phenomenological control the slope should be steep and the intercept close to 0. … Indeed, the extent to which people experience vEAR is strongly related to their score on the Phenomenological Control Scale (with zero intercept […]”. (Dienes & Lush, 2022 “The role of phenomenological control in experience”).

Insofar as two different variables could correlate with a third variable and both effects could have zero intercepts, this doesn’t hold. Either way, note the use of “entirely produced” when referring to a correlation. 

“That is, the sensations of ASMR may be largely constructed based on a belief of the situations that should bring them about." (Dienes & Lush, 2023).

Again, these are correlational data.

“Evidence that hypnotisability scores predict the most common RHI measures would be consistent with the theory that the RHI, as commonly reported, is at least partially driven by phenomenological control.” (Lush et al. 2020 pp.4)

“Our results show that RHI reports are, at least partially, likely to be driven by top-down phenomenological control in response to demand characteristics” (Lush & Seth, 2022 pp.1)

“Rubber hand illusion report is confounded by demand characteristics and may entirely reflect compliance, bias and implicit imaginative suggestion effects“

This is the title of the original paper. 

"Mirror-touch synesthesia occurs when a person feels touch that they see someone else receive. Vicarious pain occurs when a person feels pain when they see someone else receiving noxious stimulation. Both effects depend steeply on hypnotizability, with small though not necessarily zero intercepts, when tested in unrelated contexts (Lush et al., 2020)."

Note the word "depend", which denotes a causal relationship.

Reviewer #2:

P.22. “ In this framework, the hypnotic suggestions did not directly alter body representations; rather, participants changed their behaviour and experience according to their expectations...”

This paragraph is a good summary which matches my interpretation of the results. I don’t see anything in these data which is not consistent with this position. While it is only one interpretation, that it can be applied to the data presented without issue highlights that the conclusions regarding body schema etc are an overstatement of the presented results.

Response : Problems with this interpretation have been added to the manuscript (pp.22-23) “First, if the effect of the hypnotic suggestion were caused by compliance in response to demand characteristics, social desirability would be expected to correlate to some extent with effects in the hypnotic condition, which is against the evidence. Second, this interpretation predicts that pointing effect follows perceptual effect. Indeed, in this view participants might shift their perceptual reports in response to demand characteristic but correctly follow instructions in the pointing task. However, the opposite does not hold, as participants going as far as shifting their pointing in line with the suggestion should report at least similar elongation in the perceptual task. One consequence is that the distribution of differences between perceptual and pointing effects should be shifted towards positive values, with negative values only corresponding to pointing inaccuracies. However, the data suggest that it is not the case, with the discrepancy between perceptual and pointing effect being reasonably close to a gaussian distribution centred on zero.”

Reviewer #2:

Other points

As far as I can tell, it seems that one of the two measures of the Lackner illusion employed here may say survive sham control. This, to me, was among the more interesting aspects of the present manuscript. I think it would be worth bringing out more.

We thank the reviewer for his interest in that part. The effect of the Lackner illusion on the perceived size of the finger is only a replication of previous studies (Lackner 1988, Burrack & Brugger 2005, Vignemont et al. 2005, Ehrsson et al. 2005) and therefore it is not very surprising. Since it is quite peripheral to our main question, we prefer not commenting more on that result.

P.8 “By comparing the reported finger size at rest to the actual finger size, we can derive an estimate of the validity of the measure.” I think I get the intended meaning, but the use of “validity” here is inadvisable given the specific meanings of the term in psychological science.

Response : It is true that validity here is a misnomer, and we changed it: “…we can derive an estimate of the bias in the measurement” (pp.8)

Reviewer #2:

P.23 “supported by imagery and selective attention”

It might be worth noting evidence against trait differences in attention in response to imaginative suggestion here (see Martin & Pacherie, 2018, for example).

Response : Thank you for the suggestion. It is true that there is no clear relationship between measures of attention and hypnotic suggestibility, and it deserves to be mentioned here. “…the suggested prior could be supported by imagery and selective attention to seemingly confirming evidence while downplaying disconfirming evidence, though the relationship of these variable with response to suggestion is probably complex and debated (on imagery e.g., [96,97]; on attention e.g., [98–101] ).” (pp.23-24)

Reviewer #2:

P.24 “We did not index response expectancies because doing so may provide participants with explicit indicators of how they are expected to respond”

These could be checked in a separate sample, e.g., to address (possibly) differing results between hypnosis and illusion conditions (I’m not suggesting you should do this now, btw).

Response : Agreed, this might be an experiment we will do in the future.

Reviewer #2:

Results are sometimes reported in cm and sometimes in mm. Better to choose one or the other throughout.

Response : Indeed, this has been changed so that distances are in mm throughout, thank you for the suggestion.

Reviewer #2:

Open data – the variables in the provided file are difficult to interpret without a key. For example, what do the ‘pinocchio’ columns refer to?

Response : The OSF file has been updated to be interpreted more easily. Header names referred to older version of the manuscript where the “Lackner illusion” was referred to as “Pinocchio illusion” as it is sometimes used in the community.

Attachment Submitted filename: Response to Reviewers.pdf Click here for additional data file.

# Peer review: Decision Letter 1

**DOI:** 10.1371/journal.pone.0291493.r003

**Authors:** Jane Elizabeth Aspell

18 Apr 2023

PONE-D-22-33125R1Hypnotic suggestion versus sensory modulation of bodily awarenessPLOS ONE

Dear Dr. Apelian,

Thank you for submitting your manuscript to PLOS ONE. After careful consideration, we feel that it has merit but does not fully meet PLOS ONE’s publication criteria as it currently stands. Reviewer 2 still has a number of significant concerns that need to be addressed. Therefore, we invite you to submit a revised version of the manuscript that addresses the points raised during the review process.

Please submit your revised manuscript by Jun 02 2023 11:59PM. If you will need more time than this to complete your revisions, please reply to this message or contact the journal office at plosone@plos.org. When you're ready to submit your revision, log on to https://www.editorialmanager.com/pone/ and select the 'Submissions Needing Revision' folder to locate your manuscript file.

Please include the following items when submitting your revised manuscript:

- A rebuttal letter that responds to each point raised by the academic editor and reviewer(s). You should upload this letter as a separate file labeled 'Response to Reviewers'.
- A marked-up copy of your manuscript that highlights changes made to the original version. You should upload this as a separate file labeled 'Revised Manuscript with Track Changes'.
- An unmarked version of your revised paper without tracked changes. You should upload this as a separate file labeled 'Manuscript'.

If you would like to make changes to your financial disclosure, please include your updated statement in your cover letter. Guidelines for resubmitting your figure files are available below the reviewer comments at the end of this letter.

If applicable, we recommend that you deposit your laboratory protocols in protocols.io to enhance the reproducibility of your results. Protocols.io assigns your protocol its own identifier (DOI) so that it can be cited independently in the future. For instructions see: https://journals.plos.org/plosone/s/submission-guidelines#loc-laboratory-protocols. Additionally, PLOS ONE offers an option for publishing peer-reviewed Lab Protocol articles, which describe protocols hosted on protocols.io. Read more information on sharing protocols at https://plos.org/protocols?utm_medium=editorial-email&utm_source=authorletters&utm_campaign=protocols.

We look forward to receiving your revised manuscript.

Kind regards,

Jane Elizabeth Aspell, PhD

Academic Editor

PLOS ONE

[Note: HTML markup is below. Please do not edit.]

Reviewers' comments:

Reviewer's Responses to Questions

**Comments to the Author**

1. If the authors have adequately addressed your comments raised in a previous round of review and you feel that this manuscript is now acceptable for publication, you may indicate that here to bypass the “Comments to the Author” section, enter your conflict of interest statement in the “Confidential to Editor” section, and submit your "Accept" recommendation.

Reviewer #1: All comments have been addressed

Reviewer #2: (No Response)

**********

2. Is the manuscript technically sound, and do the data support the conclusions?

The manuscript must describe a technically sound piece of scientific research with data that supports the conclusions. Experiments must have been conducted rigorously, with appropriate controls, replication, and sample sizes. The conclusions must be drawn appropriately based on the data presented. 

Reviewer #1: Yes

Reviewer #2: No

**********

3. Has the statistical analysis been performed appropriately and rigorously? 

Reviewer #1: Yes

Reviewer #2: No

**********

4. Have the authors made all data underlying the findings in their manuscript fully available?

The PLOS Data policy requires authors to make all data underlying the findings described in their manuscript fully available without restriction, with rare exception (please refer to the Data Availability Statement in the manuscript PDF file). The data should be provided as part of the manuscript or its supporting information, or deposited to a public repository. For example, in addition to summary statistics, the data points behind means, medians and variance measures should be available. If there are restrictions on publicly sharing data—e.g. participant privacy or use of data from a third party—those must be specified.

Reviewer #1: Yes

Reviewer #2: Yes

**********

5. Is the manuscript presented in an intelligible fashion and written in standard English?

PLOS ONE does not copyedit accepted manuscripts, so the language in submitted articles must be clear, correct, and unambiguous. Any typographical or grammatical errors should be corrected at revision, so please note any specific errors here.

Reviewer #1: Yes

Reviewer #2: Yes

**********

6. Review Comments to the Author

Please use the space provided to explain your answers to the questions above. You may also include additional comments for the author, including concerns about dual publication, research ethics, or publication ethics. (Please upload your review as an attachment if it exceeds 20,000 characters)

Reviewer #1: The manuscript has been effectively revised in response to the points raised. The authors should be commended for undertaking a thorough response to the review.

Reviewer #2: This manuscript presents data for a finger length perception task and a line reaching task in three conditions: sham control, Lackner illusion and a hypnotic suggestion for finger elongation (which is comparable to a classic hypnosis scale item for an experience of the hands being drawn together by an imaginary magnetic force). There is evidence for a Lackner effect larger than the effect in the sham condition, but interpretation of this difference is complicated by a lack of evidence that a confounding variable is matched across conditions). The authors appeal to differences in the relationships between trait response to hypnotic imaginative suggestion (hypnotisability) and perception measure response between the hypnotic suggestion and Lackner conditions to support arguments about mechanism. However, there is no evidence for or against a difference in these relationships. In my view, the simplest explanation for the difference between the Lackner illusion and hypnotic suggestion conditions is a difference in expectancies between the two conditions. The hypothesis-consistent demand characteristics seem to me likely to be clearer for the hypnotic suggestion than for the Lackner condition (because in the first case they are explicit and in the latter case implicit).

I did not review the results in detail in the first version of the manuscript. The results have now been extensively revised, and I will therefore focus this review the results and related claims in the discussion. Many of the claims in the discussion are based on invalid inferences, which I will detail below. I will focus on the perception measure because for the line-reaching measure there was no evidence for or against an effect in the Lackner condition, or evidence for a difference between sham control and Lackner condition. I will not review in detail any discussion which is based on invalid statistical inference.

I recommend major revision to address the problems I discuss below, including: remove invalid inferences and related discussion, to correct the definition of cold control theory and to clarify the definition of body schema employed in both the introduction and abstract.

1) Response : We agree with the reviewer that a non-significant p-value should not be interpreted as evidence supporting the null hypothesis. It is also true that using Bayes Factor in several instances rather than p-values allows for a stronger conclusions, and we thank the reviewer for this suggestion. On the other hand, we do think it is not inaccurate to state “did not significantly correlate” – please note that this is not a null claim (e.g., “did not correlate”) – only a claim that a significant effect was not observed. We do acknowledge that in a few cases we incorrectly omitted the word “significant” and we have made sure to include that as appropriate throughout. This is especially appropriate when we’re not interested in the null per se but only that a covariate did not significantly confound our results. We have now added Bayes factors for all analyses (see Table 1, Table 2, Table 3, Table 4 and Fig 4). We have now clearly stated that we are using default Bayes factors throughout.

I thank the authors for removing some invalid claims based on non-significant p values (e.g., that a non-significant result is surprising). However, there are many such claims in the discussion section of the revised manuscript. For example, claims of differences for significant and non-significant correlations between hypnotisability and either the Lackner illusion or hypnotic suggestion conditions. The only evidence for or against a relationship in these cases is a p value below .05 for a positive relationship between hypnotic suggestion response and hypnotisability.

p.19: “Weaker and non-significant correlations with hypnotic

suggestibility were observed for perceptual effect in the Lackner illusion and sham condition.”

p.19 “Our data do not exclude the possibility that hypnotic suggestibility accounts for variability in the Lackner illusion,although our sample size was not large enough to detect this association. Nevertheless, our results suggest that if this association were present, it would be rather weak.”

p.19 “This sensitivity to alterations of bodily experience across bottom-up

and top-down contexts is probably not reducible to hypnotic suggestibility since correlations with effects in the Lackner illusion condition were weak and unlikely to differ from zero.”

“Relatedly, the specific effect of the Lackner illusion cannot be explained by top-down processing since it survived the inclusion of hypnotic suggestibility as a covariate in our model of perceptual effect.”

Furthermore, to support claims of a difference between the correlations, a test of the difference is required. I calculated confidence intervals from the online data (the manuscript is lacking CIs and SEs). The (bootstapped) 95% CI for the Pearson coefficient between hypnotisability and Lackner illusion is [.02, .48 ] and for the relationship between hypnotisability and response to suggestion [.08, .51]. The CI for the hypnotisability/Lackner illusion coefficient contains the point value of the parameter estimate for the hypnotisability/suggestion coefficient. The data are simply too noisy to distinguish between these relationships. A larger sample would be required.

There is no statistical evidence for or against a line reaching/pointing effect in the Lackner condition to support the following claims:

p.20 “Although the Lackner illusion did modify body image, we found that it did not significantly modify body schema. This dissociation aligns with similar results showing that action can be immune to bodily illusions in some circumstances”

p.21 “Body image and body schema can be sometimes dissociated, thus motivating the conceptual

distinction between the two bodily representations [51]. This seems to be confirmed by the dissociation between the effect of the Lackner illusion on body image, and the lack of effect on body schema.”

19. Furthermore, the stimulation was apparently similar between the sham and the Lackner illusion procedure and the instructions were identical. The sham condition was thus able to provide a good control condition for confounding expectancies, as suggested by similar correlations with hypnotic suggestibility.

No justification for models of H1 has been included. This issue could be somewhat mitigated If robustness regions are reported (so that readers can see the range of models consistent with the inference). Bayes factors are only informative regarding the relative evidence for two theories. If the model of H1 is not reasonable, the Bayes factor is not useful, but the authors provide no argument as to why the chosen model of H1 is considered reasonable. There are earlier studies of the Lackner illusion which could inform the choice of model.

Models of H1 have only been provided for post-hoc comparisons, and not for other analyses (e.g., correlational analyses).

No criterion is provided for the interpretation of Bayes factors.

2) Response : We appreciate the reviewer’s comments on the interpretation of behavioural measurement in our study. This is an important issue, which is actually widely spread in cognitive neuroscience: how to go from behaviours to what’s inside the back box? In the case of bodily processing, since Head and Holmes (1911), there has been a clinical tradition to take performance in perceptual or motor tasks as evidence in favour of a deficit of corresponding bodily representations (e.g. Paillard, 1999; Sirigu et al., 1991; Schwoebel and Coslett, 2005). It has further been found that performance in perceptual and motor tasks can be selectively modulated (e.g., Kammers et al., 2009; Gadsby, 2017). Finally, they correspond to distinct neural networks (Berlucchi and Aglioti, 2010; Naito et al., 2016; Ho and Lenggenhager, 2021). There may be other interpretations for all these results, but there is so far no evidence showing that these tasks do not measure two distinct types of bodily representations. Consequently, we believe that the body schema/body image hypothesis remains empirically and conceptually grounded. We have discussed extensively this hypothesis in other works (Vignemont, 2011; Vignemont, 2018; Vignemont et al., 2021) and we do not believe that our arguments should be repeated in this paper.

I presume that in each of these other cases the experimental effects have been at least assumed to not be suggestion effects. That's not possible for a direct suggestion.

As a side note, I should clarify my comments on a body schema interpretation of the suggestion condition in my previous review. I was not saying you shouldn’t be free to make the claim that hypnotic suggestion changes body schema, but rather noting some responses I think this claim is likely to face from imaginative suggestion researchers.

3) We should also note that researchers routinely operationalize a term with a measure and then reference the term even though others might disagree (e.g., operationalizing phenomenological control as suggestibility and then interpreting the latter as the former). We also don’t think the analogies with past life regression or animal magnetism are relevant here just as we would never draw analogies between phenomenological control and animal magnetism.

This paragraph is not directly relevant to my review of this manuscript, but note that “phenomenological control is not “operationalized as suggestibility””. It is a label we use to refer to hypnotic suggestibility (and response to implicit or direct suggestion in contexts other than hypnosis) with the aim of avoiding misunderstandings arising from other terms.

E.g., Dienes et al (2022) https://psycnet.apa.org/doi/10.1037/cns0000230: “Phenomenological control is the ability to control subjective experience so that a constructed counterfactual state of affairs appears real. It is not a new concept. We as a community have been calling it hypnotizability (in the context of hypnotic inductions) or suggestibility. But “hypnosis” refers to sleep, which is unrelated to phenomenological control (e.g. Banyai & Hilgard, 1976); and “suggestibility” implies being manipulated contrary to one’s real intentions, a gullibility that is little related to hypnotic response (e.g. Coe, Kobayashi, & Howard, 1973; Moore, 1964). So in our laboratory, we have begun to refer to hypnotic suggestibility in a more general context as the capacity for phenomenological control and, correspondingly, hypnotic response as an example of phenomenological control.”

I do not consider reference to mesmerism or hypnotic suggestion effects to be irrelevant or inappropriate when discussing an experimental investigation of response to hypnotic suggestion. Neither my colleagues nor I would be offended by comparisons between phenomenological control and mesmerism. Phenomenological control is an explanation of mesmerism. We often discuss mesmerism alongside phenomenological control.

4) We no longer think this debate is especially relevant to the current work

and we have shortened or omitted discussion of these points. It should be noted that

different expectancies in different conditions does not necessarily mean that an effect

(condition difference) is driven by expectancies…

We do not anywhere claim that expectancy studies show an effect is necessarily attributable to demand characteristics. I stated our argument in my first review: “Asynchrony is not a valid control in the RHI, simply because control conditions must be matched for all important factors, and expectancies are clearly an important factor when controlling for demand characteristic effects.”

An experiment which employs controls for a known extraneous variable which is unmatched on that extraneous variable cannot rule out confounding effects of the extraneous variable. This argument is neither controversial nor our invention. The APA dictionary of psychology gives the following definition of control:

“ the regulation of all extraneous conditions and variables in an experiment so that any change in the dependent variable can be attributed solely to manipulation of the independent variable and not to any other factors.”

5) Irrespective of control conditions in the RHI, we maintain that our sham control is adequate because it mimics all the aspects of the Lackner illusion procedure to the participant. Importantly, it doesn’t reliably elicit the illusion. We think studying expectancies with this paradigm is a useful suggestion and we have now made a recommendation regarding this in the Discussion.

That a control condition doesn’t produce the same responses as the experimental condition is not evidence for its validity. The sham control here may well be a good match for expectancies. Or it may not. Please remove any assertions that expectancies were similar across sham and lackner conditions. E.g., that the stimulus was “apparently similar” across conditions.

6) “First, if the effect of the hypnotic suggestion were caused by compliance in response to

Demand characteristics, social desirability would be expected to correlate to some

extent with effects in the hypnotic condition, which is against the evidence.

Null relationships between response to an imaginative suggestion and social desirability scales do not provide evidence against cold control theory. Social desirability is not a good predictor of hypnotic responding. The claim in cold control theory is that response to imaginative suggestion is not reducible to social desirability.

E.g., Lush et al (2020) https://doi.org/10.1038/s41467-020-18591-6:

“Hypnotic responding is distinct from social compliance or social desirability, and is not attributable to mere compliance.”

On a related note, cold control theory is described inaccurately throughout the manuscript (leading to some confusion in arguments). Cold control is a theory of hypnosis. Unless the authors define hypnosis as compliance (which they do not), this description doesn’t make sense. It is inconsistent to describe cold control as compliance and other theories as not reducible to compliance.

For example, in the final paragraph, cold control is described as “compliance without awareness”. An accurate description of cold control here would be: “intentional imagination without awareness of intention”.

7) Second,this interpretation predicts that pointing effect follows perceptual effect. Indeed, in this view participants might shift their perceptual reports in response to demand characteristic but correctly follow instructions in the pointing task. However, the opposite does not hold, as participants going as far as shifting their pointing in line with the suggestion should report at least similar elongation in the perceptual task. One consequence is that the distribution of differences between perceptual and pointing effects should be shifted towards positive values, with negative values only corresponding to pointing inaccuracies. However, the data suggest that it is not the case, with the discrepancy between perceptual and pointing effect being reasonably close to a gaussian distribution centred on zero.”

This argument is not supported by any analyses. That aside, a simple explanation for any difference between the two tasks is differing expectancies, but given there was no evidence for an effect of the Lackner illusion on this measure, it may well have other issues.

8) Response : We fully agree that this is an interesting hypothesis that warrants further attention. However, strong claims along these lines have been made in multiple papers…

The authors list quotes from studies in which we describe testing a causal theory with correlational evidence to support their claim that we infer causation from correlation. Testing a causal theory with correlational evidence is not the same as inferring causation from correlation.

9) As far as I can tell, it seems that one of the two measures of the Lackner illusion employed here may say survive sham control. This, to me, was among the more interesting aspects of the present manuscript. I think it would be worth bringing out more. We thank the reviewer for his interest in that part. The effect of the Lackner illusion on the perceived size of the finger is only a replication of previous studies (Lackner 1988, Burrack & Brugger 2005, Vignemont et al. 2005, Ehrsson et al. 2005) and therefore it is not very surprising. Since it is quite peripheral to our main question, we prefer not commenting more on that result.

My intended point was that this could provide evidence that there is a Lackner illusion effect which might not be attributable to demand characteristics. For this to hold, expectancies for the Lackner and sham conditions would need to be shown to match on expectancies. I don’t know how common sham controls are in Lackner illusion studies, but if this is not already well established it may be worth testing.

10) “the link between the two body representations and behavioural measures is definitional: body image is the body you perceive and thus variations in the perceptual task indexes changes in the body image.”

This clarifies for me the arguments regarding hypnotic suggestion changing body schema. These arguments are straightforward if reports of changing body image are definitionally changes in body schema. However body schema are now not an explanation but a description. If body schema is simply a label applied to reports about body image, the inconsistency in the discussion section is resolved:

P.18 “These results demonstrate that hypnotic suggestion is an effective technique for transient modulations of body image and body schema.”

And

P. 26 “The claim, however, is not that hypnotic suggestion engages the same basic perceptual processes as Lackner illusion. Instead, we discussed two interpretations of the effect, either low-level in terms of cognitive penetration, or high-level in terms of compliance without awareness.”

The authors’ definition of “body schema” should be established in the introduction, to reduce the risk of confusing readers who may have a different definition in mind (as I did).

11) As the study is not pre-registered, please clarify the exploratory nature of analyses.

Please state inferential rules followed in the analysis section. E.g., criteria for BF sensitivity (conventionally 3 and 1/3 or 6 and 1/6) and which inferences are based on which statistical approach (it seems that p values are interpreted for effects and Bayes factors for null effects, but sometimes this is not the case).

**********

7. PLOS authors have the option to publish the peer review history of their article (what does this mean?). If published, this will include your full peer review and any attached files.

If you choose “no”, your identity will remain anonymous but your review may still be made public.

**Do you want your identity to be public for this peer review?** For information about this choice, including consent withdrawal, please see our Privacy Policy.

Reviewer #1: No

Reviewer #2: **Yes: **Peter Lush

**********

[NOTE: If reviewer comments were submitted as an attachment file, they will be attached to this email and accessible via the submission site. Please log into your account, locate the manuscript record, and check for the action link "View Attachments". If this link does not appear, there are no attachment files.]

While revising your submission, please upload your figure files to the Preflight Analysis and Conversion Engine (PACE) digital diagnostic tool, https://pacev2.apexcovantage.com/. PACE helps ensure that figures meet PLOS requirements. To use PACE, you must first register as a user. Registration is free. Then, login and navigate to the UPLOAD tab, where you will find detailed instructions on how to use the tool. If you encounter any issues or have any questions when using PACE, please email PLOS at figures@plos.org. Please note that Supporting Information files do not need this step.

Attachment Submitted filename: Lackner RHI review.pdf Click here for additional data file.

# Author comment: Author response to Decision Letter 1

**DOI:** 10.1371/journal.pone.0291493.r004

13 Jun 2023

Formated response can be found in the document "Response to reviewers"

Attachment Submitted filename: Response to reviewers.docx Click here for additional data file.

# Peer review: Decision Letter 2

**DOI:** 10.1371/journal.pone.0291493.r005

**Authors:** Jane Elizabeth Aspell

9 Jul 2023

PONE-D-22-33125R2Hypnotic suggestion versus sensory modulation of bodily awarenessPLOS ONE

Dear Dr. Apelian,

Thank you for submitting your manuscript to PLOS ONE. While one reviewer has recommended accepting the manuscript the other has some remaining recommendations for revision which need to be addressed in full. Therefore, we invite you to submit a revised version of the manuscript that addresses the points raised during the review process.

Please submit your revised manuscript by Aug 23 2023 11:59PM. If you will need more time than this to complete your revisions, please reply to this message or contact the journal office at plosone@plos.org. When you're ready to submit your revision, log on to https://www.editorialmanager.com/pone/ and select the 'Submissions Needing Revision' folder to locate your manuscript file.

Please include the following items when submitting your revised manuscript:

- A rebuttal letter that responds to each point raised by the academic editor and reviewer(s). You should upload this letter as a separate file labeled 'Response to Reviewers'.
- A marked-up copy of your manuscript that highlights changes made to the original version. You should upload this as a separate file labeled 'Revised Manuscript with Track Changes'.
- An unmarked version of your revised paper without tracked changes. You should upload this as a separate file labeled 'Manuscript'.

If you would like to make changes to your financial disclosure, please include your updated statement in your cover letter. Guidelines for resubmitting your figure files are available below the reviewer comments at the end of this letter.

If applicable, we recommend that you deposit your laboratory protocols in protocols.io to enhance the reproducibility of your results. Protocols.io assigns your protocol its own identifier (DOI) so that it can be cited independently in the future. For instructions see: https://journals.plos.org/plosone/s/submission-guidelines#loc-laboratory-protocols. Additionally, PLOS ONE offers an option for publishing peer-reviewed Lab Protocol articles, which describe protocols hosted on protocols.io. Read more information on sharing protocols at https://plos.org/protocols?utm_medium=editorial-email&utm_source=authorletters&utm_campaign=protocols.

We look forward to receiving your revised manuscript.

Kind regards,

Jane Elizabeth Aspell, PhD

Academic Editor

PLOS ONE

Journal Requirements:

Please review your reference list to ensure that it is complete and correct. If you have cited papers that have been retracted, please include the rationale for doing so in the manuscript text, or remove these references and replace them with relevant current references. Any changes to the reference list should be mentioned in the rebuttal letter that accompanies your revised manuscript. If you need to cite a retracted article, indicate the article’s retracted status in the References list and also include a citation and full reference for the retraction notice.

[Note: HTML markup is below. Please do not edit.]

Reviewers' comments:

Reviewer's Responses to Questions

**Comments to the Author**

1. If the authors have adequately addressed your comments raised in a previous round of review and you feel that this manuscript is now acceptable for publication, you may indicate that here to bypass the “Comments to the Author” section, enter your conflict of interest statement in the “Confidential to Editor” section, and submit your "Accept" recommendation.

Reviewer #1: All comments have been addressed

Reviewer #2: (No Response)

**********

2. Is the manuscript technically sound, and do the data support the conclusions?

The manuscript must describe a technically sound piece of scientific research with data that supports the conclusions. Experiments must have been conducted rigorously, with appropriate controls, replication, and sample sizes. The conclusions must be drawn appropriately based on the data presented. 

Reviewer #1: Yes

Reviewer #2: Partly

**********

3. Has the statistical analysis been performed appropriately and rigorously? 

Reviewer #1: Yes

Reviewer #2: No

**********

4. Have the authors made all data underlying the findings in their manuscript fully available?

The PLOS Data policy requires authors to make all data underlying the findings described in their manuscript fully available without restriction, with rare exception (please refer to the Data Availability Statement in the manuscript PDF file). The data should be provided as part of the manuscript or its supporting information, or deposited to a public repository. For example, in addition to summary statistics, the data points behind means, medians and variance measures should be available. If there are restrictions on publicly sharing data—e.g. participant privacy or use of data from a third party—those must be specified.

Reviewer #1: Yes

Reviewer #2: (No Response)

**********

5. Is the manuscript presented in an intelligible fashion and written in standard English?

PLOS ONE does not copyedit accepted manuscripts, so the language in submitted articles must be clear, correct, and unambiguous. Any typographical or grammatical errors should be corrected at revision, so please note any specific errors here.

Reviewer #1: Yes

Reviewer #2: Yes

**********

6. Review Comments to the Author

Please use the space provided to explain your answers to the questions above. You may also include additional comments for the author, including concerns about dual publication, research ethics, or publication ethics. (Please upload your review as an attachment if it exceeds 20,000 characters)

Reviewer #1: The manuscript has been effectively revised in response to the points raised. The authors should be commended for undertaking a thorough response to the review.

Reviewer #2: This revised manuscript presents evidence for positive relationships between measures of trait hypnotisability and measures intended to reflect experience of an elongated finger for the Lackner illusion and a hypnotic suggestion for that experience. A null relationship between response and a measure of trait social compliance is also presented. The effect is stronger for the suggestion condition than for the Lackner condition. Because the sham control is not matched for demand characteristic effects with the Lackner condition, it is uninformative as to whether or not the Lackner illusion can be attributed to demand characteristics. A simple interpretation of these results is that both the Lackner illusion and the hypnotic suggestion are suggestion effects arising from phenomenological control. My interpretation of the difference in magnitude of effect between these conditions is as follows. In the hypnotic suggestion condition, demand characteristics will be relatively clear (because the participants are told what they should experience). In the Lackner condition, participants must work out what they are expected to experience themselves. The difference in effect between condition may reflect a resulting difference in the consistency of participant expectancies across conditions.

I thank the authors for addressing the issues I raised in the last review. However, there remain a couple of major inaccuracies which prevent the conclusions from following from both the presented data and existing evidence. There is also important information missing for Bayesian analyses. I will detail these below and provide some other brief comments which I hope will be useful.

1) On p.21 “If the effect of the hypnotic suggestion were driven by compliance in response to demand

characteristics, social desirability would be expected to correlate with responses,”. This is incorrect. First, null relationships with social desirability measures do not provide evidence against demand characteristic effects (see e.g., Nichols & Maner, 2008). Second, the idea that response to imaginative suggestion is attributable to being controlled by a ‘hypnotist’ or being gullible or weak willed etc is a myth arising from the hypnotic context (e.g., see (Lynn et al., 2020.). As I stated in the last round of review, we employ the term ‘phenomenological control’ with the aim of avoiding misunderstandings of this kind which can arise from use of the term ‘suggestibility’. Response to imaginative suggestion is goal-directed and strategic. A null relationship between measures of social compliance and a given effect does not provide evidence against a cold control mechanism for that effect. These interpretations of social desirability results don’t hold and should be revised or removed.

2) It is asserted that there was “no systematic expectation” (p.19) for the Lackner illusion condition. I do not consider the claim on p.12 to be sufficient evidence against expectancy differences to support this assertion (“No participant reported being aware that one of the conditions was a sham.”). Whether there is “systematic expectation” for the Lackner condition is an open question. I think it is likely that there is. My position is informed by evidence for hypothesis awareness in a measure similar to the pointing task here in another embodiment illusion procedure (“proprioceptive drift”): https://royalsocietypublishing.org/doi/10.1098/rsos.210911 . In any case, there is no evidence provided here to decide this matter (and the claim is contradicted in subsequent paragraphs). This assertion should be removed.

3) “Robustness tests with a wider prior (a width of √2,i.e., twice the size of the original prior) reached similar conclusions and thus the report results do not seem to be highly dependent on the specific parameters chosen for the prior.”

As requested in the previous round, robustness regions should be reported (alongside each Bayes factor). The sentence above is not a sufficient alternative. Robustness regions allow the reader to interpret Bayes factors according to their own model of H1. Because the analysis employs default models of H1, which are uniformed by evidence, RRs are of particular importance.

Other points:

• P.14, Ln 11: “By contrast”. Although this might be intended to only refer to a contrast in reference to sensitivity of Bayesian analysis, it could also be taken to mean a sensitive result contrasts meaningfully with an insensitive result. Because an insensitive result cannot be contrasted in this way (it provides no evidence for either H1 or H0 and is therefore uninformative), this second interpretation would not be valid. For this reason I recommend removing any reference to insensitive Bayes factors contrasting with sensitive Bayes factors.

• P.21 “When the suggested expectancies are unlikely for a participant, as might be expected for a low suggestible participant”

• This claim lacks evidence. The relationship between expectancies and hypnotic responding does not indicate that low hypnotisables have particularly low expectancies (e.g., Benham et al, 2006).

Lynn, S. J., Kirsch, I., Terhune, D. B., & Green, J. P. (n.d.). Myths and Misconceptions About Hypnosis and Suggestion: Separating Fact and Fiction. Applied Cognitive Psychology, 2020. https://doi.org/10.1002/acp.3730

Nichols, A. L., & Maner, J. K. (2008). The Good-Subject Effect: Investigating Participant Demand Characteristics. The Journal of General Psychology, 135(2), 151–166. https://doi.org/10.3200/GENP.135.2.151-166

**********

7. PLOS authors have the option to publish the peer review history of their article (what does this mean?). If published, this will include your full peer review and any attached files.

If you choose “no”, your identity will remain anonymous but your review may still be made public.

**Do you want your identity to be public for this peer review?** For information about this choice, including consent withdrawal, please see our Privacy Policy.

Reviewer #1: No

Reviewer #2: **Yes: **Peter Lush

**********

[NOTE: If reviewer comments were submitted as an attachment file, they will be attached to this email and accessible via the submission site. Please log into your account, locate the manuscript record, and check for the action link "View Attachments". If this link does not appear, there are no attachment files.]

While revising your submission, please upload your figure files to the Preflight Analysis and Conversion Engine (PACE) digital diagnostic tool, https://pacev2.apexcovantage.com/. PACE helps ensure that figures meet PLOS requirements. To use PACE, you must first register as a user. Registration is free. Then, login and navigate to the UPLOAD tab, where you will find detailed instructions on how to use the tool. If you encounter any issues or have any questions when using PACE, please email PLOS at figures@plos.org. Please note that Supporting Information files do not need this step.

# Author comment: Author response to Decision Letter 2

**DOI:** 10.1371/journal.pone.0291493.r006

24 Jul 2023

Response to Reviewers [see the attached "response to reviewers" file for colors]

We thank the second reviewer for his additional comments. Our responses (in blue) are below, and we have also highlighted corresponding changes in the text.

Reviewer #2:

This revised manuscript presents evidence for positive relationships between measures of trait hypnotisability and measures intended to reflect experience of an elongated finger for the Lackner illusion and a hypnotic suggestion for that experience. A null relationship between response and a measure of trait social compliance is also presented. The effect is stronger for the suggestion condition than for the Lackner condition. Because the sham control is not matched for demand characteristic effects with the Lackner condition, it is uninformative as to whether or not the Lackner illusion can be attributed to demand characteristics. A simple interpretation of these results is that both the Lackner illusion and the hypnotic suggestion are suggestion effects arising from phenomenological control. My interpretation of the difference in magnitude of effect between these conditions is as follows. In the hypnotic suggestion condition, demand characteristics will be relatively clear (because the participants are told what they should experience). In the Lackner condition, participants must work out what they are expected to experience themselves. The difference in effect between condition may reflect a resulting difference in the consistency of participant expectancies across conditions.

I thank the authors for addressing the issues I raised in the last review. However, there remain a couple of major inaccuracies which prevent the conclusions from following from both the presented data and existing evidence. There is also important information missing for Bayesian analyses. I will detail these below and provide some other brief comments which I hope will be useful.

1) On p.21 “If the effect of the hypnotic suggestion were driven by compliance in response to demand characteristics, social desirability would be expected to correlate with responses,”. This is incorrect. First, null relationships with social desirability measures do not provide evidence against demand characteristic effects (see e.g., Nichols & Maner, 2008). Second, the idea that response to imaginative suggestion is attributable to being controlled by a ‘hypnotist’ or being gullible or weak willed etc is a myth arising from the hypnotic context (e.g., see (Lynn et al., 2020.). As I stated in the last round of review, we employ the term ‘phenomenological control’ with the aim of avoiding misunderstandings of this kind which can arise from use of the term ‘suggestibility’. Response to imaginative suggestion is goal-directed and strategic. A null relationship between measures of social compliance and a given effect does not provide evidence against a cold control mechanism for that effect. These interpretations of social desirability results don’t hold and should be revised or removed.

We respectfully disagree with the reviewer. The reviewer seems to be misunderstanding what we’ve written and in particular the word ‘compliance’. We’re referring to simple compliance effects here, where participants overtly comply with the intended outcome without any actual changes in their experience. We’re not referring to suggestion effects, genuine changes in experience in response to the suggestion, or to what the author refers to as ‘phenomenological control’. This is precisely why we referred to “compliance” and not just demand characteristics, which can denote both simple compliance effects as well as genuine changes in experience. Similarly, we aren’t arguing for or against cold control theory – cold control theory isn’t relevant at all to this point. We’re not making any strong claim here – we’re only stating that a null correlation between social desirability and our observed effects (supported by Bayes factors) is consistent with the idea that the effects are not simple compliance effects. Indeed, this interpretation is consistent with the notion of phenomenological control and cold control theory (as well as other theories of hypnosis). We only base our conclusion on social desirability being representative of compliance, not all types of demand characteristics effects. Indeed, as the reviewer stated in a recent article (Corneille and Lush 2023), there are different strategies to meet the requirements of the situation, including behavioral compliance. Our conclusion here is that compliance is unlikely, and we think it is a reasonable conclusion.

Furthermore, we agree that response to suggestion is different from gullibility or being controlled by the hypnotist. These are well established results in the field. This has nothing to do with the quoted passage or our work. 

2) It is asserted that there was “no systematic expectation” (p.19) for the Lackner illusion condition. I do not consider the claim on p.12 to be sufficient evidence against expectancy differences to support this assertion (“No participant reported being aware that one of the conditions was a sham.”). Whether there is “systematic expectation” for the Lackner condition is an open question. I think it is likely that there is. My position is informed by evidence for hypothesis awareness in a measure similar to the pointing task here in another embodiment illusion procedure (“proprioceptive drift”): https://royalsocietypublishing.org/doi/10.1098/rsos.210911 . In any case, there is no evidence provided here to decide this matter (and the claim is contradicted in subsequent paragraphs). This assertion should be removed.

Our reasoning is based on the sham manipulation being similar to the baseline condition (at least on the perceptual task). If systematic expectation were present, there should be a significant difference in the sham condition relative to baseline, which was not the case. We simply disagree on the sham being a valid control. The reviewer has not pointed to a specific difference between the sham and Lackner illusion to support their view and so we have left this statement in.

3) “Robustness tests with a wider prior (a width of √2,i.e., twice the size of the original prior) reached similar conclusions and thus the report results do not seem to be highly dependent on the specific parameters chosen for the prior.”

As requested in the previous round, robustness regions should be reported (alongside each Bayes factor). The sentence above is not a sufficient alternative. Robustness regions allow the reader to interpret Bayes factors according to their own model of H1. Because the analysis employs default models of H1, which are uniformed by evidence, RRs are of particular importance.

We can see the merit of reporting robustness regions and we updated the manuscript accordingly. We were not able to compute robustness regions for the Covariate effects in repeated-measures ANOVAs examining condition effects on responses in the finger length perception task and line reaching task. However, we do not consider this to be problematic as these analyses are only used to reject the hypothesis that each covariate significantly influences the effect of the conditions. We also removed the insensitive comparisons between correlations in the “correlations” section (p.17). 

Other points:

• P.14, Ln 11: “By contrast”. Although this might be intended to only refer to a contrast in reference to sensitivity of Bayesian analysis, it could also be taken to mean a sensitive result contrasts meaningfully with an insensitive result. Because an insensitive result cannot be contrasted in this way (it provides no evidence for either H1 or H0 and is therefore uninformative), this second interpretation would not be valid. For this reason I recommend removing any reference to insensitive Bayes factors contrasting with sensitive Bayes factors.

We changed “by contrast” to “by comparison”.

• P.21 “When the suggested expectancies are unlikely for a participant, as might be expected for a low suggestible participant”

This claim lacks evidence. The relationship between expectancies and hypnotic responding does not indicate that low hypnotisables have particularly low expectancies (e.g., Benham et al, 2006).

This literature is more mixed than the reviewer implies, and some studies have indeed shown that low responsive participants do indeed have lower expectations (e.g., Lynn et al., 2008, Oxford handbook of hypnosis). On the other hand, we have several ongoing projects where the relationship between expectancy and response to suggestion shows heteroscedasticity, with low expectancies corresponding to no response and high expectancies corresponding to the whole range of responding. Nonetheless, this is not an important point for us, and we changed it for something less contentious (p21): “When the suggested experience is considered unlikely (low expectancy) by a participant, as might be the case for a difficult suggestion such as visual hallucination”

Attachment Submitted filename: Response to reviewers.docx Click here for additional data file.

# Peer review: Decision Letter 3

**DOI:** 10.1371/journal.pone.0291493.r007

**Authors:** Jane Elizabeth Aspell

28 Jul 2023

PONE-D-22-33125R3Hypnotic suggestion versus sensory modulation of bodily awarenessPLOS ONE

Dear Dr. Apelian,

Thank you for submitting your manuscript to PLOS ONE.  While one reviewer has (previously) recommended acceptance of the manuscript, the other has some remaining concerns, but the they think it should be a fairly straightforward task to address these by removing what they regard are unsupported claims and by qualifying some interpretations (some are addressed in response to reviewers but not in the manuscript itself). See the reviewer's response for details.

Please submit your revised manuscript by Sep 11 2023 11:59PM. If you will need more time than this to complete your revisions, please reply to this message or contact the journal office at plosone@plos.org. When you're ready to submit your revision, log on to https://www.editorialmanager.com/pone/ and select the 'Submissions Needing Revision' folder to locate your manuscript file.

Please include the following items when submitting your revised manuscript:

- A rebuttal letter that responds to each point raised by the academic editor and reviewer(s). You should upload this letter as a separate file labeled 'Response to Reviewers'.
- A marked-up copy of your manuscript that highlights changes made to the original version. You should upload this as a separate file labeled 'Revised Manuscript with Track Changes'.
- An unmarked version of your revised paper without tracked changes. You should upload this as a separate file labeled 'Manuscript'.

If you would like to make changes to your financial disclosure, please include your updated statement in your cover letter. Guidelines for resubmitting your figure files are available below the reviewer comments at the end of this letter.

If applicable, we recommend that you deposit your laboratory protocols in protocols.io to enhance the reproducibility of your results. Protocols.io assigns your protocol its own identifier (DOI) so that it can be cited independently in the future. For instructions see: https://journals.plos.org/plosone/s/submission-guidelines#loc-laboratory-protocols. Additionally, PLOS ONE offers an option for publishing peer-reviewed Lab Protocol articles, which describe protocols hosted on protocols.io. Read more information on sharing protocols at https://plos.org/protocols?utm_medium=editorial-email&utm_source=authorletters&utm_campaign=protocols.

We look forward to receiving your revised manuscript.

Kind regards,

Jane Elizabeth Aspell, PhD

Academic Editor

PLOS ONE

Journal Requirements:

Please review your reference list to ensure that it is complete and correct. If you have cited papers that have been retracted, please include the rationale for doing so in the manuscript text, or remove these references and replace them with relevant current references. Any changes to the reference list should be mentioned in the rebuttal letter that accompanies your revised manuscript. If you need to cite a retracted article, indicate the article’s retracted status in the References list and also include a citation and full reference for the retraction notice.

[Note: HTML markup is below. Please do not edit.]

Reviewers' comments:

Reviewer's Responses to Questions

**Comments to the Author**

1. If the authors have adequately addressed your comments raised in a previous round of review and you feel that this manuscript is now acceptable for publication, you may indicate that here to bypass the “Comments to the Author” section, enter your conflict of interest statement in the “Confidential to Editor” section, and submit your "Accept" recommendation.

Reviewer #2: (No Response)

**********

2. Is the manuscript technically sound, and do the data support the conclusions?

The manuscript must describe a technically sound piece of scientific research with data that supports the conclusions. Experiments must have been conducted rigorously, with appropriate controls, replication, and sample sizes. The conclusions must be drawn appropriately based on the data presented. 

Reviewer #2: Partly

**********

3. Has the statistical analysis been performed appropriately and rigorously? 

Reviewer #2: No

**********

4. Have the authors made all data underlying the findings in their manuscript fully available?

The PLOS Data policy requires authors to make all data underlying the findings described in their manuscript fully available without restriction, with rare exception (please refer to the Data Availability Statement in the manuscript PDF file). The data should be provided as part of the manuscript or its supporting information, or deposited to a public repository. For example, in addition to summary statistics, the data points behind means, medians and variance measures should be available. If there are restrictions on publicly sharing data—e.g. participant privacy or use of data from a third party—those must be specified.

Reviewer #2: Yes

**********

5. Is the manuscript presented in an intelligible fashion and written in standard English?

PLOS ONE does not copyedit accepted manuscripts, so the language in submitted articles must be clear, correct, and unambiguous. Any typographical or grammatical errors should be corrected at revision, so please note any specific errors here.

Reviewer #2: Yes

**********

6. Review Comments to the Author

Please use the space provided to explain your answers to the questions above. You may also include additional comments for the author, including concerns about dual publication, research ethics, or publication ethics. (Please upload your review as an attachment if it exceeds 20,000 characters)

Reviewer #2: The authors have addressed several of my comments. However, the main points I raised in the last review round have not been satisfactorily addressed (the first has been addressed in the reviewer response, but not in the manuscript).

1)

a) “ If this effect were driven by compliance in response to demand characteristics, social desirability would be expected to correlate with responses, at least in the hypnotic condition.”

As I noted previously, trait social desirability is not a good predictor of demand characteristic effects e.g., (https://www.tandfonline.com/doi/abs/10.3200/GENP.135.2.151-166), and a null relationship between social desirability and an effect does not provide evidence that an effect is not attributable to demand characteristics (including compliance). The authors' response treats this claim more cautiously, but this is not reflected in the manuscript.

b) The authors have clarified that that their claim about social desirability is only intended to refer to compliance effects, and not to cold control theory. However, this has not been clarified in the manuscript, and the passage can still be read as a rejection of cold control theory in favour of a predictive processing theory of hypnosis. The unintended rejection of cold control implied by the structure could be avoided by presenting any arguments about compliance separately, rather than sandwiched in between discussion of the two theories.

2) The assertion on p.19 that there was “no systematic” expectation for Lackner illusion conditions has not been removed as requested. The authors justify retaining it with the following argument:

“If systematic expectation were present, there should be a significant difference in the sham condition relative to baseline, which was not the case.”

This is incorrect. Whether or not a difference between baseline and sham condition would be expected to arise from expectancies depends on the direction of the expectancies. I repeat the point made in my previous review - the evidence presented does not support this strong claim regarding a central issue. The claim should be removed, or evidence provided to support it (e.g., instruction-based replication: https://doi.org/10.1525/collabra.82234)

3) RRs for Table 4,: it should be made explicit that all the RR values allow the full range of possible correlations, so the evidence for H0 is overstated.

To illustrate, the 95% CI for the correlation between the Lackner illusion perceptual effect and 'SDE' is between approximately r = -.10 and r = .40. I consider a .4 correlation to be far from evidence for no relationship.

Table 4 also does not meet minimum reporting standards as there is no measure of dispersion (SE, SD or 95% CI). While it is necessary to provide some measure of dispersion, I strongly recommend presenting 95% CIs (in addition to SD or SE) so that the reader can easily interpret the results and decide for themselves whether they agree with the presented interpretation.

Note - the data file does not contain DVs for some measures (e.g., BIDR). While these can be calculated, PLOS policy requires they be included.

**********

7. PLOS authors have the option to publish the peer review history of their article (what does this mean?). If published, this will include your full peer review and any attached files.

If you choose “no”, your identity will remain anonymous but your review may still be made public.

**Do you want your identity to be public for this peer review?** For information about this choice, including consent withdrawal, please see our Privacy Policy.

Reviewer #2: **Yes: **Peter Lush

**********

[NOTE: If reviewer comments were submitted as an attachment file, they will be attached to this email and accessible via the submission site. Please log into your account, locate the manuscript record, and check for the action link "View Attachments". If this link does not appear, there are no attachment files.]

While revising your submission, please upload your figure files to the Preflight Analysis and Conversion Engine (PACE) digital diagnostic tool, https://pacev2.apexcovantage.com/. PACE helps ensure that figures meet PLOS requirements. To use PACE, you must first register as a user. Registration is free. Then, login and navigate to the UPLOAD tab, where you will find detailed instructions on how to use the tool. If you encounter any issues or have any questions when using PACE, please email PLOS at figures@plos.org. Please note that Supporting Information files do not need this step.

# Author comment: Author response to Decision Letter 3

**DOI:** 10.1371/journal.pone.0291493.r008

23 Aug 2023

Response to reviewers was provided as an attached file for easier readability.

We thank the second reviewer for his additional comments. Our responses (in blue) are below, and we have also highlighted corresponding changes in the text.

Reviewer #2:

The authors have addressed several of my comments. However, the main points I raised in the last review round have not been satisfactorily addressed (the first has been addressed in the reviewer response, but not in the manuscript).

1)

a) “ If this effect were driven by compliance in response to demand characteristics, social desirability would be expected to correlate with responses, at least in the hypnotic condition.”

As I noted previously, trait social desirability is not a good predictor of demand characteristic effects e.g., (https://www.tandfonline.com/doi/abs/10.3200/GENP.135.2.151-166), and a null relationship between social desirability and an effect does not provide evidence that an effect is not attributable to demand characteristics (including compliance). The authors' response treats this claim more cautiously, but this is not reflected in the manuscript.

The evidence is actually mixed concerning the link between social desirability and demand characteristics, with some articles showing a positive correlation (https://link.springer.com/article/10.1007/s00520-023-07761-z). Note that we’re not claiming that this effect is thus not attributable to demand characteristics – only that an explanation in terms of basic compliance is rendered less likely. We have now amended the text as follows (p. 23): “If this effect were driven by compliance in response to the perceived requirements of the situation, social desirability would be expected to correlate with responses, at least in the hypnotic condition. Insofar as we observed a null correlation for this association, this suggests that our observed effects are unlikely to be driven by compliance, but we cannot rule out the possibility of broader effects related to demand characteristics.”

b) The authors have clarified that that their claim about social desirability is only intended to refer to compliance effects, and not to cold control theory. However, this has not been clarified in the manuscript, and the passage can still be read as a rejection of cold control theory in favour of a predictive processing theory of hypnosis. The unintended rejection of cold control implied by the structure could be avoided by presenting any arguments about compliance separately, rather than sandwiched in between discussion of the two theories.

We think this section is fairly clear since we’re clearly writing about compliance, which is a very distinct mechanism from that proposed by cold control theory. Nevertheless, we’re happy to move this to avoid any confusion. We moved this section later in the discussion (p.23) 

2) The assertion on p.19 that there was “no systematic” expectation for Lackner illusion conditions has not been removed as requested. The authors justify retaining it with the following argument:

“If systematic expectation were present, there should be a significant difference in the sham condition relative to baseline, which was not the case.”

This is incorrect. Whether or not a difference between baseline and sham condition would be expected to arise from expectancies depends on the direction of the expectancies. I repeat the point made in my previous review - the evidence presented does not support this strong claim regarding a central issue. The claim should be removed, or evidence provided to support it (e.g., instruction-based replication: https://doi.org/10.1525/collabra.82234)

As suggested by the reviewer, we gathered data from an online survey. We report the results of this survey in the article p.10-11: 

To ensure that expectations were similar between the sham manipulation and the Lackner illusion manipulation, we surveyed a group of 72 participants (online) that did not know of the Lackner illusion. Participants were described the manipulations and measurements with text and images, but the expected effect of the manipulation was not mentioned similarly to the study. Manipulations were labelled as “illusion 1” (sham) and “illusion 2” (Lackner illusion) for participants. They were then asked to estimate if the manipulation, compared to baseline, would make a person receiving the illusion “overshoot, pointing past the lines”, “correctly point to the lines”, or “undershoot, being short of reaching the lines”. This was done for both manipulations in the same order as the experiment, with the sham manipulation first then the Lackner illusion manipulation. Similar questions were asked about the effects in the finger length perception task with options: “perceive the index finger as shorter”, “perceive the index finger as normal”, “perceive the index finger as longer”. These analyses reveal that for the line reaching task expectancies are relatively balanced (no significant difference from .5) between manipulations, proportion P=.465 was not significantly different from .5 with p=.635, B=0.13 (See Table 1). However, this was not the case for the perceptual task where the sham manipulation was expected to lead to perceptual elongation more than the Lackner illusion, proportion P=.333 was significantly different from .5 with p=.007, B=4.45 (See Table 1). Overall, we are confident that the sham manipulation played its role as a control condition.

3) RRs for Table 4,: it should be made explicit that all the RR values allow the full range of possible correlations, so the evidence for H0 is overstated.

To illustrate, the 95% CI for the correlation between the Lackner illusion perceptual effect and 'SDE' is between approximately r = -.10 and r = .40. I consider a .4 correlation to be far from evidence for no relationship.

Table 4 also does not meet minimum reporting standards as there is no measure of dispersion (SE, SD or 95% CI). While it is necessary to provide some measure of dispersion, I strongly recommend presenting 95% CIs (in addition to SD or SE) so that the reader can easily interpret the results and decide for themselves whether they agree with the presented interpretation.

We added the 95% CIs, as recommended. The comment on RR and evidence for H0 being overstated comes down to the prior distribution selected (a stretched beta distribution). However, we put a reminder under Table 4 (now Table 5) to help the reader in interpreting RR (p.19): Bs and RR are derived from a stretched beta distribution prior with width 1.0

Note - the data file does not contain DVs for some measures (e.g., BIDR). While these can be calculated, PLOS policy requires they be included.

This has been changed in the data file.

Attachment Submitted filename: Response to reviewers.docx Click here for additional data file.

# Editor report: Decision Letter 4

**DOI:** 10.1371/journal.pone.0291493.r009

**Authors:** Jane Elizabeth Aspell

31 Aug 2023

Hypnotic suggestion versus sensory modulation of bodily awareness

PONE-D-22-33125R4

Dear Dr. Apelian,

We’re pleased to inform you that your manuscript has been judged scientifically suitable for publication and will be formally accepted for publication once it meets all outstanding technical requirements.

Within one week, you’ll receive an e-mail detailing the required amendments. When these have been addressed, you’ll receive a formal acceptance letter and your manuscript will be scheduled for publication.

An invoice for payment will follow shortly after the formal acceptance. To ensure an efficient process, please log into Editorial Manager at http://www.editorialmanager.com/pone/, click the 'Update My Information' link at the top of the page, and double check that your user information is up-to-date. If you have any billing related questions, please contact our Author Billing department directly at authorbilling@plos.org.

If your institution or institutions have a press office, please notify them about your upcoming paper to help maximize its impact. If they’ll be preparing press materials, please inform our press team as soon as possible -- no later than 48 hours after receiving the formal acceptance. Your manuscript will remain under strict press embargo until 2 pm Eastern Time on the date of publication. For more information, please contact onepress@plos.org.

Kind regards,

Jane Elizabeth Aspell, PhD

Academic Editor

PLOS ONE

# Editor report: Acceptance letter

**DOI:** 10.1371/journal.pone.0291493.r010

**Authors:** Jane Elizabeth Aspell

4 Sep 2023

PONE-D-22-33125R4 

Hypnotic suggestion versus sensory modulation of bodily awareness 

Dear Dr. Apelian:

I'm pleased to inform you that your manuscript has been deemed suitable for publication in PLOS ONE. Congratulations! Your manuscript is now with our production department. 

If your institution or institutions have a press office, please let them know about your upcoming paper now to help maximize its impact. If they'll be preparing press materials, please inform our press team within the next 48 hours. Your manuscript will remain under strict press embargo until 2 pm Eastern Time on the date of publication. For more information please contact onepress@plos.org.

If we can help with anything else, please email us at plosone@plos.org. 

Thank you for submitting your work to PLOS ONE and supporting open access. 

Kind regards, 

PLOS ONE Editorial Office Staff

on behalf of

Dr. Jane Elizabeth Aspell 

Academic Editor

PLOS ONE
